# Trends in Population‐Level Mortality Rates for Deaths With Both Alzheimer's Disease and Cancer Recorded on Death Certificates Among U.S. Adults Aged ≥ 65 Years, 1999–2023

**DOI:** 10.1002/nop2.70897

**Published:** 2026-10-06

**Authors:** Xiangxiang Zhang, Yu‐Jun Xiong, Tian Lv, Yiqiao Chen

**Affiliations:** ^1^ Department of Psychiatry, Shaoxing Seventh People's Hospital Shaoxing Zhejiang China; ^2^ Department of Gastroenterology, Beijing Hospital, National Centre For Gerontology, National Clinical Research Centre for Gerontology, the Key Laboratory of Geriatrics of NHC Institute of Geriatric Medicine, Chinese Academy of Medical Sciences Beijing China; ^3^ Department of Neurology Zhuji Affiliated Hospital of Wenzhou Medical University Zhuji China; ^4^ Department of Neurology Qingtian People's Hospital Zhejiang China

**Keywords:** age‐adjusted mortality rate, Alzheimer's disease, annual percent change, cancer, mortality trends

## Abstract

**Aim:**

To examine temporal trends and demographic and geographic variation in population‐level rates of deaths with both Alzheimer's disease (AD) and cancer recorded on death certificates among U.S. adults aged ≥ 65 years from 1999 to 2023.

**Design:**

A population‐based descriptive time‐trend study using multiple‐cause‐of‐death data.

**Data Sources:**

National mortality data from the CDC WONDER (1999–2023).

**Review Methods:**

Death certificates for U.S. residents aged ≥ 65 years recording both AD (G30) and malignant neoplasms (C00–C97) were included. Age‐adjusted mortality rates (AAMRs) were standardized to the 2000 U.S. population; age‐group analyses used age‐specific crude mortality rates. Joinpoint regression estimated annual percent changes (APCs) and average annual percent changes (AAPCs), with analyses stratified by demographic and geographic characteristics.

**Results:**

Annual deaths increased from 4764 in 1999 to 6240 in 2023, while the overall AAMR declined from 13.96 to 12.05 per 100,000 (AAPC −0.73%, 95% CI −1.39 to −0.06). Significant declines occurred among males, adults aged 75 –84 years, non‐Hispanic Black populations, and residents of the Northeast and Midwest, whereas a significant increase occurred among Hispanic populations. Full‐period trends among females and non‐Hispanic Other populations were not statistically significant. Neither metropolitan nor nonmetropolitan areas showed a significant trend during 1999–2020.

**Conclusion:**

The overall age‐adjusted death‐certificate mortality rate declined modestly despite increasing annual death counts, with descriptive variation across demographic and geographic groups.

**Implications for the Profession and/or Patient Care:**

The findings highlight the relevance of coordinated geriatric and oncology nursing assessment and care planning for older adults with complex health needs. Nurses may play an important role in identifying cognitive, functional, treatment‐related, and caregiver needs and facilitating multidisciplinary care. These descriptive findings identify areas for clinical attention but do not demonstrate the effectiveness of specific nursing interventions.

**What Problem Did the Study Address?:**

Limited evidence exists on long‐term population‐level trends in deaths with both AD and cancer recorded on death certificates.

**What Were the Main Findings?:**

The overall age‐adjusted mortality rate declined modestly while annual death counts increased. Subgroup‐specific trends varied, but no formal between‐group comparisons were performed.

**Where and on Whom Will the Research Have an Impact?:**

The findings are relevant to clinicians, policymakers, and public health practitioners involved in the care of older adults with multimorbidity, particularly in underserved populations.

**Reporting Method:**

This study adheres to the STROBE (Strengthening the Reporting of Observational Studies in Epidemiology) guidelines.

**Patient or Public Contribution:**

No Patient or Public Contribution. This study used publicly available, de‐identified mortality data and did not involve direct patient or public participation.

## 1 Introduction

Alzheimer's disease represents a major and growing global public health challenge. According to the most recent estimates from the Global Burden of Disease Study 2021, approximately 57 million individuals were living with dementia worldwide in 2021, with Alzheimer's disease being the most common subtype (Liu et al. 2025). This resulted in an estimated 8.02 million deaths and 25.38 million disability‐adjusted life years (Xiaopeng et al. 2025). Over recent decades, the absolute number of dementia cases has more than doubled compared to 1990, a rise driven largely by population aging and growth. While age‐standardized incidence and mortality rates have remained relatively stable, the sharp increase in absolute numbers underscores the escalating population‐level burden of Alzheimer's disease and other dementias worldwide (Hao and Chen 2025). As a progressive neurodegenerative disease, Alzheimer's imposes substantial personal, social, and economic burdens, a challenge that intensifies with global demographic aging.

Concurrently, cancer persists as one of the most burdensome diseases globally, with its incidence and mortality continuing to rise. The latest global cancer statistics in 2022 reported nearly 20 million new cancer cases and approximately 9.7 million cancer‐related deaths annually (Bray et al. 2024). Among the various cancer types, lung, breast (in women), colorectal, prostate, and stomach cancers remain the most commonly diagnosed and are leading causes of cancer mortality (Mattiuzzi and Lippi 2019). The global burden of cancer is anticipated to increase further in the coming decades, propelled by demographic shifts, population aging, and changing exposures to risk factors (Li et al. 2024). This persistent and rising burden underscores the ongoing challenge cancer poses to global health systems and highlights the critical need for effective prevention, early detection, and management strategies at scale.

Although Alzheimer's disease and cancer are often studied independently, there is growing interest in their potential comorbidity and interaction. An increasing population of older adults surviving cancer may lead to a higher proportion of patients living with both cancer and neurodegenerative conditions like Alzheimer's disease, posing complex challenges for clinical management, prognosis, and quality of life (Prathap et al. 2024; Wennberg et al. 2023). Furthermore, shared risk factors—including aging, metabolic disorders, and systemic inflammation—might predispose individuals to both neurodegeneration and malignancy, suggesting potential underlying biological links (Zabłocka et al. 2021). Consequently, the coexistence of Alzheimer's disease and cancer likely represents a vulnerable subpopulation with distinct clinical needs and prognostic profiles. Despite this potential overlap, population‐level trends in deaths with both conditions recorded on death certificates remain insufficiently characterized.

To address this evidence gap, we analysed national multiple‐cause‐of‐death data from 1999 to 2023 to characterize population‐level rates of deaths with both Alzheimer's disease and cancer recorded on death certificates among U.S. adults aged ≥ 65 years. We examined temporal patterns across demographic and geographic groups. These estimates describe death‐certificate‐based mortality patterns in the general older population; They do not estimate individual mortality risk, case fatality, or survival among people living with both conditions. Generating such evidence is critical to inform clinical decision‐making, resource allocation, and public health policy, particularly in the context of aging populations and the rising prevalence of multimorbidity.

## 2 Materials and Methods

### 2.1 Study Design, Data Sources, and Case Definition

We conducted a population‐based descriptive time‐trend study using publicly available, de‐identified aggregate mortality data from the Centres for Disease Control and Prevention Wide‐ranging Online Data for Epidemiologic Research (CDC WONDER) platform (Dort et al. 2025). Data were extracted on June 20, 2026. We used the Multiple Cause of Death, 1999–2020 dataset with bridged‐race categories for deaths occurring during 1999–2020 and the Multiple Cause of Death, 2018–2023 dataset with single‐race categories for deaths occurring during 2021–2023. Each calendar year was included once, without duplication across the two extracts.

Eligible deaths occurred among U.S. residents aged ≥ 65 years and had both Alzheimer's disease (AD; ICD‐10 G30) and malignant neoplasms (ICD‐10 C00–C97) recorded on the same death certificate. Within the ‘Multiple Cause of Death’ ICD‐10 selection fields, malignant neoplasms were entered in the first selection box and AD in the second, with the selections combined using the AND operator. The underlying cause‐of‐death field was retained as ‘All Causes,’ with no additional restriction. Either condition could therefore appear as the underlying or a contributing cause, and neither was required to occupy a specific position on the death certificate. This definition identifies deaths with both conditions recorded rather than a cohort of individuals living with both diagnoses (Qadri et al. 2025). Analyses used the aggregate data returned by CDC WONDER under the specified query criteria; no additional exclusions for missing demographic information were applied by the investigators after extraction.

### 2.2 Data Extraction and Subgroup Analyses

Annual death counts, corresponding population estimates, mortality rates, standard errors, and 95% confidence intervals (CIs) were extracted from CDC WONDER. Analyses were stratified by age (65–74, 75–84, and ≥ 85 years), sex, race and ethnicity (Hispanic, non‐Hispanic White, non‐Hispanic Black, and non‐Hispanic Other), U.S. census region (Northeast, Midwest, South, and West), and state. All rates used the population denominator corresponding to the year and demographic or geographic group being analysed (Shahid et al. 2025). For race‐ and ethnicity‐specific analyses, categories were harmonized into Hispanic, non‐Hispanic White, non‐Hispanic Black, and non‐Hispanic Other groups. The non‐Hispanic Other category represented non‐Hispanic racial categories other than White and Black available within the respective CDC WONDER extracts. Because the 1999–2020 bridged‐race and 2021–2023 single‐race systems differ in the classification of some racial identities, particularly multiracial populations, the composition of the non‐Hispanic Other category cannot be assumed to be identical across the entire study period. Accordingly, temporal findings for this heterogeneous category were interpreted cautiously.

Urbanization was classified using the 2013 National Centre for Health Statistics Urban–Rural Classification Scheme for Counties. Large central, large fringe, medium, and small metropolitan counties were grouped as metropolitan, whereas micropolitan and noncore counties were grouped as nonmetropolitan. Urbanization‐stratified analyses were restricted to 1999–2020, the period covered by the urbanization‐specific data extracted for this study. Accordingly, all urbanization‐specific death counts, rates, and trend estimates refer to this interval.

### 2.3 Statistical Analysis

The numerator comprised deaths meeting the specified death‐certificate case definition, and the denominator was the corresponding U.S. resident population aged ≥ 65 years, or the relevant demographic or geographic subgroup. Rates were expressed per 100,000 population. Age‐adjusted mortality rates (AAMRs) were computed by direct standardization based on the year 2000 U.S. standard population. Analyses of the 65–74, 75–84, and ≥ 85‐year age groups used age‐specific crude mortality rates. These measures describe population‐level rates of deaths with both conditions recorded on death certificates; they do not estimate individual mortality risk, case fatality, or survival among people living with both diagnoses.

Temporal trends were evaluated using the Joinpoint Regression Program (version 5.4.0.0; National Cancer Institute). For the primary analyses, log‐linear models were fitted using the grid search method, with a minimum of 0 and a maximum of 4 joinpoints. The minimum number of observations from a joinpoint to either end of the series was 2, excluding the joinpoint when it coincided with an observation. The minimum number of observations between two joinpoints was also 2, excluding the joinpoints when they coincided with observations. Model selection was performed using Monte Carlo permutation tests with 4999 randomly permuted datasets. The “Standard Error” option was selected using the standard errors of the corresponding mortality rates obtained from CDC WONDER, with uncorrelated errors assumed.

Annual percent changes (APCs) were estimated for individual fitted segments, whereas average annual percent changes (AAPCs) summarized trends over specified intervals. The 95% CIs for AAPCs were calculated using the parametric method. Primary AAPCs summarized the full 1999–2023 study period, whereas urbanization‐specific AAPCs summarized 1999–2020. A sensitivity analysis restricted the observation period to 1999–2019 to examine whether the direction of the estimated trends was maintained when the pandemic years were excluded. This comparison involved overlapping observation periods and was not interpreted as an estimate of the causal effect of the COVID‐19 pandemic. To assess the potential influence of the transition from bridged‐race to single‐race mortality data, we conducted a sensitivity analysis using the overlapping years 2018–2020, for which estimates were available from both CDC WONDER Multiple Cause of Death datasets. Race‐ and ethnicity‐specific AAMRs were extracted using identical age and cause‐of‐death criteria from the 1999–2020 bridged‐race dataset and the 2018–2024 single‐race dataset. For harmonizable racial and ethnic groups, we compared annual AAMRs between the two sources and calculated absolute and relative differences. Because multiracial decedents and Pacific Islander records are classified differently across the two systems, comparisons involving the heterogeneous non‐Hispanic Other category were interpreted cautiously.

An APC or AAPC was interpreted as indicating an increasing or decreasing trend at the nominal 5% significance level when its 95% CI excluded zero. Otherwise, no statistically significant change was identified. State‐level and other stratified analyses were considered exploratory. *p* values and 95% CIs were not adjusted for multiple comparisons across these analyses, and nominally significant findings were therefore interpreted cautiously. Statistical significance within one subgroup, but not another, was not interpreted as evidence of a statistically significant difference between the subgroup trends. Mortality rates and trend estimates that could not be calculated because of insufficient or unavailable data are identified as NA in the corresponding figures and Tables S1–S4.

## 3 Results

### 3.1 Overall Characteristics

During 1999–2023, 143,052 deaths with both AD and cancer recorded on death certificates were identified among U.S. residents aged ≥ 65 years (Figure 1A–D; Table 1). Annual death counts increased from 4764 in 1999 to 6240 in 2023. The overall AAMR decreased from 13.96 (95% CI 13.57 to 14.36) to 12.05 (95% CI 11.75 to 12.35) per 100,000, corresponding to a statistically significant AAPC of −0.73% (95% CI −1.39 to −0.06).

**FIGURE 1:**
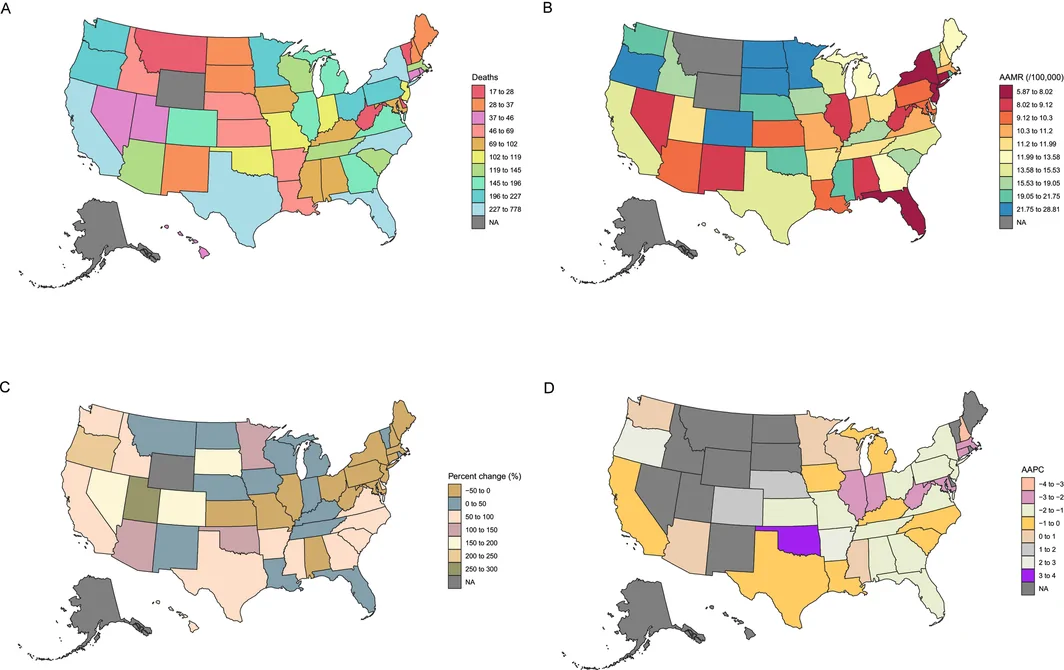
State‐specific patterns in deaths with both Alzheimer's disease and cancer recorded on death certificates among U.S. adults aged ≥ 65 years, 1999–2023: (A) death counts; (B) age‐adjusted mortality rates (AAMRs); (C) percentage change; and (D) average annual percent change (AAPC) in AAMRs. Rates are expressed per 100,000 persons in the corresponding general population aged ≥ 65 years.

**TABLE 1:** Trends in Mortality and Age‐Adjusted Mortality Rates for Cancers and Alzheimer's disease in 1999 and 2023.

Characteristics	Deaths			AAMR		
	1999	2023	Percent Change	1999	2023	AAPC (95% CI)
Overall	4764	6240	30.98	13.96 (13.57 – 14.36)	12.05 (11.75 – 12.35)	−0.73 (−1.39 – −0.06)*
Sex						
Female	2647	3357	26.82	11.79 (11.34 – 12.24)	10.82 (10.46 – 11.19)	−0.55 (−1.20 – 0.10)
Male	2117	2883	36.18	18.20 (17.41 – 18.99)	13.75 (13.25 – 14.26)	−1.17 (−2.09 – −0.25)*
Census Region						
Northeast	927	857	−7.55	12.45 (11.65 – 13.25)	8.64 (8.06 – 9.22)	−1.46 (−2.19 – −0.73)*
Midwest	1330	1387	4.29	15.84 (14.99 – 16.69)	12.64 (11.97 – 13.31)	−0.94 (−1.61 – −0.27)*
South	1589	2290	44.12	13.46 (12.80 – 14.13)	11.74 (11.26 – 12.22)	−0.70 (−1.54 – 0.15)
West	918	1706	85.84	13.94 (13.04 – 14.84)	14.70 (14.00 – 15.40)	0.40 (−0.52 – 1.33)
Races						
Hispanic	75	437	482.67	5.93 (4.66 – 7.45)	9.77 (8.85 – 10.69)	2.58 (0.93 – 4.25)*
NH Black	422	468	10.9	16.35 (14.79 – 17.91)	10.56 (9.59 – 11.53)	−1.69 (−3.09 – −0.26)*
NH White	4220	5090	20.62	14.21 (13.78 – 14.64)	12.85 (12.50 – 13.21)	−0.56 (−1.25 – 0.13)
NH Other	47	245	421.28	4.45 (3.00 – 6.35)	6.78 (5.92 – 7.65)	0.73 (−1.35 – 2.86)
Urbanization						
Metropolitan	3719	NA	NA	13.55 (13.11 – 13.99)	12.70 (12.36 – 13.03)	−0.48 (−1.00 – 0.04)
Nonmetropolitan	1045	NA	NA	15.52 (14.58 – 16.47)	14.77 (13.96 – 15.59)	−0.37 (−1.06 – 0.32)
Age groups						
65–74 years	364	504	38.46	1.98 (1.77 – 2.18)	1.45 (1.33 – 1.58)	−1.03 (−2.31 – 0.26)
75–84 years	1951	2216	13.58	15.96 (15.25 – 16.67)	12.06 (11.56 – 12.57)	−1.26 (−2.10 – −0.42)*
85+ years	2449	3520	43.73	58.95 (56.62 – 61.29)	56.82 (54.94 – 58.70)	−0.20 (−1.00 – 0.60)

Abbreviations: AAMR, Age adjusted mortality rate; AAPC, average annual percentage change; CI, confidence interval; NH, non‐Hispanic.

* Indicates an AAPC whose 95% confidence interval excludes zero at the nominal 5% significance level. No adjustment was made for multiple comparisons across stratified analyses.

^#^ Urbanization‐specific endpoint rates refer to 2020, and the corresponding AAPCs were calculated over 1999‐2020.

^##^ Rates for the age groups are age‐specific crude mortality rates, expressed per 100,000 persons in the corresponding age group. APCs and AAPCs for these groups were estimated from the age‐specific crude rates. Rates for all other categories are age‐adjusted mortality rates standardized to the 2000 U.S. standard population.

^###^ The large percentage increases in death counts among Hispanic adults (482.67%) and non‐Hispanic other adults (640.62%) should be interpreted in the context of the small 1999 baseline counts of 75 and 32 deaths, respectively; these percentages reflect changes in death counts, not mortality rates.

### 3.2 Sex‐Stratified Analyses

Over the full 1999–2023 period, males showed a statistically significant decline in mortality (AAPC −1.17%, 95% CI −2.09 to −0.25), whereas the estimated decline among females was not statistically significant (AAPC −0.55%, 95% CI −1.20 to 0.10). Joinpoint analyses further identified several shorter temporal segments within each sex (Figure 2; Table S2). Among females, a total of 79,117 deaths with both AD and cancer recorded on death certificates were identified during the study period. Joinpoint regression identified a multi‐phase temporal trajectory for female mortality. An initial increase was observed from 1999 to 2006, with an APC of 1.69% (95% CI 0.79 to 2.59). This was followed by a pronounced decline between 2006 and 2014 (APC –4.33%, 95% CI –5.18 to −3.48). Subsequently, mortality exhibited a significant upward trend from 2014 to 2021 (APC 3.11%, 95% CI 2.01 to 4.23), and the final segment, 2021–2023, had a negative APC that was not statistically significant (−5.33%, 95% CI −10.95 to 0.64).

**FIGURE 2:**
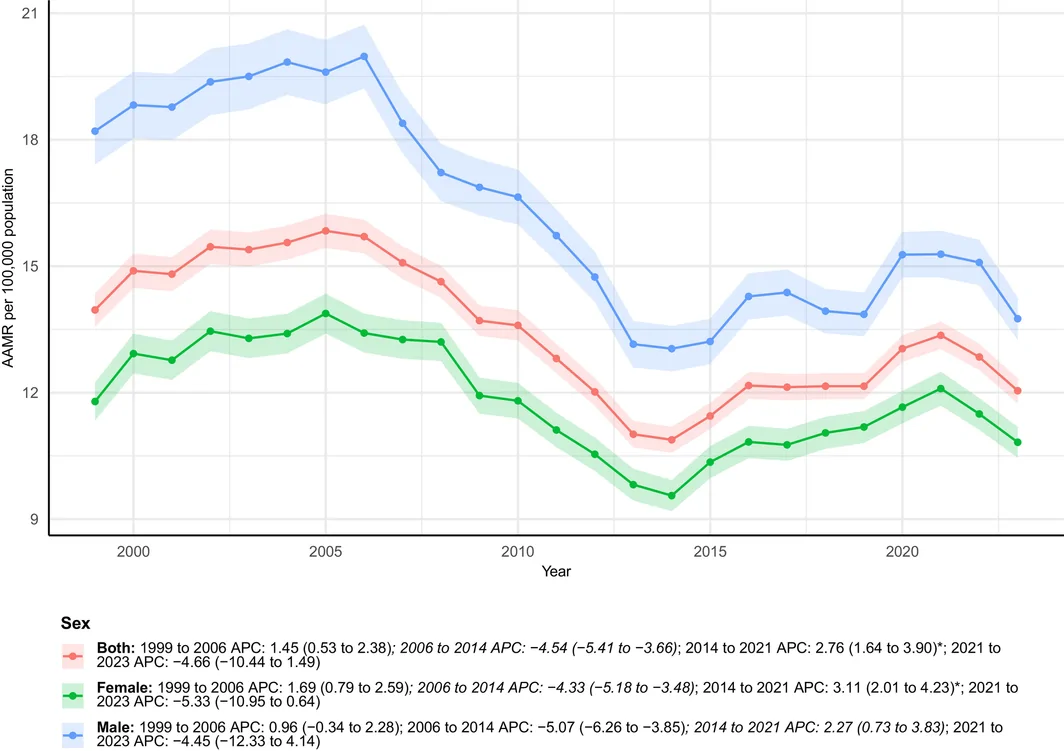
Age‐adjusted mortality rates (AAMRs) for deaths with both Alzheimer's disease and cancer recorded on death certificates among U.S. adults aged ≥ 65 years, stratified by sex, 1999–2023. Rates are expressed per 100,000 persons in the corresponding sex‐specific general population aged ≥ 65 years. Solid lines represent estimated mortality trends, and shaded areas indicate the corresponding 95% confidence intervals. Abbreviations: AAMR, age‐adjusted mortality rate; AAPC, average annual percent change.

Among males, 63,935 deaths were recorded over the same period. The mortality trend for males also demonstrated distinct phases. A non‐significant increase occurred from 1999 to 2006 (APC 0.96%, 95% CI –0.34 to 2.28), followed by a substantial reduction between 2006 and 2014 (APC –5.07%, 95% CI –6.26 to −3.85). This was succeeded by a period of moderate increase from 2014 to 2021 (APC 2.27%, 95% CI 0.73 to 3.83). The final segment from 2021 to 2023 showed a non‐significant decline (APC –4.45%, 95% CI –12.33 to 4.14).

### 3.3 Age‐Stratified Analyses

Age‐specific crude mortality rates were examined for the 65–74, 75–84, and ≥ 85‐year age groups during 1999–2023 (Figure 3; Tables S2–S4). All three groups had negative full‐period AAPC point estimates, but only the 75–84‐year group showed a statistically significant decline. Joinpoint regression identified periods of increasing and decreasing rates within each age group.

**FIGURE 3:**
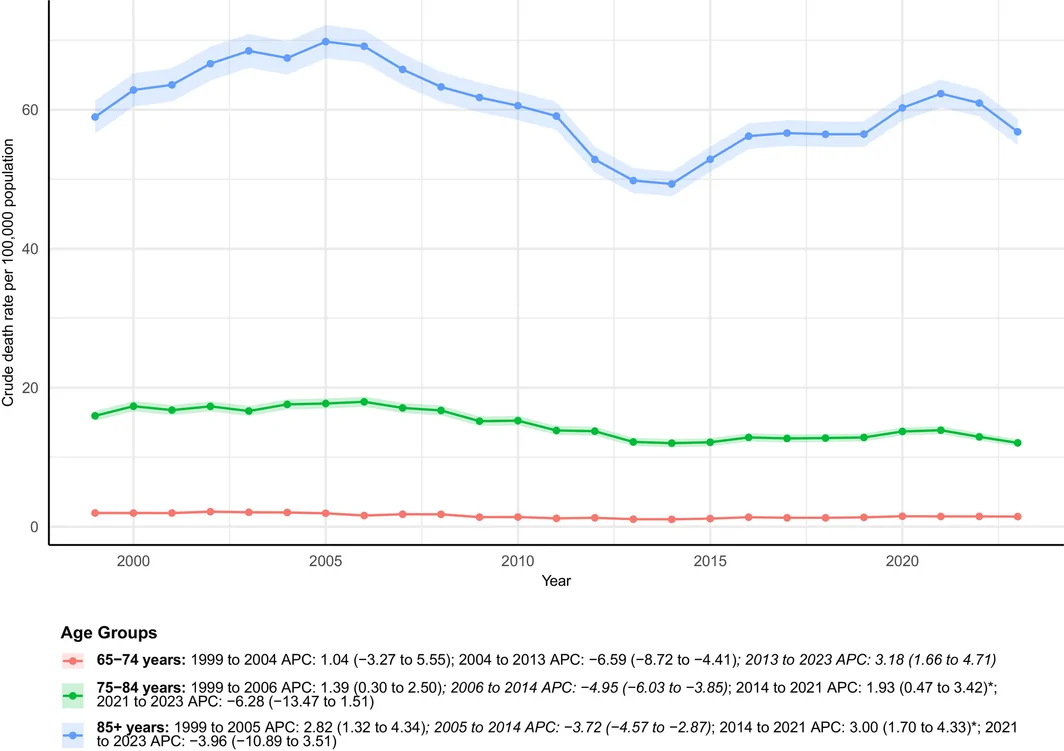
Age‐specific crude mortality rates for deaths with both Alzheimer's disease and cancer recorded on death certificates among U.S. adults aged ≥ 65 years, stratified by age group, 1999–2023. Rates are expressed per 100,000 persons in the corresponding general‐population age group. Solid lines represent estimated mortality trends, and shaded areas indicate the corresponding 95% confidence intervals. Abbreviation: AAPC, average annual percent change.

Within the 65–74 year age group, 9272 deaths were recorded. The age‐specific crude mortality rate decreased from 1.98 (95% confidence interval, 1.77 to 2.18) in 1999 to 1.45 (95% CI, 1.33 to 1.58) in 2023. The overall average annual percent change (AAPC) of −1.03% (95% CI, −2.31 to 0.26) was not statistically significant. Joinpoint analysis delineated three distinct temporal segments: no statistically significant trend during 1999–2004 (annual percent change [APC] 1.04%, 95% CI −3.27 to 5.55), followed by a pronounced decline from 2004 to 2013 (APC −6.59%, 95% CI −8.72 to −4.41), and a subsequent increase from 2013 to 2023 (APC 3.18%, 95% CI 1.66 to 4.71).

Among individuals aged 75 –84 years, 51,242 deaths occurred. The age‐specific crude mortality rate decreased from 15.96 (95% CI, 15.25 to 16.67) in 1999 to 12.06 (95% CI, 11.56 to 12.57) in 2023, corresponding to a statistically significant AAPC of −1.26% (95% CI, −2.10 to −0.42). Trend analysis identified an initial increase from 1999 to 2006 (APC 1.39%, 95% CI 0.30 to 2.50), followed by a pronounced decline during 2006–2014 (APC −4.95%, 95% CI −6.03 to −3.85). Mortality subsequently rose between 2014 and 2021 (APC 1.93%, 95% CI 0.47 to 3.42), before shifting to a nonsignificant decrease from 2021 to 2023 (APC −6.28%, 95% CI −13.47 to 1.51).

For patients aged 85 years and older, 82,538 deaths were documented. The age‐specific crude mortality rate decreased from 58.95 (95% CI, 56.62 to 61.29) to 56.82 (95% CI, 54.94 to 58.70) over the study period, with a nonsignificant AAPC of −0.20% (95% CI, −1.00 to 0.60). Joinpoint modelling indicated a significant initial increase from 1999 to 2005 (APC 2.82%, 95% CI 1.32 to 4.34), followed by a sustained decline during 2005–2014 (APC −3.72%, 95% CI −4.57 to −2.87). This was followed by a significant upward trend between 2014 and 2021 (APC 3.00%, 95% CI 1.70 to 4.33), and a final nonsignificant downward phase from 2021 to 2023 (APC −3.96%, 95% CI −10.89 to 3.51).

### 3.4 Regional‐Stratified Analyses

#### 3.4.1 Census Regions Stratified

Population‐level rates of deaths with both AD and cancer recorded on death certificates varied descriptively across U.S. census regions (Figure 4). The total number of deaths was highest in the South (*n* = 48,389), followed by the West (*n* = 36,533), the Midwest (*n* = 35,260), and the Northeast (*n* = 22,870).

**FIGURE 4:**
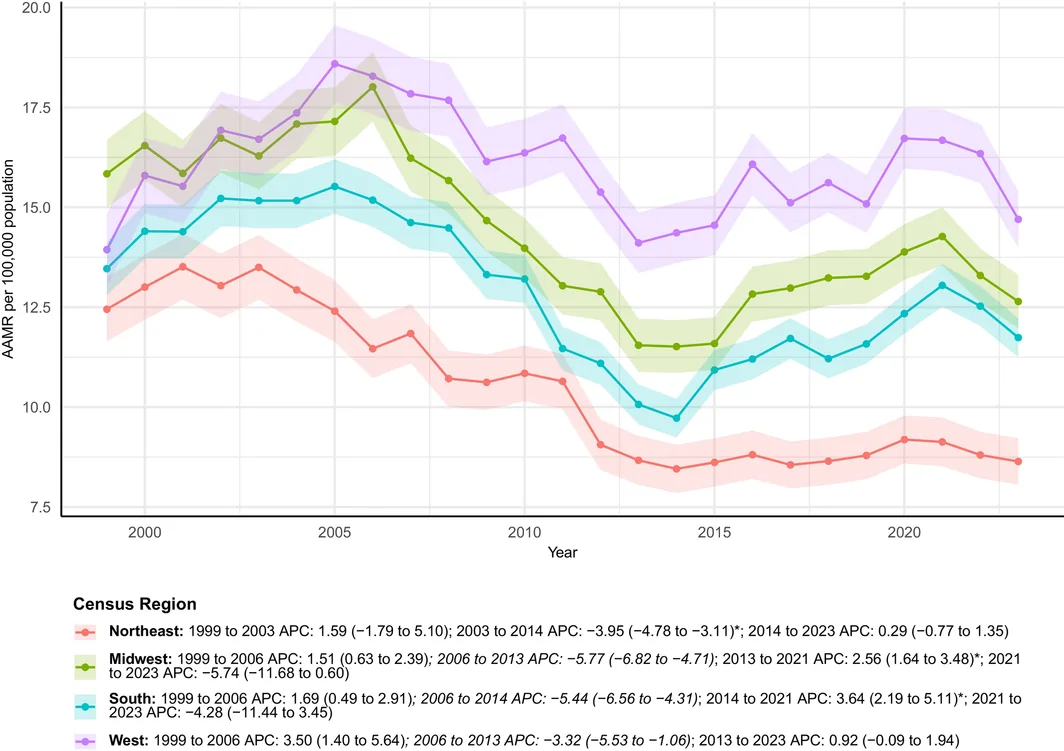
Age‐adjusted mortality rates (AAMRs) for deaths with both Alzheimer's disease and cancer recorded on death certificates among U.S. adults aged ≥ 65 years, stratified by census region, 1999–2023. Rates are expressed per 100,000 persons in the corresponding regional general population aged ≥ 65 years. Solid lines represent estimated mortality trends, and shaded areas indicate the corresponding 95% confidence intervals. Abbreviations: AAMR, age‐adjusted mortality rate; AAPC, average annual percent change.

In the Northeast, deaths modestly declined from 927 in 1999 to 857 in 2023, a reduction of 7.55%. Concurrently, the AAMR decreased from 12.45 to 8.64 per 100,000, with a statistically significant AAPC of −1.46% (95% CI, −2.19 to −0.73). Joinpoint analysis identified three distinct temporal phases: a non‐significant increase from 1999 to 2003 (APC 1.59%), a marked decline from 2003 to 2014 (APC −3.95%), and no statistically significant trend during 2014–2023 (APC 0.29%).

The Midwest witnessed a slight increase in deaths from 1330 to 1387 (4.29%), while the AAMR fell from 15.84 to 12.64 per 100,000, corresponding to a significant AAPC of −0.94% (95% CI, −1.61 to −0.27). The temporal trend consisted of an initial increase from 1999 to 2006 (APC 1.51%), followed by a sharp decline from 2006 to 2013 (APC −5.77%), a subsequent significant rebound from 2013 to 2021 (APC 2.56%), and a nonsignificant negative APC during 2021–2023 (−5.74%).

Despite a substantial rise in deaths in the South from 1589 to 2290 (44.12%), the AAMR declined from 13.46 to 11.74 per 100,000. The overall AAPC was −0.70% (95% CI, −1.54 to 0.15), which was not statistically significant. The mortality trend showed an initial increase from 1999 to 2006 (APC 1.69%), a pronounced decrease from 2006 to 2014 (APC −5.44%), followed by a period of significant increase from 2014 to 2021 (APC 3.64%), and a slight decline thereafter from 2021 to 2023 (APC −4.28%).

In the West, annual death counts increased from 918 in 1999 to 1706 in 2023, an increase of 85.84%. In contrast to other regions, the AAMR showed a mild increase from 13.94 to 14.70 per 100,000, yielding a non‐significant AAPC of 0.40% (95% CI, −0.52 to 1.33). The temporal pattern was characterized by a significant initial increase from 1999 to 2006 (APC 3.50%), a subsequent decline from 2006 to 2013 (APC −3.32%), and a stable to slightly increasing trend from 2013 to 2023 (APC 0.92%).

#### 3.4.2 State‐Stratified

State‐level counts and age‐adjusted rates of deaths with both Alzheimer's disease and cancer recorded on death certificates varied across U.S. jurisdictions during 1999–2023. Cumulative death counts over the full study period were highest in California (*n* = 19,608), Texas (*n* = 9883), Ohio (*n* = 6862), Florida (*n* = 6605), and Pennsylvania (*n* = 6483). Vermont (*n* = 554), Hawaii (*n* = 655), Utah (*n* = 655), and New Mexico (*n* = 667) each recorded fewer than 1000 deaths. Full‐period AAPC point estimates were negative in Maryland (−2.70%), New Hampshire (−3.45%), Illinois (−2.33%), and Massachusetts (−2.06%), and positive in Oregon (2.42%), Oklahoma (3.19%), Nebraska (1.13%), Wisconsin (0.33%), and Minnesota (0.96%). The corresponding 95% confidence intervals are presented in Table S1; the direction of a point estimate alone does not establish a statistically significant trend.

These AAPCs summarize average annual changes over 1999–2023 and do not characterize changes specifically in the final years of observation. No separate recent‐period state‐specific analysis was performed; therefore, the full‐period AAPCs, endpoint rates, and cumulative death counts cannot establish whether mortality increases accelerated as 2023 approached or whether earlier declines continued during recent years. State‐level AAMRs and AAPCs were unavailable for several jurisdictions, including Alaska, the District of Columbia, Montana, Nevada, South Dakota, Delaware, and Wyoming, because the available data were insufficient for estimation. These unavailable estimates limit the completeness of state‐level comparisons and should not be interpreted as evidence of deficiencies in mortality reporting.

### 3.5 Race‐Stratified Analyses

Race‐ and ethnicity‐specific rates of deaths with both AD and cancer recorded on death certificates were examined during 1999–2023 (Figure 5). Cumulative deaths were highest among non‐Hispanic White individuals (*n* = 122,214), followed by non‐Hispanic Black (*n* = 11,711), Hispanic (*n* = 6225), and non‐Hispanic Other populations (*n* = 2902). The AAMR for non‐Hispanic Black individuals declined from 16.35 to 10.56 per 100,000, corresponding to a statistically significant AAPC of −1.69% (95% CI −3.09 to −0.26). Non‐Hispanic White individuals experienced a more modest, non‐significant reduction in AAMR from 14.21 to 12.85 per 100,000 (AAPC −0.56%, 95% CI −1.25 to 0.13). In contrast, the AAMR increased significantly among Hispanic individuals (AAPC 2.58%, 95% CI 0.93 to 4.25). Among non‐Hispanic Other individuals, the AAPC was positive (0.73%, 95% CI −1.35 to 2.86), but the confidence interval included zero, indicating no statistically significant long‐term change.

**FIGURE 5:**
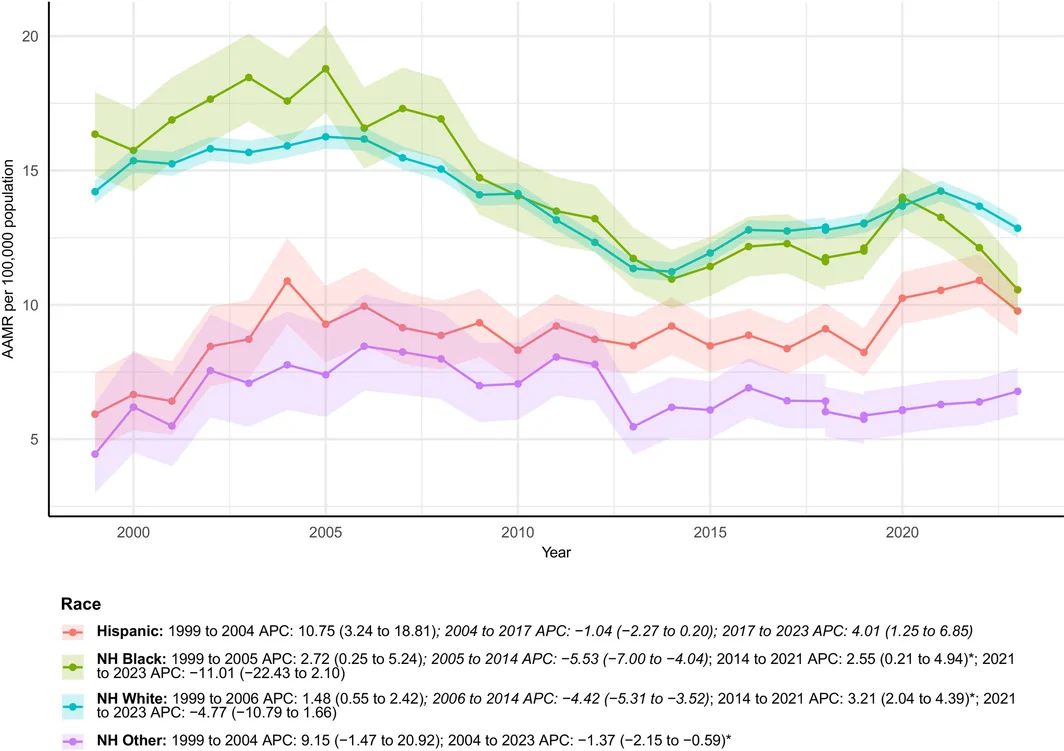
Age‐adjusted mortality rates (AAMRs) for deaths with both Alzheimer's disease and cancer recorded on death certificates among U.S. adults aged ≥ 65 years, stratified by race and ethnicity, 1999–2023. Rates are expressed per 100,000 persons in the corresponding racial and ethnic general‐population group aged ≥ 65 years. Solid lines represent estimated mortality trends, and shaded areas indicate the corresponding 95% confidence intervals. Abbreviations: AAMR, age‐adjusted mortality rate; AAPC, average annual percent change; NH, non‐Hispanic.

Joinpoint analyses revealed distinct temporal trajectories across racial and ethnic groups. Among Hispanic individuals, mortality increased sharply from 1999 to 2004 (APC 10.75%, 95% CI 3.24 to 18.81), showed no statistically significant trend during 2004–2017 (APC −1.04%, 95% CI −2.27 to 0.20), and then rose moderately from 2017 to 2023 (APC 4.01%, 95% CI 1.25 to 6.85). For non‐Hispanic Black individuals, an initial increase from 1999 to 2005 (APC 2.72%) was followed by a pronounced decline from 2005 to 2014 (APC −5.53%) and a subsequent significant increase from 2014 to 2021 (APC 2.55%). Non‐Hispanic White individuals exhibited an initial increase (1999–2006, APC 1.48%), a marked decline (2006–2014, APC −4.42%), and a significant subsequent rise (2014–2021, APC 3.21%). The non‐Hispanic Other population showed a rapid early increase (1999–2004, APC 9.15%) followed by a long‐term declining trend from 2004 to 2023 (APC −1.37%). Over the full study period, AAMRs increased significantly among Hispanic populations and declined significantly among non‐Hispanic Black populations. Full‐period trends among non‐Hispanic White and non‐Hispanic Other populations were not statistically significant. These subgroup‐specific findings do not establish statistically significant differences between groups.

### 3.6 Urbanization‐ Stratified Analyses

Urbanization‐specific trends in population‐level rates of deaths with both Alzheimer's disease and cancer recorded on death certificates were assessed for 1999–2020 (Figure 6). Cumulative death counts were 98,154 in metropolitan areas and 25,468 in nonmetropolitan areas. In metropolitan areas, the AAMR decreased from 13.55 to 12.70 per 100,000 (AAPC −0.48%, 95% CI −1.00 to 0.04). In nonmetropolitan areas, the AAMR decreased from 15.52 to 14.77 per 100,000 (AAPC −0.37%, 95% CI −1.06 to 0.32). Neither full‐period trend was statistically significant.

**FIGURE 6:**
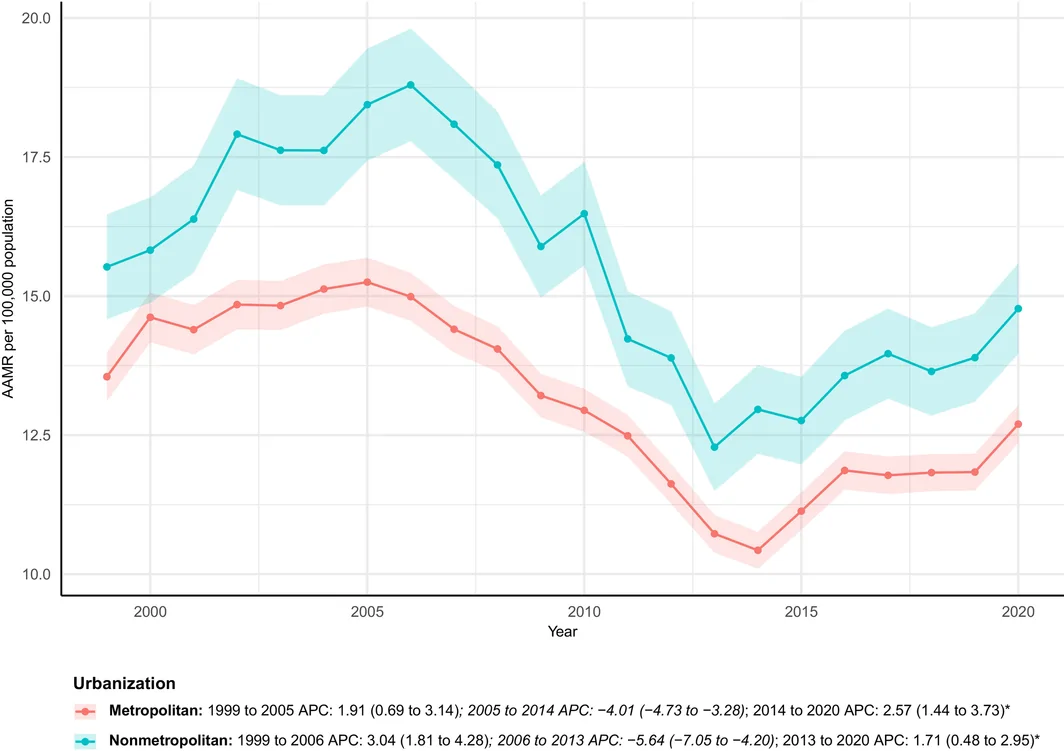
Age‐adjusted mortality rates (AAMRs) for deaths with both Alzheimer's disease and cancer recorded on death certificates among U.S. adults aged ≥ 65 years, stratified by urbanization, 1999–2020. Metropolitan and nonmetropolitan counties were classified using the 2013 NCHS Urban–Rural Classification Scheme. Rates are expressed per 100,000 persons in the corresponding general population aged ≥ 65 years. Urbanization analyses were restricted to the 1999–2020 data extracted for this study. Solid lines represent estimated mortality trends, and shaded areas indicate the corresponding 95% confidence intervals. Abbreviations: AAMR, age‐adjusted mortality rate; AAPC, average annual percent change; NCHS, National Centre for Health Statistics.

Within metropolitan areas, Joinpoint regression identified an increase during 1999–2005 (APC 1.91%, 95% CI 0.69 to 3.14), a decline during 2005–2014 (APC −4.01%, 95% CI −4.73 to −3.28), and a subsequent increase during 2014–2020 (APC 2.57%, 95% CI 1.05 to 4.12). Within nonmetropolitan areas, rates increased during 1999–2006 (APC 3.04%, 95% CI 0.99 to 5.13), declined during 2006–2013 (APC −5.64%, 95% CI −7.24 to −4.01), and increased during 2013–2020 (APC 1.71%, 95% CI 0.36 to 3.08). These estimates describe temporal changes within each subgroup. No formal between‐group comparison was performed; therefore, differences in point estimates or estimated joinpoint years should not be interpreted as evidence of statistically significant differences in trends by urbanization status.

### 3.7 Sensitivity Analysis Excluding the COVID‐19 Pandemic Period

To assess the potential influence of the COVID‐19 pandemic on long‐term mortality trends, we performed a sensitivity analysis by restricting the study period to 1999–2019. The results were generally consistent with those of the primary analysis covering 1999–2023 (Table S5). The overall mortality trend remained decreasing, with an AAPC of −0.78% (95% CI: −1.31 to −0.24) during 1999–2019, compared with −0.73% (95% CI: −1.39 to −0.06) in the full study period. The increasing trend observed among Hispanic individuals persisted after excluding the pandemic period (AAPC: 1.82%, 95% CI: 0.64 to 3.01), although the magnitude was slightly attenuated compared with the full‐period analysis (AAPC: 2.58%, 95% CI: 0.93 to 4.25). The overall decline and the increase among Hispanic populations were also observed during 1999–2019. This comparison of overlapping periods does not quantify the effect of the COVID‐19 pandemic.

In the overlapping‐period sensitivity analysis, Hispanic AAMRs were identical across the two data sources in 2018–2020 (Table S6). Absolute differences in AAMRs ranged from 0.02 to 0.11 per 100,000 for NH White individuals and from 0.11 to 0.14 per 100,000 for NH Black individuals, corresponding to relative differences below 1.3%. Differences for the heterogeneous NH Other category were larger in 2018 (−0.39 per 100,000; −6.11%) but were smaller in 2019 and 2020 (+2.40% and + 0.41%, respectively). These comparisons showed small differences in annual AAMRs for Hispanic, NH White, and NH Black populations during 2018–2020, with greater variation for NH Other. However, this overlap comparison does not establish that full‐period APCs or AAPCs are unaffected by the transition or assess the effects of population‐denominator changes after 2020.

## 4 Discussion

In this national analysis of death certificates from 1999 to 2023, the population‐level age‐adjusted rate of deaths with both AD and cancer recorded declined modestly, while the annual number of such deaths increased. These findings describe changes in recorded mortality at the population level and should not be interpreted as evidence of improved survival among individuals living with both conditions. The observed patterns may reflect changes in disease occurrence, diagnosis, death‐certificate reporting, and population composition; the present data cannot distinguish their respective contributions.

The highest age‐specific mortality rates were observed among adults aged 85 years and above. Previous literature has discussed frailty, multimorbidity, and reduced physiological reserve as potential contributors to complex health needs in later life (Cho et al. 2025). Biological pathways involving inflammation, DNA repair, and mitochondrial function have also been investigated in relation to neurodegeneration and cancer (Chen et al. 2025; Fu et al. 2025). These findings provide possible context for the observed age‐related patterns, but the present analysis did not measure frailty, functional status, or biological mechanisms and cannot determine their contributions. Differences in cancer type, stage, and treatment may also be relevant; however, their relationships with the observed mortality patterns were not evaluated. The higher rates in the oldest age group should therefore be interpreted as descriptive population‐level findings rather than evidence of specific biological or clinical mechanisms.

Variation in death‐certificate mortality rates was also observed by sex and race/ethnicity. Previous studies have examined sex differences in cancer outcomes and dementia‐related clinical characteristics, which may provide context for these patterns (Caba et al. 2021; Lachner et al. 2022; Snider et al. 2023). However, the present data cannot determine whether differences in cancer type, comorbidity, diagnosis, treatment, or biological characteristics explain the observed sex‐specific rates. Non‐Hispanic Black populations had the highest AAMRs early in the study period, followed by a decline and numerical convergence with non‐Hispanic White populations in later years. Differences in healthcare access, socioeconomic circumstances, and diagnostic or certification practices are possible explanations requiring further investigation. The present analysis did not measure these factors or evaluate whether changes in healthcare coverage or delivery contributed to the observed pattern (McInerney et al. 2020; Ozten et al. 2019; Vegeto et al. 2020). Hispanic populations showed a statistically significant increase in AAMRs, whereas the full‐period increase among non‐Hispanic Other populations was not statistically significant. These findings should not be interpreted as direct evidence of changes in individual vulnerability or the effectiveness of care.

Geographic analyses showed variation in population‐level death‐certificate mortality rates. The Western region had a positive full‐period AAPC, although its confidence interval included zero. Nonmetropolitan areas had numerically higher AAMRs than metropolitan areas at the beginning and end of the 1999–2020 urbanization analysis, while neither area showed a statistically significant full‐period trend. No formal between‐group comparison was performed, and these findings do not establish differences in temporal trends between regions or urbanization groups. Previous literature has discussed geographic variation in healthcare access and specialist availability, while environmental exposures have also been investigated in relation to cancer and cognitive outcomes (Bhatia et al. 2022; Khan et al. 2024; Laguna et al. 2024; Mohammadzadeh et al. 2024; Monnat 2025; Morris et al. 2023; Rahman et al. 2021). These factors offer possible context but were not measured in this study. We therefore cannot determine whether healthcare access, cancer treatment, socioeconomic conditions, environmental exposures, or differences in cancer composition contributed to the observed geographic patterns. Differences in diagnosis and death‐certificate reporting may also affect the recorded rates. Studies linking mortality information with clinical and contextual measures are needed to evaluate these possibilities.

The observed population‐level patterns identify areas for further assessment of the needs of older adults with cognitive impairment and cancer. Coordinated geriatric, oncology, and neurological care may warrant consideration, with attention to cognitive and functional assessment, treatment burden, medication management, caregiver support, and goals of care. Nurses may contribute to this assessment and facilitate communication across patients, caregivers, and clinical teams. Demographic and geographic variation also supports further investigation of potential barriers to appropriate care. However, the present findings do not establish the presence or causes of specific service gaps and cannot determine whether particular care models, screening strategies, or resource‐allocation policies would improve outcomes. Such approaches require evaluation using individual‐level clinical and healthcare information.

This study has several limitations. As a retrospective analysis of death certificate data from CDC WONDER, it is subject to potential misclassification bias from ICD‐10 coding practices and incomplete reporting of Alzheimer's disease as an underlying or contributing cause of death. Previous studies have demonstrated that Alzheimer's disease mortality is substantially underreported on death certificates, particularly among older adults with multiple comorbidities, as immediate causes of death may be preferentially recorded (Sato et al. 2025). The absence of detailed clinical data—including cognitive status, disease severity, comorbidities, and treatment information—prevents assessment of disease progression and the potential impact of clinical interventions. In addition, death certificates do not identify a cohort of individuals living with both AD and cancer, and population denominators specific to this comorbid group were unavailable. Consequently, this study cannot estimate all‐cause mortality risk, case fatality, or survival among individuals living with both AD and cancer. Co‐recording of the two conditions also does not establish their temporal relationship or a causal interaction. Incomplete recording and changes in certification practices may affect both the level and temporal pattern of the reported rates. Furthermore, socioeconomic conditions, healthcare access, cancer treatment, environmental exposures, and biological mechanisms were not directly measured. Explanations involving these factors are therefore hypotheses informed by previous literature rather than findings established by the present analysis. We could not determine whether, or to what extent, they contributed to the observed demographic and geographic patterns. The transition from bridged‐race to single‐race classification and changes in population‐denominator methodology may affect the comparability of race‐specific estimates over time. Although the 2018–2020 overlap analysis showed small differences for Hispanic, NH White, and NH Black populations, it did not evaluate the effects of denominator changes after 2020. The NH Other category is not strictly equivalent across classification systems, and its temporal trends require particular caution. Finally, multiple state‐level and subgroup analyses were conducted without adjustment for multiple comparisons across analyses, increasing the possibility of chance findings. The reported *p* values and confidence intervals are nominal, and these findings should be interpreted as exploratory rather than confirmatory. Being an ecological study, these findings cannot establish causality, underscoring the need for prospective studies incorporating comprehensive clinical and social determinants data.

## 5 Conclusion

From 1999 to 2023, the population‐level age‐adjusted rate of deaths with both AD and cancer recorded on death certificates declined modestly among U.S. adults aged ≥ 65 years, despite an increase in annual death counts. Patterns varied across demographic and geographic groups. These findings support continued mortality surveillance and further investigation using linked clinical data to assess outcomes among individuals living with both conditions.

## 6 Implications for Nursing Practice

The population‐level patterns identified in this study have several implications for nursing practice in the care of older adults with complex health needs. Geriatric and oncology nurses are often positioned to identify overlapping cognitive, functional, symptom‐management, and cancer‐care needs among older adults. Coordinated nursing assessment may therefore include attention to cognitive status, functional ability, treatment burden, medication management, caregiver support, and goals of care when Alzheimer's disease and cancer coexist.

The observed demographic and geographic variation also supports attention to continuity and coordination across oncology, geriatric, primary‐care, and supportive‐care services. Nurses may contribute to communication among multidisciplinary teams, patients, and caregivers and help identify barriers to follow‐up and supportive services. However, because this study was descriptive and based on aggregate death‐certificate data, it did not evaluate specific nursing interventions or models of care. These findings should therefore be viewed as informing areas for clinical attention and future research rather than demonstrating the effectiveness of any particular nursing strategy.

## Author Contributions

**Xiangxiang Zhang:** conceptualization, investigation, writing – original draft. **Yu‐Jun Xiong:** methodology, validation, supervision. **Tian Lv:** investigation, formal analysis, software. **Yiqiao Chen:** writing – review and editing, project administration, software, supervision, data curation.

## Funding

This study was supported by Zhejiang Provincial Health Industry Science and Technology Program (2025HY1587).

## Disclosure

*Statistical Statement:* There is a statistician on the author team: Yu‐Jun Xiong.

The authors affirm that the methods used in the data analyses are appropriate for the study design and data, and that the statistical findings have been correctly implemented and interpreted.

The authors take full responsibility for the appropriateness of the statistical analyses and their correct implementation and interpretation.

## Ethics Statement

This study used publicly available, de‐identified, aggregate mortality data obtained from CDC WONDER and involved no direct interaction with human participants and no access to identifiable private information. Therefore, institutional ethics committee approval and informed consent were not required for this analysis. No formal institutional exemption determination was obtained.

## Conflicts of Interest

The authors declare no conflicts of interest.

## Supporting information


**Table S1:** State‐specific mortality and age‐adjusted mortality rates for Alzheimer's disease with concurrent cancers in 1999 and 2023.
**Table S2:** Age‐adjusted mortality rates and standard errors by characteristics across years.
**Table S3:** Characteristics with APC, 95% CI, and Time Range.
**Table S4:** Characteristics with AAPC, 95% CI, and Time Range.
**Table S5:** Sensitivity analysis comparing AAPCs for the full study period (1999–2023) and the restricted pre‐pandemic period (1999–2019).
**Table S6:** Comparison of race‐ and ethnicity‐specific age‐adjusted mortality rates from the bridged‐race and single‐race CDC WONDER datasets during the overlapping period 2018 to 2020.

## Data Availability

The mortality data used in this study are publicly available through CDC WONDER (https://wonder.cdc.gov/). Data for 1999–2020 were obtained from the Multiple Cause of Death, 1999–2020 dataset using bridged‐race categories, and data for 2021–2023 were obtained from the Multiple Cause of Death, 2018–2023 dataset using single‐race categories. Data were extracted on June 20, 2026. The principal extraction criteria were U.S. residents aged ≥ 65 years with Alzheimer's disease (ICD‐10 G30) and malignant neoplasms (ICD‐10 C00–C97) both recorded on the death certificate using the AND operator, with underlying cause retained as All Causes. The subgroup definitions and statistical procedures required to reproduce the analyses are described in the Methods and supporting information.
