# Supplementary material for: Trends in Population‐Level Mortality Rates for Deaths With Both Alzheimer's Disease and Cancer Recorded on Death Certificates Among U.S. Adults Aged ≥ 65 Years, 1999–2023
Source: Nurs Open. 2026 Oct 6;13(10):e70897. doi: 10.1002/nop2.70897 (PMC13639708; doi:10.1002/nop2.70897)
Supplement: Supplementary file 1 [file NOP2-13-e70897-s001.docx]

Supplementary table 1. State-specific Mortality and Age-Adjusted Mortality Rates for Alzheimer’s disease with concurrent cancers in 1999 and 2023.

|  | States | Deaths | | | AAMR | | |
| --- | --- | --- | --- | --- | --- | --- | --- |
|  |  | 1999 | 2023 | Percent Change | 1999 | 2023 | AAPC (95% CI) |
|  | Alabama | 72 | 69 | -4.17 | 13.21 (10.34 to 16.64) | 8.60 (6.68 to 10.90) | -1.47 (-3.78 to 0.88) |
|  | Alaska | NA | NA | NA | NA | NA | NA |
|  | Arizona | 51 | 119 | 133.33 | 8.45 (6.29 to 11.12) | 9.27 (7.60 to 10.94) | 0.04 (-1.00 to 1.10 |
|  | Arkansas | 33 | 57 | 72.73 | 8.93 (6.15 to 12.54) | 11.63 (8.81 to 15.07) | 2.73 (-0.24 to 5.79) |
|  | California | 478 | 778 | 62.76 | 13.80 (12.56 to 15.04) | 13.58 (12.63 to 14.54) | -0.03 (-0.76 to 0.71) |
|  | Colorado | 61 | 163 | 167.21 | 15.63 (11.95 to 20.07) | 21.75 (18.39 to 25.10) | 1.88 (-3.70 to 7.78) |
|  | Connecticut | 62 | 38 | -38.71 | 12.45 (9.54 to 15.96) | 5.87 (4.16 to 8.06) | -2.50 (-5.38 to 0.47) |
|  | Delaware | 15 | 24 | 60 | NA (9.20 to 27.10) | 13.53 (8.67 to 20.12) | NA |
|  | District of Columbia | NA | NA | NA | NA | NA | NA |
|  | Florida | 251 | 319 | 27.09 | 9.19 (8.05 to 10.33) | 6.67 (5.94 to 7.40) | -1.18 (-3.41 to 1.11) |
|  | Georgia | 99 | 182 | 83.84 | 13.55 (11.01 to 16.50) | 13.13 (11.20 to 15.05) | -1.02 (-2.41 to 0.38) |
|  | Hawaii | 22 | 38 | 72.73 | 15.05 (9.43 to 22.79) | 12.14 (8.59 to 16.66) | NA |
|  | Idaho | 33 | 51 | 54.55 | 22.99 (15.83 to 32.29) | 18.44 (13.68 to 24.30) | NA |
|  | Illinois | 212 | 158 | -25.47 | 13.95 (12.07 to 15.82) | 8.02 (6.77 to 9.28) | -2.33 (-3.69 to -0.94)* |
|  | Indiana | 107 | 108 | 0.93 | 14.44 (11.71 to 17.18) | 10.57 (8.57 to 12.57) | -2.38 (-4.91 to 0.21) |
|  | Iowa | 81 | 92 | 13.58 | 16.51 (13.09 to 20.55) | 16.52 (13.30 to 20.28) | -0.32 (-1.29 to 0.65) |
|  | Kansas | 54 | 46 | -14.81 | 13.88 (10.43 to 18.11) | 9.92 (7.26 to 13.23) | -1.64 (-5.27 to 2.13) |
|  | Kentucky | 82 | 102 | 24.39 | 17.24 (13.71 to 21.40) | 15.53 (12.50 to 18.55) | -0.90 (-1.62 to -0.18)* |
|  | Louisiana | 51 | 60 | 17.65 | 10.61 (7.90 to 13.96) | 9.12 (6.94 to 11.77) | -0.30 (-3.13 to 2.61) |
|  | Maine | 39 | 33 | -15.38 | 21.25 (15.11 to 29.05) | 12.07 (8.31 to 16.95) | NA |
|  | Maryland | 116 | 89 | -23.28 | 20.63 (16.87 to 24.39) | 9.35 (7.51 to 11.50) | -2.70 (-4.26 to -1.11)* |
|  | Massachusetts | 135 | 123 | -8.89 | 14.94 (12.42 to 17.46) | 10.53 (8.66 to 12.39) | -2.06 (-3.51 to -0.58)* |
|  | Michigan | 173 | 192 | 10.98 | 14.72 (12.52 to 16.91) | 11.99 (10.29 to 13.69) | -0.85 (-1.91 to 0.21) |
|  | Minnesota | 104 | 210 | 101.92 | 15.95 (12.88 to 19.02) | 22.81 (19.71 to 25.90) | 0.96 (-1.41 to 3.39) |
|  | Mississippi | 53 | 94 | 77.36 | 15.88 (11.90 to 20.78) | 21.68 (17.50 to 26.56) | 0.64 (-0.37 to 1.66) |
| Missouri | | 128 | 103 | -19.53 | 16.59 (13.72 to 19.47) | 10.30 (8.31 to 12.30) | -1.52 (-3.37 to 0.37) |
| Montana | | 14 | 17 | 21.43 | NA (6.29 to 19.32) | NA (5.32 to 14.62) | NA |
| Nebraska | | 42 | 62 | 47.62 | 16.25 (11.71 to 21.97) | 20.35 (15.60 to 26.08) | 1.13 (0.02 to 2.26)* |
| Nevada | | 13 | 37 | 184.62 | NA (4.60 to 14.77) | 8.84 (6.19 to 12.24) | NA |
| New Hampshire | | 29 | 28 | -3.45 | 20.05 (13.43 to 28.79) | 11.20 (7.44 to 16.19) | -3.45 (-4.47 to -2.42)* |
| New Jersey | | 109 | 104 | -4.59 | 9.93 (8.07 to 11.80) | 6.84 (5.53 to 8.16) | -1.60 (-2.73 to -0.45)* |
| New Mexico | | 25 | 28 | 12 | 13.12 (8.49 to 19.37) | 8.20 (5.45 to 11.85) | NA |
| New York | | 250 | 247 | -1.2 | 10.25 (8.98 to 11.52) | 7.22 (6.32 to 8.12) | -1.25 (-2.30 to -0.19)* |
| North Carolina | | 144 | 227 | 57.64 | 16.08 (13.45 to 18.71) | 14.25 (12.38 to 16.12) | -0.74 (-4.00 to 2.64) |
| North Dakota | | 19 | 28 | 47.37 | NA (10.45 to 27.11) | 22.11 (14.57 to 32.17) | NA |
| Ohio | | 295 | 214 | -27.46 | 20.19 (17.88 to 22.49) | 11.34 (9.82 to 12.86) | -1.98 (-3.20 to -0.74)* |
| Oklahoma | | 49 | 118 | 140.82 | 10.75 (7.95 to 14.21) | 20.23 (16.57 to 23.90) | 3.19 (-0.40 to 6.92 |
| Oregon | | 64 | 196 | 206.25 | 14.12 (10.88 to 18.04) | 28.81 (24.75 to 32.87) | 2.42 (1.33 to 3.52)* |
| Pennsylvania | | 263 | 225 | -14.45 | 13.60 (11.96 to 15.24) | 9.53 (8.28 to 10.77) | -1.39 (-2.35 to -0.42)* |
| Rhode Island | | 25 | 37 | 48 | 15.23 (9.86 to 22.49) | 19.05 (13.41 to 26.26) | NA |
| South Carolina | | 74 | 141 | 90.54 | 17.20 (13.51 to 21.60) | 17.18 (14.31 to 20.04) | -0.60 (-1.48 to 0.29) |
| South Dakota | | 13 | 34 | 161.54 | NA (5.97 to 19.17) | 23.33 (16.16 to 32.61) | NA |
| Tennessee | | 112 | 121 | 8.04 | 16.77 (13.67 to 19.88) | 11.67 (9.57 to 13.77) | -1.05 (-2.68 to 0.61) |
| Texas | | 301 | 512 | 70.1 | 15.55 (13.79 to 17.31) | 14.87 (13.57 to 16.16) | -0.19 (-1.79 to 1.44) |
| Utah | | 10 | 37 | 270 | NA (2.68 to 10.28) | 11.23 (7.87 to 15.55) | NA |
| Vermont | | 15 | 22 | 46.67 | NA (10.74 to 31.66) | 18.78 (11.77 to 28.43) | NA |
| Virginia | | 93 | 145 | 55.91 | 12.64 (10.20 to 15.48) | 11.14 (9.32 to 12.96) | -1.41 (-5.78 to 3.16) |
| Washington | | 133 | 225 | 69.17 | 19.89 (16.51 to 23.27) | 20.26 (17.60 to 22.92) | 0.55 (-2.13 to 3.30) |
| West Virginia | | 35 | 26 | -25.71 | 13.21 (9.20 to 18.37) | 8.28 (5.41 to 12.13) | -2.74 (-4.52 to -0.92)* |
| Wisconsin | | 102 | 140 | 37.25 | 13.71 (11.04 to 16.37) | 14.58 (12.15 to 17.00) | 0.33 (-1.00 to 1.67) |
| Wyoming | | 11 |  | NA | NA (10.15 to 36.37) | NA (NA to NA) | NA |

* indicates statistically significant AAPC.

#Age-group AAMR and APC are calculated using crude rates.

Abbreviations: Age adjusted mortality rate (AAMR), confidence interval (CI), average annual percentage change (AAPC), non-Hispanic (NH).

Supplementary table 2. Age-Adjusted Mortality Rates and Standard Errors by Characteristics Across Years.

| Characteristics | Year | AAMR | SE |
| --- | --- | --- | --- |
| Census Region_Midwest | 1999 | 15.8394615 | 0.43441255 |
| Census Region_Midwest | 2000 | 16.54550822 | 0.441682373 |
| Census Region_Midwest | 2001 | 15.84906614 | 0.428962016 |
| Census Region_Midwest | 2002 | 16.73201149 | 0.439198059 |
| Census Region_Midwest | 2003 | 16.28704672 | 0.42979727 |
| Census Region_Midwest | 2004 | 17.0849751 | 0.438779174 |
| Census Region_Midwest | 2005 | 17.15055596 | 0.437059517 |
| Census Region_Midwest | 2006 | 18.01611249 | 0.444239654 |
| Census Region_Midwest | 2007 | 16.23023686 | 0.41913302 |
| Census Region_Midwest | 2008 | 15.66948907 | 0.409437504 |
| Census Region_Midwest | 2009 | 14.66789543 | 0.392167924 |
| Census Region_Midwest | 2010 | 13.97695124 | 0.381796631 |
| Census Region_Midwest | 2011 | 13.03725753 | 0.36445096 |
| Census Region_Midwest | 2012 | 12.8867792 | 0.363090321 |
| Census Region_Midwest | 2013 | 11.54770447 | 0.340567238 |
| Census Region_Midwest | 2014 | 11.51511796 | 0.336626662 |
| Census Region_Midwest | 2015 | 11.59057612 | 0.336098709 |
| Census Region_Midwest | 2016 | 12.82937818 | 0.35178359 |
| Census Region_Midwest | 2017 | 12.9785692 | 0.350265359 |
| Census Region_Midwest | 2018 | 13.23298545 | 0.349552652 |
| Census Region_Midwest | 2019 | 13.27407029 | 0.348114396 |
| Census Region_Midwest | 2020 | 13.88411643 | 0.35443707 |
| Census Region_Midwest | 2021 | 14.27012022 | 0.37193556 |
| Census Region_Midwest | 2022 | 13.29323375 | 0.345834495 |
| Census Region_Midwest | 2023 | 12.64187593 | 0.340319116 |
| Census Region_Northeast | 1999 | 12.44914495 | 0.408901009 |
| Census Region_Northeast | 2000 | 13.00316904 | 0.415263483 |
| Census Region_Northeast | 2001 | 13.51115572 | 0.419025307 |
| Census Region_Northeast | 2002 | 13.04031597 | 0.409733709 |
| Census Region_Northeast | 2003 | 13.49750583 | 0.413792897 |
| Census Region_Northeast | 2004 | 12.93143764 | 0.403171008 |
| Census Region_Northeast | 2005 | 12.40283488 | 0.392366533 |
| Census Region_Northeast | 2006 | 11.46130854 | 0.373924458 |
| Census Region_Northeast | 2007 | 11.84193393 | 0.377094331 |
| Census Region_Northeast | 2008 | 10.71262917 | 0.3577751 |
| Census Region_Northeast | 2009 | 10.6197822 | 0.353753384 |
| Census Region_Northeast | 2010 | 10.84599134 | 0.355120158 |
| Census Region_Northeast | 2011 | 10.64371722 | 0.349703765 |
| Census Region_Northeast | 2012 | 9.059723548 | 0.320888747 |
| Census Region_Northeast | 2013 | 8.666888738 | 0.311322341 |
| Census Region_Northeast | 2014 | 8.454283654 | 0.307754129 |
| Census Region_Northeast | 2015 | 8.615865089 | 0.307516004 |
| Census Region_Northeast | 2016 | 8.808926728 | 0.308788423 |
| Census Region_Northeast | 2017 | 8.552904682 | 0.298726343 |
| Census Region_Northeast | 2018 | 8.647345293 | 0.301331023 |
| Census Region_Northeast | 2019 | 8.789263789 | 0.301285487 |
| Census Region_Northeast | 2020 | 9.187657988 | 0.305383765 |
| Census Region_Northeast | 2021 | 9.128969662 | 0.311612004 |
| Census Region_Northeast | 2022 | 8.801761539 | 0.296631214 |
| Census Region_Northeast | 2023 | 8.639193287 | 0.295543008 |
| Census Region_South | 1999 | 13.46429682 | 0.338012186 |
| Census Region_South | 2000 | 14.40131209 | 0.347484694 |
| Census Region_South | 2001 | 14.39022114 | 0.345424827 |
| Census Region_South | 2002 | 15.22264233 | 0.35334231 |
| Census Region_South | 2003 | 15.16622813 | 0.350325885 |
| Census Region_South | 2004 | 15.16844075 | 0.347781058 |
| Census Region_South | 2005 | 15.52319116 | 0.347486684 |
| Census Region_South | 2006 | 15.18021218 | 0.339828571 |
| Census Region_South | 2007 | 14.61864291 | 0.329780022 |
| Census Region_South | 2008 | 14.48416226 | 0.32464313 |
| Census Region_South | 2009 | 13.31565463 | 0.307838975 |
| Census Region_South | 2010 | 13.20621531 | 0.304464788 |
| Census Region_South | 2011 | 11.46580745 | 0.278461161 |
| Census Region_South | 2012 | 11.09490619 | 0.270499068 |
| Census Region_South | 2013 | 10.06687658 | 0.253814736 |
| Census Region_South | 2014 | 9.723624941 | 0.246518029 |
| Census Region_South | 2015 | 10.92817319 | 0.257011587 |
| Census Region_South | 2016 | 11.20240554 | 0.256777748 |
| Census Region_South | 2017 | 11.71695059 | 0.259454689 |
| Census Region_South | 2018 | 11.21090387 | 0.249085593 |
| Census Region_South | 2019 | 11.58269677 | 0.249491602 |
| Census Region_South | 2020 | 12.34060079 | 0.254444595 |
| Census Region_South | 2021 | 13.04698167 | 0.270622985 |
| Census Region_South | 2022 | 12.52649724 | 0.254197125 |
| Census Region_South | 2023 | 11.73955247 | 0.246214337 |
| Census Region_West | 1999 | 13.94063797 | 0.460378284 |
| Census Region_West | 2000 | 15.79715085 | 0.484258276 |
| Census Region_West | 2001 | 15.52729791 | 0.474142842 |
| Census Region_West | 2002 | 16.93012087 | 0.490050557 |
| Census Region_West | 2003 | 16.70436538 | 0.480445092 |
| Census Region_West | 2004 | 17.36266155 | 0.485118095 |
| Census Region_West | 2005 | 18.59207365 | 0.494468117 |
| Census Region_West | 2006 | 18.28246901 | 0.483203901 |
| Census Region_West | 2007 | 17.84121533 | 0.471468503 |
| Census Region_West | 2008 | 17.67998021 | 0.463686648 |
| Census Region_West | 2009 | 16.14567705 | 0.436824991 |
| Census Region_West | 2010 | 16.36266155 | 0.43546418 |
| Census Region_West | 2011 | 16.73440862 | 0.430631249 |
| Census Region_West | 2012 | 15.38094201 | 0.40815172 |
| Census Region_West | 2013 | 14.1093266 | 0.385817748 |
| Census Region_West | 2014 | 14.36134923 | 0.38482404 |
| Census Region_West | 2015 | 14.55301973 | 0.380769488 |
| Census Region_West | 2016 | 16.07894406 | 0.394844674 |
| Census Region_West | 2017 | 15.11654113 | 0.378961169 |
| Census Region_West | 2018 | 15.61625631 | 0.379494898 |
| Census Region_West | 2019 | 15.0865014 | 0.367730687 |
| Census Region_West | 2020 | 16.72507664 | 0.383059744 |
| Census Region_West | 2021 | 16.67865923 | 0.396244125 |
| Census Region_West | 2022 | 16.3454659 | 0.375026609 |
| Census Region_West | 2023 | 14.69989079 | 0.356750907 |
| Race_Hispanic | 1999 | 5.931442087 | 0.690642967 |
| Race_Hispanic | 2000 | 6.664850093 | 0.710276244 |
| Race_Hispanic | 2001 | 6.417219042 | 0.675788665 |
| Race_Hispanic | 2002 | 8.455628955 | 0.756311965 |
| Race_Hispanic | 2003 | 8.720801985 | 0.74116604 |
| Race_Hispanic | 2004 | 10.8865767 | 0.81742946 |
| Race_Hispanic | 2005 | 9.280645965 | 0.726603493 |
| Race_Hispanic | 2006 | 9.952773026 | 0.730340563 |
| Race_Hispanic | 2007 | 9.148893101 | 0.682566173 |
| Race_Hispanic | 2008 | 8.864341952 | 0.651749002 |
| Race_Hispanic | 2009 | 9.334455388 | 0.647280783 |
| Race_Hispanic | 2010 | 8.311424173 | 0.597519295 |
| Race_Hispanic | 2011 | 9.214139794 | 0.602511785 |
| Race_Hispanic | 2012 | 8.719423014 | 0.565596985 |
| Race_Hispanic | 2013 | 8.483658568 | 0.542673323 |
| Race_Hispanic | 2014 | 9.209533551 | 0.544637027 |
| Race_Hispanic | 2015 | 8.474446082 | 0.50631057 |
| Race_Hispanic | 2016 | 8.87199447 | 0.506411335 |
| Race_Hispanic | 2017 | 8.371672468 | 0.476301918 |
| Race_Hispanic | 2018 | 9.109640885 | 0.486027521 |
| Race_Hispanic | 2019 | 8.231194446 | 0.452699557 |
| Race_Hispanic | 2020 | 10.24966876 | 0.493528996 |
| Race_Hispanic | 2021 | 10.54200408 | 0.506349895 |
| Race_Hispanic | 2022 | 10.90963223 | 0.49848503 |
| Race_Hispanic | 2023 | 9.771663816 | 0.469298311 |
| Race_NH Black | 1999 | 16.3510026 | 0.797604 |
| Race_NH Black | 2000 | 15.74800215 | 0.779224167 |
| Race_NH Black | 2001 | 16.88141367 | 0.805921161 |
| Race_NH Black | 2002 | 17.65609968 | 0.821407981 |
| Race_NH Black | 2003 | 18.45992161 | 0.83632442 |
| Race_NH Black | 2004 | 17.58752189 | 0.812566892 |
| Race_NH Black | 2005 | 18.78705607 | 0.828630779 |
| Race_NH Black | 2006 | 16.57709576 | 0.772513973 |
| Race_NH Black | 2007 | 17.3052907 | 0.779129468 |
| Race_NH Black | 2008 | 16.9199059 | 0.759102829 |
| Race_NH Black | 2009 | 14.73262697 | 0.701134485 |
| Race_NH Black | 2010 | 14.0602394 | 0.675293941 |
| Race_NH Black | 2011 | 13.49000647 | 0.651169997 |
| Race_NH Black | 2012 | 13.20960791 | 0.631716429 |
| Race_NH Black | 2013 | 11.72732738 | 0.585270048 |
| Race_NH Black | 2014 | 10.95592664 | 0.557911616 |
| Race_NH Black | 2015 | 11.42938684 | 0.558142354 |
| Race_NH Black | 2016 | 12.16924588 | 0.56847411 |
| Race_NH Black | 2017 | 12.27845836 | 0.562184144 |
| Race_NH Black | 2018 | 11.7529297 | 0.542519479 |
| Race_NH Black | 2019 | 12.11255901 | 0.542776246 |
| Race_NH Black | 2020 | 14.00232323 | 0.573792331 |
| Race_NH Black | 2021 | 13.25613265 | 0.573627627 |
| Race_NH Black | 2022 | 12.12872202 | 0.532745103 |
| Race_NH Black | 2023 | 10.56104453 | 0.493488327 |
| Race_NH Other | 1999 | 4.446280607 | 0.806034938 |
| Race_NH Other | 2000 | 6.197010782 | 0.934977711 |
| Race_NH Other | 2001 | 5.49506731 | 0.83060758 |
| Race_NH Other | 2002 | 7.555806442 | 0.94502274 |
| Race_NH Other | 2003 | 7.085726678 | 0.889000881 |
| Race_NH Other | 2004 | 7.767957869 | 0.901083687 |
| Race_NH Other | 2005 | 7.396998621 | 0.84980575 |
| Race_NH Other | 2006 | 8.459797211 | 0.888426977 |
| Race_NH Other | 2007 | 8.246012153 | 0.845810472 |
| Race_NH Other | 2008 | 7.992049564 | 0.810087433 |
| Race_NH Other | 2009 | 6.993226029 | 0.731801496 |
| Race_NH Other | 2010 | 7.06291013 | 0.713379446 |
| Race_NH Other | 2011 | 8.058675229 | 0.733373145 |
| Race_NH Other | 2012 | 7.787001825 | 0.692543094 |
| Race_NH Other | 2013 | 5.462588129 | 0.558803269 |
| Race_NH Other | 2014 | 6.187678801 | 0.573080775 |
| Race_NH Other | 2015 | 6.088631947 | 0.543642051 |
| Race_NH Other | 2016 | 6.914329443 | 0.565495843 |
| Race_NH Other | 2017 | 6.432263578 | 0.52535666 |
| Race_NH Other | 2018 | 6.41584007 | 0.507394964 |
| Race_NH Other | 2019 | 5.74481932 | 0.469225374 |
| Race_NH Other | 2020 | 6.068589014 | 0.463484639 |
| Race_NH Other | 2021 | 6.293510851 | 0.456271631 |
| Race_NH Other | 2022 | 6.38923877 | 0.43487321 |
| Race_NH Other | 2023 | 6.781954086 | 0.441869871 |
| Race_NH White | 1999 | 14.2120967 | 0.218780122 |
| Race_NH White | 2000 | 15.3610029 | 0.226148573 |
| Race_NH White | 2001 | 15.2494583 | 0.224237955 |
| Race_NH White | 2002 | 15.81042075 | 0.227047998 |
| Race_NH White | 2003 | 15.67200219 | 0.224424224 |
| Race_NH White | 2004 | 15.91819276 | 0.225241697 |
| Race_NH White | 2005 | 16.25544702 | 0.225664499 |
| Race_NH White | 2006 | 16.17076422 | 0.222895738 |
| Race_NH White | 2007 | 15.47227485 | 0.21665651 |
| Race_NH White | 2008 | 15.05323089 | 0.21262742 |
| Race_NH White | 2009 | 14.09516155 | 0.203824705 |
| Race_NH White | 2010 | 14.13891267 | 0.203626945 |
| Race_NH White | 2011 | 13.16129988 | 0.193876216 |
| Race_NH White | 2012 | 12.32687723 | 0.187185874 |
| Race_NH White | 2013 | 11.35243021 | 0.177695813 |
| Race_NH White | 2014 | 11.23238728 | 0.175898586 |
| Race_NH White | 2015 | 11.92966301 | 0.179996696 |
| Race_NH White | 2016 | 12.79012597 | 0.184969303 |
| Race_NH White | 2017 | 12.74964795 | 0.182745053 |
| Race_NH White | 2018 | 12.89473221 | 0.182611852 |
| Race_NH White | 2019 | 13.0438159 | 0.181852198 |
| Race_NH White | 2020 | 13.69844681 | 0.185119942 |
| Race_NH White | 2021 | 14.23620056 | 0.196131549 |
| Race_NH White | 2022 | 13.67146552 | 0.184861781 |
| Race_NH White | 2023 | 12.8517074 | 0.180377147 |
| Sex_Both | 1999 | 13.96157254 | 0.20228722 |
| Sex_Both | 2000 | 14.88910267 | 0.207095289 |
| Sex_Both | 2001 | 14.80987192 | 0.2050655 |
| Sex_Both | 2002 | 15.45989659 | 0.208112584 |
| Sex_Both | 2003 | 15.39243727 | 0.205903195 |
| Sex_Both | 2004 | 15.56000392 | 0.205471376 |
| Sex_Both | 2005 | 15.83773621 | 0.205199788 |
| Sex_Both | 2006 | 15.70153027 | 0.202386116 |
| Sex_Both | 2007 | 15.08177215 | 0.196503179 |
| Sex_Both | 2008 | 14.63308062 | 0.191899897 |
| Sex_Both | 2009 | 13.70782462 | 0.184075642 |
| Sex_Both | 2010 | 13.59605319 | 0.182118013 |
| Sex_Both | 2011 | 12.81077573 | 0.174060266 |
| Sex_Both | 2012 | 12.01899789 | 0.167180678 |
| Sex_Both | 2013 | 11.01417697 | 0.15825599 |
| Sex_Both | 2014 | 10.88186312 | 0.155697112 |
| Sex_Both | 2015 | 11.44682499 | 0.158021624 |
| Sex_Both | 2016 | 12.16914205 | 0.160956412 |
| Sex_Both | 2017 | 12.13049666 | 0.158887546 |
| Sex_Both | 2018 | 12.15370536 | 0.157119014 |
| Sex_Both | 2019 | 12.15370536 | 0.154998532 |
| Sex_Both | 2020 | 13.0438159 | 0.159277017 |
| Sex_Both | 2021 | 13.36019357 | 0.166550112 |
| Sex_Both | 2022 | 12.84587536 | 0.156994278 |
| Sex_Both | 2023 | 12.04665967 | 0.152763003 |
| Sex_Female | 1999 | 11.79024195 | 0.229903931 |
| Sex_Female | 2000 | 12.92687723 | 0.239619179 |
| Sex_Female | 2001 | 12.76952204 | 0.236109677 |
| Sex_Female | 2002 | 13.45819211 | 0.241349674 |
| Sex_Female | 2003 | 13.28745969 | 0.238473932 |
| Sex_Female | 2004 | 13.40217006 | 0.239116208 |
| Sex_Female | 2005 | 13.8773434 | 0.24084303 |
| Sex_Female | 2006 | 13.413822 | 0.235100066 |
| Sex_Female | 2007 | 13.25952525 | 0.232406834 |
| Sex_Female | 2008 | 13.2038832 | 0.23087022 |
| Sex_Female | 2009 | 11.93066198 | 0.217454189 |
| Sex_Female | 2010 | 11.80745328 | 0.215071403 |
| Sex_Female | 2011 | 11.11501063 | 0.206161796 |
| Sex_Female | 2012 | 10.5392763 | 0.201212839 |
| Sex_Female | 2013 | 9.819126031 | 0.192080925 |
| Sex_Female | 2014 | 9.558497742 | 0.187066559 |
| Sex_Female | 2015 | 10.3526077 | 0.192891397 |
| Sex_Female | 2016 | 10.83167312 | 0.195237907 |
| Sex_Female | 2017 | 10.7625465 | 0.192969408 |
| Sex_Female | 2018 | 11.04588401 | 0.19338385 |
| Sex_Female | 2019 | 11.18586254 | 0.192617658 |
| Sex_Female | 2020 | 11.65642963 | 0.195045203 |
| Sex_Female | 2021 | 12.09684966 | 0.204969379 |
| Sex_Female | 2022 | 11.49530185 | 0.191221857 |
| Sex_Female | 2023 | 10.82412794 | 0.187102506 |
| Sex_Male | 1999 | 18.20162895 | 0.402164782 |
| Sex_Male | 2000 | 18.82055341 | 0.404101107 |
| Sex_Male | 2001 | 18.77302969 | 0.39957103 |
| Sex_Male | 2002 | 19.37030542 | 0.401856942 |
| Sex_Male | 2003 | 19.50028716 | 0.398120135 |
| Sex_Male | 2004 | 19.83931254 | 0.396092554 |
| Sex_Male | 2005 | 19.60272661 | 0.387639903 |
| Sex_Male | 2006 | 19.97473417 | 0.384911409 |
| Sex_Male | 2007 | 18.3890732 | 0.362722844 |
| Sex_Male | 2008 | 17.22001674 | 0.345774101 |
| Sex_Male | 2009 | 16.87004141 | 0.338482556 |
| Sex_Male | 2010 | 16.63789288 | 0.332009193 |
| Sex_Male | 2011 | 15.72563412 | 0.316427424 |
| Sex_Male | 2012 | 14.74235461 | 0.301983496 |
| Sex_Male | 2013 | 13.15008944 | 0.280683107 |
| Sex_Male | 2014 | 13.04342374 | 0.275501281 |
| Sex_Male | 2015 | 13.21327668 | 0.273462605 |
| Sex_Male | 2016 | 14.28211848 | 0.280354898 |
| Sex_Male | 2017 | 14.37662059 | 0.277519557 |
| Sex_Male | 2018 | 13.93376111 | 0.26901053 |
| Sex_Male | 2019 | 13.85696981 | 0.264613836 |
| Sex_Male | 2020 | 15.27256318 | 0.274540959 |
| Sex_Male | 2021 | 15.28350096 | 0.283519449 |
| Sex_Male | 2022 | 15.08689356 | 0.274486363 |
| Sex_Male | 2023 | 13.7541962 | 0.258935344 |
| State_Alabama | 1999 | 13.21395716 | 1.557805917 |
| State_Alabama | 2000 | 17.49905082 | 1.777325157 |
| State_Alabama | 2001 | 14.77321232 | 1.631855358 |
| State_Alabama | 2002 | 18.5276571 | 1.826525769 |
| State_Alabama | 2003 | 19.58319883 | 1.885964426 |
| State_Alabama | 2004 | 22.41876731 | 2.006928994 |
| State_Alabama | 2005 | 21.44668375 | 1.950982823 |
| State_Alabama | 2006 | 21.16377207 | 1.916777211 |
| State_Alabama | 2007 | 16.24786466 | 1.659580151 |
| State_Alabama | 2008 | 17.88236331 | 1.722623933 |
| State_Alabama | 2009 | 14.2224155 | 1.534354593 |
| State_Alabama | 2010 | 14.3075978 | 1.526437272 |
| State_Alabama | 2011 | 10.02539607 | 1.264378108 |
| State_Alabama | 2012 | 10.05347151 | 1.258838011 |
| State_Alabama | 2013 | 8.635966016 | 1.146139983 |
| State_Alabama | 2014 | 11.21939004 | 1.297955846 |
| State_Alabama | 2015 | 9.439845941 | 1.173851387 |
| State_Alabama | 2016 | 9.635074372 | 1.172784111 |
| State_Alabama | 2017 | 11.90068025 | 1.294331542 |
| State_Alabama | 2018 | 9.815154442 | 1.152446794 |
| State_Alabama | 2019 | 7.627442667 | 0.997737203 |
| State_Alabama | 2020 | 10.70497665 | 1.179315398 |
| State_Alabama | 2021 | 10.17048384 | 1.187025176 |
| State_Alabama | 2022 | 11.437609 | 1.197564249 |
| State_Alabama | 2023 | 8.596924958 | 1.039547411 |
| State_Alaska | 1999 |  |  |
| State_Alaska | 2000 |  | 11.54258772 |
| State_Alaska | 2001 |  |  |
| State_Alaska | 2002 |  |  |
| State_Alaska | 2003 |  |  |
| State_Alaska | 2004 |  |  |
| State_Alaska | 2005 |  |  |
| State_Alaska | 2006 |  |  |
| State_Alaska | 2007 |  |  |
| State_Alaska | 2008 |  | 8.275630607 |
| State_Alaska | 2009 |  |  |
| State_Alaska | 2010 |  |  |
| State_Alaska | 2011 |  |  |
| State_Alaska | 2012 |  |  |
| State_Alaska | 2013 |  |  |
| State_Alaska | 2014 |  |  |
| State_Alaska | 2015 |  |  |
| State_Alaska | 2016 |  |  |
| State_Alaska | 2017 |  |  |
| State_Alaska | 2018 |  |  |
| State_Alaska | 2019 |  | 5.085420078 |
| State_Alaska | 2020 |  |  |
| State_Alaska | 2021 |  |  |
| State_Alaska | 2022 |  |  |
| State_Alaska | 2023 |  |  |
| State_Arizona | 1999 | 8.454267984 | 1.189129643 |
| State_Arizona | 2000 | 10.32526441 | 1.295837234 |
| State_Arizona | 2001 | 8.117363556 | 1.141443758 |
| State_Arizona | 2002 | 7.641595558 | 1.095955955 |
| State_Arizona | 2003 | 8.875221741 | 1.168478639 |
| State_Arizona | 2004 | 7.436477666 | 1.05369243 |
| State_Arizona | 2005 | 12.95846828 | 1.360736476 |
| State_Arizona | 2006 | 8.864222457 | 1.100520341 |
| State_Arizona | 2007 | 7.222704768 | 0.974739865 |
| State_Arizona | 2008 | 8.596763141 | 1.0508214 |
| State_Arizona | 2009 | 7.130369447 | 0.937671688 |
| State_Arizona | 2010 | 10.36837855 | 1.119193578 |
| State_Arizona | 2011 | 13.97552644 | 1.262133233 |
| State_Arizona | 2012 | 9.804142998 | 1.041003765 |
| State_Arizona | 2013 | 8.700382567 | 0.957327181 |
| State_Arizona | 2014 | 8.784096555 | 0.949249941 |
| State_Arizona | 2015 | 10.40531081 | 1.003807521 |
| State_Arizona | 2016 | 12.06805141 | 1.052975455 |
| State_Arizona | 2017 | 9.431801269 | 0.927605716 |
| State_Arizona | 2018 | 9.16111819 | 0.894941498 |
| State_Arizona | 2019 | 8.261675672 | 0.835527703 |
| State_Arizona | 2020 | 8.829031164 | 0.842798844 |
| State_Arizona | 2021 | 9.453453512 | 0.915653691 |
| State_Arizona | 2022 | 8.380397626 | 0.818663614 |
| State_Arizona | 2023 | 9.26749556 | 0.851177907 |
| State_Arkansas | 1999 | 8.929395488 | 1.554548182 |
| State_Arkansas | 2000 | 6.966017906 | 1.366475507 |
| State_Arkansas | 2001 | 9.125119898 | 1.565223679 |
| State_Arkansas | 2002 | 11.76925453 | 1.774461647 |
| State_Arkansas | 2003 | 13.04122328 | 1.863116586 |
| State_Arkansas | 2004 | 13.89054829 | 1.926476282 |
| State_Arkansas | 2005 | 16.35677665 | 2.077827537 |
| State_Arkansas | 2006 | 18.51880893 | 2.198832241 |
| State_Arkansas | 2007 | 16.69396076 | 2.072058744 |
| State_Arkansas | 2008 | 17.29045451 | 2.11431418 |
| State_Arkansas | 2009 | 16.50924779 | 2.03423984 |
| State_Arkansas | 2010 | 16.86897742 | 2.049326254 |
| State_Arkansas | 2011 | 11.78601921 | 1.687186359 |
| State_Arkansas | 2012 | 11.41867588 | 1.652727809 |
| State_Arkansas | 2013 | 10.11529545 | 1.545781478 |
| State_Arkansas | 2014 | 11.69244242 | 1.658184117 |
| State_Arkansas | 2015 | 10.61179552 | 1.552952819 |
| State_Arkansas | 2016 | 10.41224917 | 1.522901229 |
| State_Arkansas | 2017 | 12.12090067 | 1.625006843 |
| State_Arkansas | 2018 | 10.84963509 | 1.507982629 |
| State_Arkansas | 2019 | 11.90483283 | 1.579907102 |
| State_Arkansas | 2020 | 9.216517744 | 1.37577206 |
| State_Arkansas | 2021 | 17.21983574 | 1.954902748 |
| State_Arkansas | 2022 | 15.39385273 | 1.772118647 |
| State_Arkansas | 2023 | 11.63215694 | 1.545834745 |
| State_California | 1999 | 13.79938429 | 0.631429198 |
| State_California | 2000 | 16.08204832 | 0.675506452 |
| State_California | 2001 | 15.77604744 | 0.659772668 |
| State_California | 2002 | 16.86620732 | 0.675825935 |
| State_California | 2003 | 17.72489564 | 0.685327749 |
| State_California | 2004 | 19.607783 | 0.715508148 |
| State_California | 2005 | 20.17523366 | 0.715282411 |
| State_California | 2006 | 19.7069407 | 0.699080547 |
| State_California | 2007 | 19.89700189 | 0.694636491 |
| State_California | 2008 | 20.12626947 | 0.689083329 |
| State_California | 2009 | 18.43383898 | 0.649393872 |
| State_California | 2010 | 18.62728061 | 0.647460873 |
| State_California | 2011 | 19.11151327 | 0.641727146 |
| State_California | 2012 | 18.37525798 | 0.622252419 |
| State_California | 2013 | 16.26149374 | 0.583706369 |
| State_California | 2014 | 16.2381777 | 0.574458425 |
| State_California | 2015 | 15.77215886 | 0.557236269 |
| State_California | 2016 | 16.09654754 | 0.553732003 |
| State_California | 2017 | 14.97766891 | 0.531053314 |
| State_California | 2018 | 15.54690286 | 0.533775809 |
| State_California | 2019 | 14.43659622 | 0.506552437 |
| State_California | 2020 | 16.440406 | 0.537660343 |
| State_California | 2021 | 15.11848109 | 0.533128502 |
| State_California | 2022 | 15.58356246 | 0.518762855 |
| State_California | 2023 | 13.58196531 | 0.487641002 |
| State_Colorado | 1999 | 15.62613361 | 2.001515357 |
| State_Colorado | 2000 | 12.29589652 | 1.757161785 |
| State_Colorado | 2001 | 16.68857372 | 2.023932858 |
| State_Colorado | 2002 | 17.55901197 | 2.055394014 |
| State_Colorado | 2003 | 12.59518236 | 1.714317273 |
| State_Colorado | 2004 | 15.93357146 | 1.904466166 |
| State_Colorado | 2005 | 18.90769133 | 2.039306604 |
| State_Colorado | 2006 | 14.8795317 | 1.779252917 |
| State_Colorado | 2007 | 18.63075271 | 1.955342993 |
| State_Colorado | 2008 | 18.10934648 | 1.891388003 |
| State_Colorado | 2009 | 15.20957494 | 1.716734748 |
| State_Colorado | 2010 | 12.13298545 | 1.510075033 |
| State_Colorado | 2011 | 13.45819562 | 1.551375559 |
| State_Colorado | 2012 | 10.83065847 | 1.384591127 |
| State_Colorado | 2013 | 11.7265094 | 1.401192705 |
| State_Colorado | 2014 | 10.99840612 | 1.344153635 |
| State_Colorado | 2015 | 15.02620727 | 1.535396461 |
| State_Colorado | 2016 | 27.19746045 | 2.07018973 |
| State_Colorado | 2017 | 26.64132523 | 2.004224953 |
| State_Colorado | 2018 | 23.57125061 | 1.860887274 |
| State_Colorado | 2019 | 23.13924076 | 1.807770698 |
| State_Colorado | 2020 | 27.91815371 | 1.956896523 |
| State_Colorado | 2021 | 25.9483866 | 1.962702265 |
| State_Colorado | 2022 | 21.0689213 | 1.681168057 |
| State_Colorado | 2023 | 21.74653409 | 1.712312991 |
| State_Connecticut | 1999 | 12.44730016 | 1.58202867 |
| State_Connecticut | 2000 | 14.06458515 | 1.671410516 |
| State_Connecticut | 2001 | 17.09949232 | 1.825099688 |
| State_Connecticut | 2002 | 14.89921031 | 1.679888351 |
| State_Connecticut | 2003 | 17.88542011 | 1.822859712 |
| State_Connecticut | 2004 | 13.48553189 | 1.587799805 |
| State_Connecticut | 2005 | 14.0872822 | 1.606250885 |
| State_Connecticut | 2006 | 12.92238183 | 1.514199759 |
| State_Connecticut | 2007 | 15.1681101 | 1.643564841 |
| State_Connecticut | 2008 | 13.25287522 | 1.520480737 |
| State_Connecticut | 2009 | 11.71200153 | 1.421642688 |
| State_Connecticut | 2010 | 9.967499069 | 1.298215976 |
| State_Connecticut | 2011 | 10.27094966 | 1.320283411 |
| State_Connecticut | 2012 | 8.227437524 | 1.138774574 |
| State_Connecticut | 2013 | 8.406198946 | 1.208002209 |
| State_Connecticut | 2014 | 10.31690125 | 1.334360463 |
| State_Connecticut | 2015 | 8.23052963 | 1.158058935 |
| State_Connecticut | 2016 | 11.48736452 | 1.37610311 |
| State_Connecticut | 2017 | 8.567400387 | 1.170027416 |
| State_Connecticut | 2018 | 9.291872076 | 1.210854182 |
| State_Connecticut | 2019 | 8.865708763 | 1.19208298 |
| State_Connecticut | 2020 | 8.395484484 | 1.156304192 |
| State_Connecticut | 2021 | 6.775895208 | 1.063423189 |
| State_Connecticut | 2022 | 6.441472554 | 1.021649412 |
| State_Connecticut | 2023 | 5.871585947 | 0.954496352 |
| State_Delaware | 1999 |  | 4.256552818 |
| State_Delaware | 2000 |  | 4.463747492 |
| State_Delaware | 2001 |  | 3.725268209 |
| State_Delaware | 2002 |  |  |
| State_Delaware | 2003 |  | 3.774842178 |
| State_Delaware | 2004 |  | 3.160671543 |
| State_Delaware | 2005 |  | 3.497934391 |
| State_Delaware | 2006 |  | 3.097895947 |
| State_Delaware | 2007 |  | 3.499245081 |
| State_Delaware | 2008 |  |  |
| State_Delaware | 2009 |  | 2.757473148 |
| State_Delaware | 2010 |  | 2.863476557 |
| State_Delaware | 2011 |  |  |
| State_Delaware | 2012 |  | 2.480815408 |
| State_Delaware | 2013 |  | 2.374675982 |
| State_Delaware | 2014 |  | 2.384546316 |
| State_Delaware | 2015 |  | 2.904961893 |
| State_Delaware | 2016 |  | 2.098481289 |
| State_Delaware | 2017 |  | 2.615057448 |
| State_Delaware | 2018 |  | 1.988351406 |
| State_Delaware | 2019 | 14.76761412 | 3.015136099 |
| State_Delaware | 2020 | 12.11646326 | 2.657385985 |
| State_Delaware | 2021 | 14.54857531 | 2.992310238 |
| State_Delaware | 2022 | 14.00303154 | 2.823582508 |
| State_Delaware | 2023 | 13.52506611 | 2.776144998 |
| State_District of Columbia | 1999 |  |  |
| State_District of Columbia | 2000 |  | 6.077776951 |
| State_District of Columbia | 2001 | 32.42814748 | 6.7643186 |
| State_District of Columbia | 2002 |  | 5.039313341 |
| State_District of Columbia | 2003 | 30.07550469 | 6.566018879 |
| State_District of Columbia | 2004 |  |  |
| State_District of Columbia | 2005 |  | 4.669065031 |
| State_District of Columbia | 2006 |  |  |
| State_District of Columbia | 2007 |  |  |
| State_District of Columbia | 2008 |  | 4.761479993 |
| State_District of Columbia | 2009 |  | 5.070302849 |
| State_District of Columbia | 2010 |  | 5.002583997 |
| State_District of Columbia | 2011 |  | 3.914939919 |
| State_District of Columbia | 2012 |  |  |
| State_District of Columbia | 2013 |  | 4.092034159 |
| State_District of Columbia | 2014 |  |  |
| State_District of Columbia | 2015 |  |  |
| State_District of Columbia | 2016 |  |  |
| State_District of Columbia | 2017 |  |  |
| State_District of Columbia | 2018 |  |  |
| State_District of Columbia | 2019 |  | 3.614756835 |
| State_District of Columbia | 2020 |  |  |
| State_District of Columbia | 2021 |  | 4.578835009 |
| State_District of Columbia | 2022 |  |  |
| State_District of Columbia | 2023 |  |  |
| State_Florida | 1999 | 9.188549632 | 0.580455332 |
| State_Florida | 2000 | 8.880658126 | 0.566477763 |
| State_Florida | 2001 | 7.62276627 | 0.521359809 |
| State_Florida | 2002 | 8.701926866 | 0.55186238 |
| State_Florida | 2003 | 8.07222995 | 0.527939615 |
| State_Florida | 2004 | 9.019138192 | 0.553227049 |
| State_Florida | 2005 | 10.19008528 | 0.582721838 |
| State_Florida | 2006 | 8.885537029 | 0.538819863 |
| State_Florida | 2007 | 8.614969936 | 0.524363517 |
| State_Florida | 2008 | 7.943750189 | 0.497754502 |
| State_Florida | 2009 | 7.14030825 | 0.46748148 |
| State_Florida | 2010 | 6.41449126 | 0.440391942 |
| State_Florida | 2011 | 5.332986386 | 0.391926305 |
| State_Florida | 2012 | 5.428606971 | 0.388666401 |
| State_Florida | 2013 | 5.277083834 | 0.375517762 |
| State_Florida | 2014 | 5.437377961 | 0.378599964 |
| State_Florida | 2015 | 6.311052831 | 0.399604829 |
| State_Florida | 2016 | 6.416658051 | 0.397513691 |
| State_Florida | 2017 | 6.534096207 | 0.394932167 |
| State_Florida | 2018 | 6.40705341 | 0.384818922 |
| State_Florida | 2019 | 6.815386413 | 0.389847821 |
| State_Florida | 2020 | 7.92675353 | 0.41529451 |
| State_Florida | 2021 | 7.98128927 | 0.429484454 |
| State_Florida | 2022 | 8.257961073 | 0.417434701 |
| State_Florida | 2023 | 6.670801637 | 0.373703321 |
| State_Georgia | 1999 | 13.55173757 | 1.363444909 |
| State_Georgia | 2000 | 13.65583216 | 1.366234541 |
| State_Georgia | 2001 | 17.12566428 | 1.51523444 |
| State_Georgia | 2002 | 16.06984756 | 1.456970011 |
| State_Georgia | 2003 | 13.56090422 | 1.330973207 |
| State_Georgia | 2004 | 15.48812169 | 1.409244872 |
| State_Georgia | 2005 | 14.14243693 | 1.325978225 |
| State_Georgia | 2006 | 12.2435554 | 1.213042719 |
| State_Georgia | 2007 | 11.51322032 | 1.158621157 |
| State_Georgia | 2008 | 11.34960726 | 1.132077866 |
| State_Georgia | 2009 | 11.10519132 | 1.107525084 |
| State_Georgia | 2010 | 12.9087006 | 1.189954525 |
| State_Georgia | 2011 | 10.9906921 | 1.070946595 |
| State_Georgia | 2012 | 10.96531661 | 1.05116138 |
| State_Georgia | 2013 | 8.624243924 | 0.921461537 |
| State_Georgia | 2014 | 7.72729885 | 0.860657018 |
| State_Georgia | 2015 | 7.023584251 | 0.804663197 |
| State_Georgia | 2016 | 11.19740714 | 1.001678993 |
| State_Georgia | 2017 | 11.40439134 | 0.992597212 |
| State_Georgia | 2018 | 10.11885337 | 0.913288478 |
| State_Georgia | 2019 | 10.78975463 | 0.925336843 |
| State_Georgia | 2020 | 12.81601311 | 0.988172949 |
| State_Georgia | 2021 | 12.50500167 | 1.008569954 |
| State_Georgia | 2022 | 10.82227964 | 0.894455952 |
| State_Georgia | 2023 | 13.12687723 | 0.981181278 |
| State_Hawaii | 1999 | 15.0515671 | 3.216215423 |
| State_Hawaii | 2000 | 21.3401317 | 3.779655561 |
| State_Hawaii | 2001 |  | 2.610498412 |
| State_Hawaii | 2002 | 14.2917341 | 2.919596857 |
| State_Hawaii | 2003 | 12.2824648 | 2.681225169 |
| State_Hawaii | 2004 | 13.04376656 | 2.72151838 |
| State_Hawaii | 2005 | 12.53766418 | 2.615712506 |
| State_Hawaii | 2006 | 15.69057448 | 2.868047916 |
| State_Hawaii | 2007 | 20.35354028 | 3.183684368 |
| State_Hawaii | 2008 | 10.03312927 | 2.196453486 |
| State_Hawaii | 2009 | 20.33885023 | 3.122892381 |
| State_Hawaii | 2010 | 9.736043188 | 2.095062429 |
| State_Hawaii | 2011 |  | 1.744073515 |
| State_Hawaii | 2012 |  | 1.771185246 |
| State_Hawaii | 2013 |  | 1.584826935 |
| State_Hawaii | 2014 | 8.850576073 | 1.849700949 |
| State_Hawaii | 2015 |  | 1.591288299 |
| State_Hawaii | 2016 | 7.739800109 | 1.744672012 |
| State_Hawaii | 2017 | 10.6682904 | 1.930086546 |
| State_Hawaii | 2018 | 6.877078692 | 1.53373621 |
| State_Hawaii | 2019 | 12.28355544 | 2.134108375 |
| State_Hawaii | 2020 | 11.50357686 | 1.98495937 |
| State_Hawaii | 2021 | 7.31322664 | 1.571456158 |
| State_Hawaii | 2022 | 12.62771439 | 2.023639111 |
| State_Hawaii | 2023 | 12.13660488 | 1.979293204 |
| State_Idaho | 1999 | 22.9905249 | 4.002191642 |
| State_Idaho | 2000 | 17.03519877 | 3.407048233 |
| State_Idaho | 2001 | 18.61889545 | 3.518706859 |
| State_Idaho | 2002 | 23.51688534 | 3.91973309 |
| State_Idaho | 2003 | 19.50003367 | 3.503122175 |
| State_Idaho | 2004 | 17.54253864 | 3.316384455 |
| State_Idaho | 2005 | 18.72542015 | 3.365781421 |
| State_Idaho | 2006 | 20.02345704 | 3.388558276 |
| State_Idaho | 2007 | 16.01888027 | 2.980400467 |
| State_Idaho | 2008 | 13.87710441 | 2.784546665 |
| State_Idaho | 2009 |  | 2.324784396 |
| State_Idaho | 2010 | 10.77606123 | 2.421592029 |
| State_Idaho | 2011 |  | 2.22916333 |
| State_Idaho | 2012 | 9.824520094 | 2.208600742 |
| State_Idaho | 2013 | 9.96111819 | 2.186148374 |
| State_Idaho | 2014 | 8.995466483 | 2.021809302 |
| State_Idaho | 2015 | 12.02881719 | 2.366992178 |
| State_Idaho | 2016 | 18.32242485 | 2.876671627 |
| State_Idaho | 2017 | 13.31699058 | 2.370662679 |
| State_Idaho | 2018 | 11.7970185 | 2.232923664 |
| State_Idaho | 2019 | 10.4188377 | 2.052407937 |
| State_Idaho | 2020 | 18.38225434 | 2.690180401 |
| State_Idaho | 2021 | 21.1575495 | 2.909494527 |
| State_Idaho | 2022 | 20.6237262 | 2.77002231 |
| State_Idaho | 2023 | 18.43542327 | 2.608888236 |
| State_Illinois | 1999 | 13.94519487 | 0.957813215 |
| State_Illinois | 2000 | 15.51675229 | 1.006061448 |
| State_Illinois | 2001 | 13.35490684 | 0.926218988 |
| State_Illinois | 2002 | 15.38844651 | 0.996076761 |
| State_Illinois | 2003 | 13.77440095 | 0.935756623 |
| State_Illinois | 2004 | 15.45806396 | 0.984495355 |
| State_Illinois | 2005 | 14.62892804 | 0.951547038 |
| State_Illinois | 2006 | 14.97534895 | 0.953038242 |
| State_Illinois | 2007 | 12.78817034 | 0.883429885 |
| State_Illinois | 2008 | 13.77639025 | 0.910936906 |
| State_Illinois | 2009 | 12.18097148 | 0.849971908 |
| State_Illinois | 2010 | 11.28669619 | 0.812247042 |
| State_Illinois | 2011 | 9.699083098 | 0.748853839 |
| State_Illinois | 2012 | 9.671813473 | 0.75261503 |
| State_Illinois | 2013 | 8.800535733 | 0.700034319 |
| State_Illinois | 2014 | 7.358080568 | 0.631470227 |
| State_Illinois | 2015 | 8.330633455 | 0.680528999 |
| State_Illinois | 2016 | 8.962616657 | 0.694042792 |
| State_Illinois | 2017 | 8.548224776 | 0.675898855 |
| State_Illinois | 2018 | 7.775337726 | 0.63223774 |
| State_Illinois | 2019 | 7.119159005 | 0.5997309 |
| State_Illinois | 2020 | 9.407532086 | 0.690891444 |
| State_Illinois | 2021 | 8.576051883 | 0.678385442 |
| State_Illinois | 2022 | 7.462744803 | 0.609489657 |
| State_Illinois | 2023 | 8.022815611 | 0.639069405 |
| State_Indiana | 1999 | 14.4420906 | 1.396253053 |
| State_Indiana | 2000 | 15.64613071 | 1.446500762 |
| State_Indiana | 2001 | 17.76486202 | 1.52897469 |
| State_Indiana | 2002 | 18.35878747 | 1.546125537 |
| State_Indiana | 2003 | 17.85619485 | 1.514627587 |
| State_Indiana | 2004 | 17.44370926 | 1.49038738 |
| State_Indiana | 2005 | 17.54097283 | 1.482849887 |
| State_Indiana | 2006 | 20.2731707 | 1.579182397 |
| State_Indiana | 2007 | 17.19698552 | 1.439940232 |
| State_Indiana | 2008 | 15.70616667 | 1.375253257 |
| State_Indiana | 2009 | 15.50257858 | 1.34934232 |
| State_Indiana | 2010 | 15.34361058 | 1.346729796 |
| State_Indiana | 2011 | 14.67009238 | 1.305310326 |
| State_Indiana | 2012 | 15.06362335 | 1.296328181 |
| State_Indiana | 2013 | 15.96065658 | 1.337409031 |
| State_Indiana | 2014 | 12.49580485 | 1.154346483 |
| State_Indiana | 2015 | 16.66605415 | 1.332964482 |
| State_Indiana | 2016 | 14.31865507 | 1.233495456 |
| State_Indiana | 2017 | 14.26018141 | 1.218532661 |
| State_Indiana | 2018 | 16.21301337 | 1.289562318 |
| State_Indiana | 2019 | 16.2532344 | 1.271393568 |
| State_Indiana | 2020 | 13.65956243 | 1.166871161 |
| State_Indiana | 2021 | 14.7142635 | 1.245893838 |
| State_Indiana | 2022 | 10.50764293 | 1.01028422 |
| State_Indiana | 2023 | 10.56820972 | 1.020727757 |
| State_Iowa | 1999 | 16.5100901 | 1.841883153 |
| State_Iowa | 2000 | 18.1677743 | 1.911657655 |
| State_Iowa | 2001 | 18.37412315 | 1.949180871 |
| State_Iowa | 2002 | 17.70880279 | 1.910090417 |
| State_Iowa | 2003 | 15.78892518 | 1.786380587 |
| State_Iowa | 2004 | 17.87212519 | 1.899071157 |
| State_Iowa | 2005 | 17.06258205 | 1.833878072 |
| State_Iowa | 2006 | 15.00020578 | 1.737030306 |
| State_Iowa | 2007 | 14.9106719 | 1.70665996 |
| State_Iowa | 2008 | 16.76442403 | 1.796748811 |
| State_Iowa | 2009 | 15.87584844 | 1.75457807 |
| State_Iowa | 2010 | 13.722857 | 1.62881812 |
| State_Iowa | 2011 | 13.40309889 | 1.606806192 |
| State_Iowa | 2012 | 13.43681603 | 1.611416839 |
| State_Iowa | 2013 | 13.8009936 | 1.612739932 |
| State_Iowa | 2014 | 14.34613585 | 1.654329656 |
| State_Iowa | 2015 | 11.3597944 | 1.483312407 |
| State_Iowa | 2016 | 12.88405493 | 1.55621266 |
| State_Iowa | 2017 | 17.69297745 | 1.799039634 |
| State_Iowa | 2018 | 14.83909367 | 1.632053698 |
| State_Iowa | 2019 | 16.03835519 | 1.702457734 |
| State_Iowa | 2020 | 13.66595547 | 1.565747468 |
| State_Iowa | 2021 | 16.15826647 | 1.775756366 |
| State_Iowa | 2022 | 16.11802744 | 1.692797323 |
| State_Iowa | 2023 | 16.5186836 | 1.728127141 |
| State_Kansas | 1999 | 13.8777449 | 1.893834621 |
| State_Kansas | 2000 | 15.40540668 | 1.995327715 |
| State_Kansas | 2001 | 15.65961177 | 1.99513593 |
| State_Kansas | 2002 | 17.18077668 | 2.09137818 |
| State_Kansas | 2003 | 15.48020634 | 1.97525064 |
| State_Kansas | 2004 | 16.79997614 | 2.075674172 |
| State_Kansas | 2005 | 15.14219794 | 1.950070609 |
| State_Kansas | 2006 | 14.87838821 | 1.934400152 |
| State_Kansas | 2007 | 15.54389891 | 1.959841353 |
| State_Kansas | 2008 | 12.40512116 | 1.736951748 |
| State_Kansas | 2009 | 11.38534738 | 1.678297574 |
| State_Kansas | 2010 | 11.38118614 | 1.644458906 |
| State_Kansas | 2011 | 11.27912318 | 1.651306506 |
| State_Kansas | 2012 | 8.44618964 | 1.409680766 |
| State_Kansas | 2013 | 7.473274759 | 1.342979203 |
| State_Kansas | 2014 | 8.454675809 | 1.394374651 |
| State_Kansas | 2015 | 6.790072421 | 1.218301526 |
| State_Kansas | 2016 | 10.17359162 | 1.509951599 |
| State_Kansas | 2017 | 9.601093214 | 1.469668662 |
| State_Kansas | 2018 | 12.44438554 | 1.641846476 |
| State_Kansas | 2019 | 11.61093754 | 1.600489991 |
| State_Kansas | 2020 | 9.225246411 | 1.388079624 |
| State_Kansas | 2021 | 11.63379408 | 1.60921427 |
| State_Kansas | 2022 | 9.98664685 | 1.463436586 |
| State_Kansas | 2023 | 9.915580268 | 1.466115019 |
| State_Kentucky | 1999 | 17.24347378 | 1.904778345 |
| State_Kentucky | 2000 | 16.74462874 | 1.861340103 |
| State_Kentucky | 2001 | 17.00926861 | 1.867982402 |
| State_Kentucky | 2002 | 22.13440932 | 2.130802307 |
| State_Kentucky | 2003 | 20.65052649 | 2.055649263 |
| State_Kentucky | 2004 | 19.46579949 | 1.977618648 |
| State_Kentucky | 2005 | 23.12786662 | 2.13886776 |
| State_Kentucky | 2006 | 19.70371975 | 1.951467106 |
| State_Kentucky | 2007 | 21.33766956 | 2.00856599 |
| State_Kentucky | 2008 | 20.37383551 | 1.953151401 |
| State_Kentucky | 2009 | 16.09418759 | 1.727204345 |
| State_Kentucky | 2010 | 21.92731428 | 1.996697841 |
| State_Kentucky | 2011 | 18.41309989 | 1.817478577 |
| State_Kentucky | 2012 | 20.70342509 | 1.911902077 |
| State_Kentucky | 2013 | 15.93049735 | 1.665445609 |
| State_Kentucky | 2014 | 13.6490783 | 1.520298823 |
| State_Kentucky | 2015 | 16.22802774 | 1.636070077 |
| State_Kentucky | 2016 | 17.22557613 | 1.671004756 |
| State_Kentucky | 2017 | 14.47366949 | 1.520693532 |
| State_Kentucky | 2018 | 18.33153702 | 1.69169951 |
| State_Kentucky | 2019 | 17.1779874 | 1.609081795 |
| State_Kentucky | 2020 | 15.24080329 | 1.50218149 |
| State_Kentucky | 2021 | 18.3054932 | 1.715070792 |
| State_Kentucky | 2022 | 17.75933046 | 1.634281026 |
| State_Kentucky | 2023 | 15.52556046 | 1.542023273 |
| State_Louisiana | 1999 | 10.61430863 | 1.487350082 |
| State_Louisiana | 2000 | 8.150660719 | 1.289306795 |
| State_Louisiana | 2001 | 9.256726615 | 1.380539009 |
| State_Louisiana | 2002 | 10.03668017 | 1.421280747 |
| State_Louisiana | 2003 | 11.37316228 | 1.508402432 |
| State_Louisiana | 2004 | 9.553284676 | 1.380528598 |
| State_Louisiana | 2005 | 12.87688623 | 1.585932172 |
| State_Louisiana | 2006 | 11.84540603 | 1.555809536 |
| State_Louisiana | 2007 | 13.75160591 | 1.655881594 |
| State_Louisiana | 2008 | 11.86618393 | 1.520229307 |
| State_Louisiana | 2009 | 12.52131784 | 1.554001742 |
| State_Louisiana | 2010 | 11.88290793 | 1.499186181 |
| State_Louisiana | 2011 | 12.06761224 | 1.499184322 |
| State_Louisiana | 2012 | 13.06117688 | 1.542733325 |
| State_Louisiana | 2013 | 14.5577298 | 1.602950292 |
| State_Louisiana | 2014 | 10.64136592 | 1.36839521 |
| State_Louisiana | 2015 | 11.67582856 | 1.393027491 |
| State_Louisiana | 2016 | 12.83538772 | 1.451094769 |
| State_Louisiana | 2017 | 11.3779252 | 1.346856079 |
| State_Louisiana | 2018 | 11.47475943 | 1.338602664 |
| State_Louisiana | 2019 | 11.48247345 | 1.328301468 |
| State_Louisiana | 2020 | 15.54191311 | 1.523312031 |
| State_Louisiana | 2021 | 14.76577447 | 1.545237001 |
| State_Louisiana | 2022 | 12.2349619 | 1.358627368 |
| State_Louisiana | 2023 | 9.121090316 | 1.184008958 |
| State_Maine | 1999 | 21.24782585 | 3.40317336 |
| State_Maine | 2000 | 20.3961009 | 3.309707393 |
| State_Maine | 2001 | 24.83707911 | 3.624316354 |
| State_Maine | 2002 | 22.91708482 | 3.456369253 |
| State_Maine | 2003 | 15.5347996 | 2.837378242 |
| State_Maine | 2004 | 20.20736068 | 3.196896364 |
| State_Maine | 2005 | 15.52649793 | 2.790496972 |
| State_Maine | 2006 | 13.19365957 | 2.542448759 |
| State_Maine | 2007 | 13.9675456 | 2.598298261 |
| State_Maine | 2008 | 16.32227801 | 2.765971706 |
| State_Maine | 2009 | 10.74546332 | 2.247658778 |
| State_Maine | 2010 | 11.5435734 | 2.319795873 |
| State_Maine | 2011 | 14.0500228 | 2.495855185 |
| State_Maine | 2012 | 10.2089784 | 2.188757162 |
| State_Maine | 2013 |  | 1.81197007 |
| State_Maine | 2014 |  | 1.679584486 |
| State_Maine | 2015 | 10.85695928 | 2.188369253 |
| State_Maine | 2016 | 12.60417504 | 2.277788899 |
| State_Maine | 2017 | 11.61890271 | 2.174495381 |
| State_Maine | 2018 | 14.81839176 | 2.449666231 |
| State_Maine | 2019 | 8.386462344 | 1.83687447 |
| State_Maine | 2020 | 13.78770803 | 2.307416196 |
| State_Maine | 2021 | 12.13001284 | 2.221423578 |
| State_Maine | 2022 | 8.429469151 | 1.761323033 |
| State_Maine | 2023 | 12.06883572 | 2.111134104 |
| State_Maryland | 1999 | 20.62732644 | 1.918046893 |
| State_Maryland | 2000 | 18.42845941 | 1.79168294 |
| State_Maryland | 2001 | 16.76915842 | 1.695247618 |
| State_Maryland | 2002 | 16.87548996 | 1.680120953 |
| State_Maryland | 2003 | 14.04730718 | 1.515208511 |
| State_Maryland | 2004 | 13.56007057 | 1.470915872 |
| State_Maryland | 2005 | 17.88961502 | 1.668322845 |
| State_Maryland | 2006 | 11.98377876 | 1.349328394 |
| State_Maryland | 2007 | 12.0251061 | 1.330361429 |
| State_Maryland | 2008 | 11.8659571 | 1.322531085 |
| State_Maryland | 2009 | 11.67612624 | 1.288216068 |
| State_Maryland | 2010 | 10.00640145 | 1.187578674 |
| State_Maryland | 2011 | 9.371421318 | 1.138874944 |
| State_Maryland | 2012 | 9.203189852 | 1.104380108 |
| State_Maryland | 2013 | 7.225371048 | 0.968832324 |
| State_Maryland | 2014 | 4.901733011 | 0.783805439 |
| State_Maryland | 2015 | 5.627227999 | 0.820734877 |
| State_Maryland | 2016 | 6.210718668 | 0.870785701 |
| State_Maryland | 2017 | 8.109864204 | 0.985590505 |
| State_Maryland | 2018 | 8.66640141 | 0.988742483 |
| State_Maryland | 2019 | 8.679824475 | 0.989149281 |
| State_Maryland | 2020 | 10.56515479 | 1.072192195 |
| State_Maryland | 2021 | 9.329514983 | 1.034890934 |
| State_Maryland | 2022 | 10.49446469 | 1.052295467 |
| State_Maryland | 2023 | 9.345677991 | 0.993244777 |
| State_Massachusetts | 1999 | 14.93902001 | 1.286905285 |
| State_Massachusetts | 2000 | 15.83734054 | 1.316321547 |
| State_Massachusetts | 2001 | 13.41146205 | 1.205386068 |
| State_Massachusetts | 2002 | 14.58643639 | 1.252783668 |
| State_Massachusetts | 2003 | 15.23824435 | 1.277730926 |
| State_Massachusetts | 2004 | 15.76095707 | 1.291641615 |
| State_Massachusetts | 2005 | 12.43666287 | 1.135672892 |
| State_Massachusetts | 2006 | 11.25182339 | 1.073876541 |
| State_Massachusetts | 2007 | 10.57309213 | 1.029031819 |
| State_Massachusetts | 2008 | 10.80190605 | 1.039183403 |
| State_Massachusetts | 2009 | 11.13187563 | 1.050302673 |
| State_Massachusetts | 2010 | 9.875090085 | 0.986008959 |
| State_Massachusetts | 2011 | 11.59563602 | 1.058396401 |
| State_Massachusetts | 2012 | 8.492594885 | 0.912086998 |
| State_Massachusetts | 2013 | 8.738289482 | 0.905394603 |
| State_Massachusetts | 2014 | 7.466505234 | 0.84818913 |
| State_Massachusetts | 2015 | 6.724433573 | 0.781311647 |
| State_Massachusetts | 2016 | 6.861531158 | 0.785295878 |
| State_Massachusetts | 2017 | 7.150201222 | 0.793095917 |
| State_Massachusetts | 2018 | 7.302484347 | 0.813614513 |
| State_Massachusetts | 2019 | 7.640964414 | 0.819925182 |
| State_Massachusetts | 2020 | 8.018160027 | 0.830125722 |
| State_Massachusetts | 2021 | 10.43839682 | 0.978338032 |
| State_Massachusetts | 2022 | 8.303321508 | 0.841193176 |
| State_Massachusetts | 2023 | 10.52662538 | 0.951054262 |
| State_Michigan | 1999 | 14.71820141 | 1.119544478 |
| State_Michigan | 2000 | 15.4735093 | 1.138079847 |
| State_Michigan | 2001 | 14.49511221 | 1.092847175 |
| State_Michigan | 2002 | 13.6785692 | 1.052417567 |
| State_Michigan | 2003 | 13.51139471 | 1.036444162 |
| State_Michigan | 2004 | 14.37371181 | 1.059826528 |
| State_Michigan | 2005 | 13.16825041 | 1.007289905 |
| State_Michigan | 2006 | 12.99782432 | 0.988928069 |
| State_Michigan | 2007 | 13.28417092 | 0.99447937 |
| State_Michigan | 2008 | 13.138253 | 0.981609474 |
| State_Michigan | 2009 | 12.84453005 | 0.956086091 |
| State_Michigan | 2010 | 9.259463049 | 0.804097529 |
| State_Michigan | 2011 | 10.45931923 | 0.855135001 |
| State_Michigan | 2012 | 10.21634026 | 0.842419015 |
| State_Michigan | 2013 | 9.057421592 | 0.800336612 |
| State_Michigan | 2014 | 12.13814567 | 0.907487367 |
| State_Michigan | 2015 | 8.893321202 | 0.766153566 |
| State_Michigan | 2016 | 9.999536755 | 0.804199386 |
| State_Michigan | 2017 | 11.22522209 | 0.849263118 |
| State_Michigan | 2018 | 9.598810437 | 0.773030498 |
| State_Michigan | 2019 | 12.34954061 | 0.870416053 |
| State_Michigan | 2020 | 11.46364065 | 0.839703901 |
| State_Michigan | 2021 | 12.2430524 | 0.891342565 |
| State_Michigan | 2022 | 12.39801748 | 0.872618638 |
| State_Michigan | 2023 | 11.98680703 | 0.86816861 |
| State_Minnesota | 1999 | 15.94918915 | 1.56839434 |
| State_Minnesota | 2000 | 16.27947371 | 1.593671664 |
| State_Minnesota | 2001 | 14.71439515 | 1.500695212 |
| State_Minnesota | 2002 | 18.45043132 | 1.649893487 |
| State_Minnesota | 2003 | 17.90581802 | 1.630254422 |
| State_Minnesota | 2004 | 19.48594111 | 1.692849656 |
| State_Minnesota | 2005 | 17.56631465 | 1.594080562 |
| State_Minnesota | 2006 | 20.05699202 | 1.708503068 |
| State_Minnesota | 2007 | 14.72153953 | 1.422755779 |
| State_Minnesota | 2008 | 14.22808222 | 1.396577552 |
| State_Minnesota | 2009 | 14.87783423 | 1.415648721 |
| State_Minnesota | 2010 | 17.56571134 | 1.525052579 |
| State_Minnesota | 2011 | 14.06027658 | 1.361393408 |
| State_Minnesota | 2012 | 15.27352216 | 1.423054893 |
| State_Minnesota | 2013 | 13.23466491 | 1.281414517 |
| State_Minnesota | 2014 | 18.73414531 | 1.534856541 |
| State_Minnesota | 2015 | 15.64400975 | 1.38358888 |
| State_Minnesota | 2016 | 19.73040406 | 1.51565917 |
| State_Minnesota | 2017 | 19.51960377 | 1.518208834 |
| State_Minnesota | 2018 | 22.23792843 | 1.585031663 |
| State_Minnesota | 2019 | 24.62977361 | 1.669213565 |
| State_Minnesota | 2020 | 25.84057974 | 1.703591174 |
| State_Minnesota | 2021 | 28.43108079 | 1.835149827 |
| State_Minnesota | 2022 | 24.19436765 | 1.613874497 |
| State_Minnesota | 2023 | 22.80569408 | 1.5773374 |
| State_Mississippi | 1999 | 15.88456377 | 2.183360209 |
| State_Mississippi | 2000 | 15.40517985 | 2.1380351 |
| State_Mississippi | 2001 | 21.3628519 | 2.518627268 |
| State_Mississippi | 2002 | 21.43141753 | 2.526239057 |
| State_Mississippi | 2003 | 15.0060135 | 2.122297212 |
| State_Mississippi | 2004 | 16.96168058 | 2.247407466 |
| State_Mississippi | 2005 | 24.08868526 | 2.660647389 |
| State_Mississippi | 2006 | 19.57019458 | 2.391780561 |
| State_Mississippi | 2007 | 18.6650402 | 2.33321031 |
| State_Mississippi | 2008 | 24.66926996 | 2.647406497 |
| State_Mississippi | 2009 | 23.76538138 | 2.595591703 |
| State_Mississippi | 2010 | 19.84853368 | 2.358125943 |
| State_Mississippi | 2011 | 20.42811777 | 2.361687653 |
| State_Mississippi | 2012 | 19.22830624 | 2.272337643 |
| State_Mississippi | 2013 | 17.23840453 | 2.111698422 |
| State_Mississippi | 2014 | 13.01699128 | 1.825440041 |
| State_Mississippi | 2015 | 17.93659692 | 2.132820159 |
| State_Mississippi | 2016 | 20.11874229 | 2.240790998 |
| State_Mississippi | 2017 | 16.16212488 | 1.980767051 |
| State_Mississippi | 2018 | 14.55936857 | 1.84288616 |
| State_Mississippi | 2019 | 22.799714 | 2.301118551 |
| State_Mississippi | 2020 | 26.39451216 | 2.466619941 |
| State_Mississippi | 2021 | 22.79046854 | 2.385751078 |
| State_Mississippi | 2022 | 22.56901648 | 2.285591368 |
| State_Mississippi | 2023 | 21.68139122 | 2.243934867 |
| State_Missouri | 1999 | 16.59148413 | 1.467341492 |
| State_Missouri | 2000 | 13.82139151 | 1.337190644 |
| State_Missouri | 2001 | 12.47152351 | 1.260546094 |
| State_Missouri | 2002 | 16.77454032 | 1.460790898 |
| State_Missouri | 2003 | 16.92487413 | 1.462896812 |
| State_Missouri | 2004 | 16.14986963 | 1.422549586 |
| State_Missouri | 2005 | 19.29463259 | 1.551034947 |
| State_Missouri | 2006 | 18.6161775 | 1.506739946 |
| State_Missouri | 2007 | 17.94877267 | 1.473047091 |
| State_Missouri | 2008 | 17.07382616 | 1.426024566 |
| State_Missouri | 2009 | 18.07385983 | 1.46576165 |
| State_Missouri | 2010 | 13.97783073 | 1.281059028 |
| State_Missouri | 2011 | 11.28210562 | 1.121802367 |
| State_Missouri | 2012 | 13.15524101 | 1.218731391 |
| State_Missouri | 2013 | 14.01647612 | 1.252668435 |
| State_Missouri | 2014 | 7.922196627 | 0.927777015 |
| State_Missouri | 2015 | 12.77307132 | 1.170212106 |
| State_Missouri | 2016 | 10.93216045 | 1.080305116 |
| State_Missouri | 2017 | 11.13400875 | 1.068023427 |
| State_Missouri | 2018 | 12.33999747 | 1.109263123 |
| State_Missouri | 2019 | 12.15087375 | 1.09627104 |
| State_Missouri | 2020 | 10.3439527 | 1.005141217 |
| State_Missouri | 2021 | 12.13964764 | 1.13492861 |
| State_Missouri | 2022 | 11.96798991 | 1.085636318 |
| State_Missouri | 2023 | 10.30429616 | 1.017321375 |
| State_Montana | 1999 |  | 3.077234681 |
| State_Montana | 2000 |  | 3.331941899 |
| State_Montana | 2001 | 15.95391722 | 3.568845882 |
| State_Montana | 2002 | 22.67968674 | 4.214355646 |
| State_Montana | 2003 | 21.21146229 | 4.011543485 |
| State_Montana | 2004 |  | 2.67058049 |
| State_Montana | 2005 | 15.83624476 | 3.38171934 |
| State_Montana | 2006 | 16.98149435 | 3.474364537 |
| State_Montana | 2007 |  | 2.918384545 |
| State_Montana | 2008 | 16.06954706 | 3.365224377 |
| State_Montana | 2009 |  | 2.721931648 |
| State_Montana | 2010 | 15.64390944 | 3.210370587 |
| State_Montana | 2011 |  | 2.872759167 |
| State_Montana | 2012 |  |  |
| State_Montana | 2013 |  | 2.650044795 |
| State_Montana | 2014 |  | 2.309771313 |
| State_Montana | 2015 |  | 2.153110854 |
| State_Montana | 2016 |  | 2.31056955 |
| State_Montana | 2017 | 12.19523521 | 2.671696486 |
| State_Montana | 2018 |  | 2.419602967 |
| State_Montana | 2019 |  | 2.465234584 |
| State_Montana | 2020 |  | 2.072861609 |
| State_Montana | 2021 | 14.34268526 | 2.881039691 |
| State_Montana | 2022 |  | 2.323962238 |
| State_Montana | 2023 |  | 2.22344183 |
| State_Nebraska | 1999 | 16.25304756 | 2.518042467 |
| State_Nebraska | 2000 | 12.32581091 | 2.18823967 |
| State_Nebraska | 2001 | 16.12563412 | 2.498441144 |
| State_Nebraska | 2002 | 17.73954382 | 2.659073951 |
| State_Nebraska | 2003 | 18.31591349 | 2.657151277 |
| State_Nebraska | 2004 | 17.50188945 | 2.591106228 |
| State_Nebraska | 2005 | 20.07332153 | 2.747097379 |
| State_Nebraska | 2006 | 21.52569118 | 2.817765506 |
| State_Nebraska | 2007 | 13.32857236 | 2.209610104 |
| State_Nebraska | 2008 | 18.95788366 | 2.676422707 |
| State_Nebraska | 2009 | 20.05192347 | 2.730827011 |
| State_Nebraska | 2010 | 16.6876622 | 2.488833346 |
| State_Nebraska | 2011 | 19.06027846 | 2.555610649 |
| State_Nebraska | 2012 | 21.8602843 | 2.793416462 |
| State_Nebraska | 2013 | 13.96018141 | 2.2137694 |
| State_Nebraska | 2014 | 11.41656194 | 1.958245721 |
| State_Nebraska | 2015 | 11.97371111 | 2.02528955 |
| State_Nebraska | 2016 | 16.67988737 | 2.449775663 |
| State_Nebraska | 2017 | 21.88432151 | 2.696380414 |
| State_Nebraska | 2018 | 16.5293244 | 2.34843363 |
| State_Nebraska | 2019 | 17.32687209 | 2.362837944 |
| State_Nebraska | 2020 | 25.43785827 | 2.854819959 |
| State_Nebraska | 2021 | 24.75801087 | 2.958128554 |
| State_Nebraska | 2022 | 22.20075367 | 2.687575918 |
| State_Nebraska | 2023 | 20.34730787 | 2.592824665 |
| State_Nevada | 1999 |  | 2.428536466 |
| State_Nevada | 2000 |  |  |
| State_Nevada | 2001 |  |  |
| State_Nevada | 2002 |  | 2.245822258 |
| State_Nevada | 2003 |  | 2.119974901 |
| State_Nevada | 2004 |  | 1.991827137 |
| State_Nevada | 2005 |  | 1.632973411 |
| State_Nevada | 2006 |  | 1.600419862 |
| State_Nevada | 2007 |  | 1.714050872 |
| State_Nevada | 2008 |  | 1.476234552 |
| State_Nevada | 2009 |  | 1.244824755 |
| State_Nevada | 2010 |  | 1.241211616 |
| State_Nevada | 2011 |  | 1.346305989 |
| State_Nevada | 2012 |  | 1.152983112 |
| State_Nevada | 2013 |  | 1.292846874 |
| State_Nevada | 2014 |  | 1.113185835 |
| State_Nevada | 2015 |  | 1.311481659 |
| State_Nevada | 2016 |  | 1.258190538 |
| State_Nevada | 2017 |  | 1.169343507 |
| State_Nevada | 2018 | 5.656161418 | 1.218394018 |
| State_Nevada | 2019 | 5.039664247 | 1.133927163 |
| State_Nevada | 2020 | 6.83499136 | 1.3007395 |
| State_Nevada | 2021 | 7.569291009 | 1.400997702 |
| State_Nevada | 2022 | 7.565944243 | 1.328924124 |
| State_Nevada | 2023 | 8.840183613 | 1.464886329 |
| State_New Hampshire | 1999 | 20.049401 | 3.7232614 |
| State_New Hampshire | 2000 | 21.06331257 | 3.783391591 |
| State_New Hampshire | 2001 | 23.17647701 | 3.917957588 |
| State_New Hampshire | 2002 | 20.86683752 | 3.689321568 |
| State_New Hampshire | 2003 | 24.91069014 | 3.990080795 |
| State_New Hampshire | 2004 | 28.54052525 | 4.210946821 |
| State_New Hampshire | 2005 | 26.18399389 | 3.99808354 |
| State_New Hampshire | 2006 | 18.23191702 | 3.283211609 |
| State_New Hampshire | 2007 | 16.08003237 | 2.994163617 |
| State_New Hampshire | 2008 | 17.56360768 | 3.167467223 |
| State_New Hampshire | 2009 | 15.50074829 | 2.998792805 |
| State_New Hampshire | 2010 | 21.26039163 | 3.426030729 |
| State_New Hampshire | 2011 | 14.1122777 | 2.734885817 |
| State_New Hampshire | 2012 | 9.896122647 | 2.228327808 |
| State_New Hampshire | 2013 | 12.28802068 | 2.482235707 |
| State_New Hampshire | 2014 | 10.16144019 | 2.241717802 |
| State_New Hampshire | 2015 | 13.80806712 | 2.550721325 |
| State_New Hampshire | 2016 | 14.09917897 | 2.652455014 |
| State_New Hampshire | 2017 | 13.98497159 | 2.582583816 |
| State_New Hampshire | 2018 | 10.78174012 | 2.221617542 |
| State_New Hampshire | 2019 | 13.21919688 | 2.431542501 |
| State_New Hampshire | 2020 | 12.61841257 | 2.356259746 |
| State_New Hampshire | 2021 | 13.36775676 | 2.488587361 |
| State_New Hampshire | 2022 | 11.59216976 | 2.194060689 |
| State_New Hampshire | 2023 | 11.20262886 | 2.128441771 |
| State_New Jersey | 1999 | 9.933336915 | 0.951629766 |
| State_New Jersey | 2000 | 10.73521889 | 0.980115902 |
| State_New Jersey | 2001 | 12.83464924 | 1.062358687 |
| State_New Jersey | 2002 | 12.23949448 | 1.030931275 |
| State_New Jersey | 2003 | 11.40134506 | 0.988898157 |
| State_New Jersey | 2004 | 12.55665997 | 1.03253592 |
| State_New Jersey | 2005 | 12.40255006 | 1.016680373 |
| State_New Jersey | 2006 | 10.84907994 | 0.949738737 |
| State_New Jersey | 2007 | 11.49351506 | 0.964065029 |
| State_New Jersey | 2008 | 10.13256477 | 0.896029932 |
| State_New Jersey | 2009 | 10.23377841 | 0.897146924 |
| State_New Jersey | 2010 | 13.29232643 | 1.019630171 |
| State_New Jersey | 2011 | 13.07644824 | 1.008813863 |
| State_New Jersey | 2012 | 11.72660457 | 0.939431255 |
| State_New Jersey | 2013 | 11.46785474 | 0.931744525 |
| State_New Jersey | 2014 | 9.780826961 | 0.847666052 |
| State_New Jersey | 2015 | 9.620140679 | 0.827612514 |
| State_New Jersey | 2016 | 9.787162009 | 0.826734458 |
| State_New Jersey | 2017 | 10.78275827 | 0.862028108 |
| State_New Jersey | 2018 | 8.137706981 | 0.737883987 |
| State_New Jersey | 2019 | 9.610882361 | 0.793377098 |
| State_New Jersey | 2020 | 9.735804198 | 0.803014146 |
| State_New Jersey | 2021 | 7.602438515 | 0.727175473 |
| State_New Jersey | 2022 | 8.288276972 | 0.734468191 |
| State_New Jersey | 2023 | 6.843201359 | 0.672380098 |
| State_New Mexico | 1999 | 13.12083402 | 2.62791937 |
| State_New Mexico | 2000 | 15.09957744 | 2.762072767 |
| State_New Mexico | 2001 |  | 2.092365291 |
| State_New Mexico | 2002 | 12.15711947 | 2.43266589 |
| State_New Mexico | 2003 | 15.1344016 | 2.677772725 |
| State_New Mexico | 2004 | 11.24209925 | 2.296407623 |
| State_New Mexico | 2005 | 11.36429682 | 2.273555044 |
| State_New Mexico | 2006 | 12.62340513 | 2.346218413 |
| State_New Mexico | 2007 |  | 1.752570667 |
| State_New Mexico | 2008 | 9.014151955 | 1.923051124 |
| State_New Mexico | 2009 | 10.17629087 | 1.999763369 |
| State_New Mexico | 2010 |  | 1.680161834 |
| State_New Mexico | 2011 |  | 1.498653788 |
| State_New Mexico | 2012 | 7.534491871 | 1.647895672 |
| State_New Mexico | 2013 |  | 1.382300667 |
| State_New Mexico | 2014 | 6.750090379 | 1.516310717 |
| State_New Mexico | 2015 | 8.393201707 | 1.68499319 |
| State_New Mexico | 2016 | 9.25048674 | 1.752674023 |
| State_New Mexico | 2017 | 6.647638766 | 1.458696718 |
| State_New Mexico | 2018 | 9.733517912 | 1.725564034 |
| State_New Mexico | 2019 | 8.729349656 | 1.624477814 |
| State_New Mexico | 2020 | 13.79843465 | 2.015130347 |
| State_New Mexico | 2021 | 12.34576802 | 1.957862749 |
| State_New Mexico | 2022 | 15.48811071 | 2.131384546 |
| State_New Mexico | 2023 | 8.200336735 | 1.554344394 |
| State_New York | 1999 | 10.24904627 | 0.648340246 |
| State_New York | 2000 | 10.01269932 | 0.634706787 |
| State_New York | 2001 | 11.1928009 | 0.664340924 |
| State_New York | 2002 | 9.947394634 | 0.621887002 |
| State_New York | 2003 | 9.468056551 | 0.602738053 |
| State_New York | 2004 | 7.568457358 | 0.536821444 |
| State_New York | 2005 | 7.852793839 | 0.543903506 |
| State_New York | 2006 | 7.897378615 | 0.543957163 |
| State_New York | 2007 | 8.88849164 | 0.567577598 |
| State_New York | 2008 | 7.437856637 | 0.521017085 |
| State_New York | 2009 | 7.648629092 | 0.522865007 |
| State_New York | 2010 | 8.368387204 | 0.544859918 |
| State_New York | 2011 | 6.918147866 | 0.489998546 |
| State_New York | 2012 | 7.116600059 | 0.494933063 |
| State_New York | 2013 | 5.993176688 | 0.447759095 |
| State_New York | 2014 | 6.022036444 | 0.457139639 |
| State_New York | 2015 | 6.971515793 | 0.479066854 |
| State_New York | 2016 | 6.12221744 | 0.444798361 |
| State_New York | 2017 | 6.212385971 | 0.442320001 |
| State_New York | 2018 | 6.244093003 | 0.438113618 |
| State_New York | 2019 | 7.652901173 | 0.484814189 |
| State_New York | 2020 | 7.600832715 | 0.47368362 |
| State_New York | 2021 | 8.396940628 | 0.507233164 |
| State_New York | 2022 | 7.655686948 | 0.47181863 |
| State_New York | 2023 | 7.22143313 | 0.460269359 |
| State_North Carolina | 1999 | 16.08282912 | 1.342644044 |
| State_North Carolina | 2000 | 16.60223226 | 1.361703842 |
| State_North Carolina | 2001 | 21.3199716 | 1.524798231 |
| State_North Carolina | 2002 | 20.69151266 | 1.484075086 |
| State_North Carolina | 2003 | 19.5695176 | 1.42882376 |
| State_North Carolina | 2004 | 18.88133486 | 1.389259586 |
| State_North Carolina | 2005 | 19.09473993 | 1.382292242 |
| State_North Carolina | 2006 | 19.61399504 | 1.37029823 |
| State_North Carolina | 2007 | 16.98874769 | 1.256277341 |
| State_North Carolina | 2008 | 17.47530031 | 1.258434715 |
| State_North Carolina | 2009 | 16.30013913 | 1.196580535 |
| State_North Carolina | 2010 | 15.12196535 | 1.141485261 |
| State_North Carolina | 2011 | 12.06894889 | 1.007588317 |
| State_North Carolina | 2012 | 11.63726618 | 0.9692046 |
| State_North Carolina | 2013 | 11.30695028 | 0.942322646 |
| State_North Carolina | 2014 | 9.891203752 | 0.865271513 |
| State_North Carolina | 2015 | 12.62260515 | 0.964460889 |
| State_North Carolina | 2016 | 12.38974247 | 0.941830784 |
| State_North Carolina | 2017 | 12.04796497 | 0.924704693 |
| State_North Carolina | 2018 | 11.64394404 | 0.883598067 |
| State_North Carolina | 2019 | 12.67403311 | 0.906548471 |
| State_North Carolina | 2020 | 11.72111534 | 0.861941079 |
| State_North Carolina | 2021 | 12.72422662 | 0.936757822 |
| State_North Carolina | 2022 | 11.13323309 | 0.837813784 |
| State_North Carolina | 2023 | 14.25008944 | 0.952454632 |
| State_North Dakota | 1999 |  | 4.00905572 |
| State_North Dakota | 2000 | 19.32899375 | 4.249440945 |
| State_North Dakota | 2001 | 21.94113371 | 4.510395151 |
| State_North Dakota | 2002 | 18.25949415 | 4.009289422 |
| State_North Dakota | 2003 | 24.4078272 | 4.746036833 |
| State_North Dakota | 2004 | 23.89857823 | 4.646749611 |
| State_North Dakota | 2005 | 29.62858335 | 5.130032993 |
| State_North Dakota | 2006 | 33.52318599 | 5.567689145 |
| State_North Dakota | 2007 | 34.61149315 | 5.700985228 |
| State_North Dakota | 2008 | 25.62294609 | 4.739227457 |
| State_North Dakota | 2009 | 19.18166155 | 3.952783303 |
| State_North Dakota | 2010 | 31.75187107 | 5.372824637 |
| State_North Dakota | 2011 | 37.64848855 | 5.587990966 |
| State_North Dakota | 2012 | 32.20463617 | 5.260868211 |
| State_North Dakota | 2013 | 24.44340901 | 4.480263196 |
| State_North Dakota | 2014 | 24.33171357 | 4.552841206 |
| State_North Dakota | 2015 | 20.90207465 | 4.210323493 |
| State_North Dakota | 2016 |  | 2.856213061 |
| State_North Dakota | 2017 | 23.72711317 | 4.542316712 |
| State_North Dakota | 2018 | 22.37477066 | 4.252180437 |
| State_North Dakota | 2019 | 20.79376597 | 4.100976462 |
| State_North Dakota | 2020 | 24.81679836 | 4.328576896 |
| State_North Dakota | 2021 | 17.82103723 | 3.945159826 |
| State_North Dakota | 2022 | 23.91589619 | 4.288276352 |
| State_North Dakota | 2023 | 22.10865453 | 4.220020736 |
| State_Ohio | 1999 | 20.18577578 | 1.175861793 |
| State_Ohio | 2000 | 19.53998952 | 1.149852586 |
| State_Ohio | 2001 | 20.3811817 | 1.165515902 |
| State_Ohio | 2002 | 19.64635473 | 1.136568076 |
| State_Ohio | 2003 | 19.01661549 | 1.109241663 |
| State_Ohio | 2004 | 21.10148513 | 1.163594915 |
| State_Ohio | 2005 | 22.1772073 | 1.184009666 |
| State_Ohio | 2006 | 23.02511452 | 1.194542162 |
| State_Ohio | 2007 | 21.40120031 | 1.14215004 |
| State_Ohio | 2008 | 18.73214384 | 1.061299446 |
| State_Ohio | 2009 | 16.98691857 | 0.999646453 |
| State_Ohio | 2010 | 16.97343401 | 0.993114691 |
| State_Ohio | 2011 | 16.2665658 | 0.961554296 |
| State_Ohio | 2012 | 14.22479345 | 0.909342577 |
| State_Ohio | 2013 | 12.06308317 | 0.823310356 |
| State_Ohio | 2014 | 11.92937468 | 0.819774483 |
| State_Ohio | 2015 | 11.78990779 | 0.808471201 |
| State_Ohio | 2016 | 14.15134892 | 0.882994803 |
| State_Ohio | 2017 | 13.23258979 | 0.846198473 |
| State_Ohio | 2018 | 12.81581996 | 0.824353774 |
| State_Ohio | 2019 | 11.00778393 | 0.760348264 |
| State_Ohio | 2020 | 13.65046944 | 0.839623264 |
| State_Ohio | 2021 | 11.49701499 | 0.800008901 |
| State_Ohio | 2022 | 11.81655843 | 0.785001608 |
| State_Ohio | 2023 | 11.34072028 | 0.776725925 |
| State_Oklahoma | 1999 | 10.74608582 | 1.53564688 |
| State_Oklahoma | 2000 | 14.31553513 | 1.776307104 |
| State_Oklahoma | 2001 | 18.01037492 | 1.989161265 |
| State_Oklahoma | 2002 | 15.67486746 | 1.860423694 |
| State_Oklahoma | 2003 | 19.00740884 | 2.049733393 |
| State_Oklahoma | 2004 | 15.25859712 | 1.837063784 |
| State_Oklahoma | 2005 | 14.00583532 | 1.750946966 |
| State_Oklahoma | 2006 | 15.43209381 | 1.819114482 |
| State_Oklahoma | 2007 | 13.81154787 | 1.713686938 |
| State_Oklahoma | 2008 | 12.70796844 | 1.628274992 |
| State_Oklahoma | 2009 | 11.35254971 | 1.532182853 |
| State_Oklahoma | 2010 | 15.19474507 | 1.768297418 |
| State_Oklahoma | 2011 | 9.951124903 | 1.395608896 |
| State_Oklahoma | 2012 | 12.54070461 | 1.572025406 |
| State_Oklahoma | 2013 | 12.19117078 | 1.539700776 |
| State_Oklahoma | 2014 | 14.70681348 | 1.670546281 |
| State_Oklahoma | 2015 | 14.75527468 | 1.665739954 |
| State_Oklahoma | 2016 | 18.48372848 | 1.844039438 |
| State_Oklahoma | 2017 | 19.76553034 | 1.882948545 |
| State_Oklahoma | 2018 | 18.99561941 | 1.82375799 |
| State_Oklahoma | 2019 | 17.44361409 | 1.724462048 |
| State_Oklahoma | 2020 | 19.26463519 | 1.801229301 |
| State_Oklahoma | 2021 | 23.70723487 | 2.072666415 |
| State_Oklahoma | 2022 | 20.86605134 | 1.893704451 |
| State_Oklahoma | 2023 | 20.23433847 | 1.870796954 |
| State_Oregon | 1999 | 14.12397547 | 1.766003835 |
| State_Oregon | 2000 | 18.28900819 | 1.996154187 |
| State_Oregon | 2001 | 17.58558543 | 1.931273365 |
| State_Oregon | 2002 | 17.8136237 | 1.9338597 |
| State_Oregon | 2003 | 14.96306236 | 1.753643992 |
| State_Oregon | 2004 | 18.74112015 | 1.947173879 |
| State_Oregon | 2005 | 19.41599651 | 1.95549287 |
| State_Oregon | 2006 | 20.0029256 | 1.968414325 |
| State_Oregon | 2007 | 20.61592284 | 1.985277377 |
| State_Oregon | 2008 | 17.23212817 | 1.810486751 |
| State_Oregon | 2009 | 15.55873229 | 1.683703203 |
| State_Oregon | 2010 | 15.67374899 | 1.69945718 |
| State_Oregon | 2011 | 17.70500938 | 1.768105138 |
| State_Oregon | 2012 | 16.86154308 | 1.709524878 |
| State_Oregon | 2013 | 17.16860445 | 1.696473675 |
| State_Oregon | 2014 | 13.43148698 | 1.49199563 |
| State_Oregon | 2015 | 19.20990396 | 1.765984821 |
| State_Oregon | 2016 | 20.38975369 | 1.793374794 |
| State_Oregon | 2017 | 22.54014105 | 1.886174679 |
| State_Oregon | 2018 | 25.67106214 | 1.976577159 |
| State_Oregon | 2019 | 22.47613583 | 1.829471454 |
| State_Oregon | 2020 | 24.42228642 | 1.896900506 |
| State_Oregon | 2021 | 29.49408703 | 2.170342576 |
| State_Oregon | 2022 | 29.91480812 | 2.080805316 |
| State_Oregon | 2023 | 28.81067003 | 2.069620356 |
| State_Pennsylvania | 1999 | 13.60000327 | 0.838844821 |
| State_Pennsylvania | 2000 | 14.08027018 | 0.846149162 |
| State_Pennsylvania | 2001 | 14.34513688 | 0.851546619 |
| State_Pennsylvania | 2002 | 13.6070368 | 0.823918178 |
| State_Pennsylvania | 2003 | 15.97217102 | 0.886842652 |
| State_Pennsylvania | 2004 | 15.92457364 | 0.879785814 |
| State_Pennsylvania | 2005 | 15.2708802 | 0.859096997 |
| State_Pennsylvania | 2006 | 14.47250167 | 0.825082379 |
| State_Pennsylvania | 2007 | 13.96662028 | 0.806551633 |
| State_Pennsylvania | 2008 | 12.65870376 | 0.767580719 |
| State_Pennsylvania | 2009 | 12.7287592 | 0.76330349 |
| State_Pennsylvania | 2010 | 11.30130274 | 0.709365826 |
| State_Pennsylvania | 2011 | 11.89118294 | 0.73542532 |
| State_Pennsylvania | 2012 | 9.25205887 | 0.644852669 |
| State_Pennsylvania | 2013 | 9.177661192 | 0.633420204 |
| State_Pennsylvania | 2014 | 9.115414941 | 0.627862124 |
| State_Pennsylvania | 2015 | 9.472935454 | 0.644646742 |
| State_Pennsylvania | 2016 | 9.968770708 | 0.659081515 |
| State_Pennsylvania | 2017 | 9.357787095 | 0.625131814 |
| State_Pennsylvania | 2018 | 9.92733954 | 0.650859299 |
| State_Pennsylvania | 2019 | 8.950118907 | 0.614007039 |
| State_Pennsylvania | 2020 | 10.37387644 | 0.659439492 |
| State_Pennsylvania | 2021 | 9.666116588 | 0.655003748 |
| State_Pennsylvania | 2022 | 9.557832926 | 0.632405602 |
| State_Pennsylvania | 2023 | 9.527033208 | 0.635783933 |
| State_Rhode Island | 1999 | 15.23236365 | 3.046938269 |
| State_Rhode Island | 2000 | 16.72965856 | 3.16228264 |
| State_Rhode Island | 2001 |  | 2.34965036 |
| State_Rhode Island | 2002 | 17.89014515 | 3.283306979 |
| State_Rhode Island | 2003 | 19.36709733 | 3.332622049 |
| State_Rhode Island | 2004 | 18.72612496 | 3.278407929 |
| State_Rhode Island | 2005 | 17.20640215 | 3.223397994 |
| State_Rhode Island | 2006 | 16.51626566 | 3.034670745 |
| State_Rhode Island | 2007 | 12.24195825 | 2.64602809 |
| State_Rhode Island | 2008 | 13.56005257 | 2.753832984 |
| State_Rhode Island | 2009 | 18.41325305 | 3.264729278 |
| State_Rhode Island | 2010 | 14.98803003 | 2.947385714 |
| State_Rhode Island | 2011 | 19.38701375 | 3.314626049 |
| State_Rhode Island | 2012 | 16.32044607 | 3.015600082 |
| State_Rhode Island | 2013 | 17.19019168 | 3.090069597 |
| State_Rhode Island | 2014 | 19.21921162 | 3.264497213 |
| State_Rhode Island | 2015 | 12.19833597 | 2.628644856 |
| State_Rhode Island | 2016 | 14.43650689 | 2.870189758 |
| State_Rhode Island | 2017 | 11.46179353 | 2.380090232 |
| State_Rhode Island | 2018 | 17.50584304 | 3.070605263 |
| State_Rhode Island | 2019 | 14.68836419 | 2.829467852 |
| State_Rhode Island | 2020 | 11.24373873 | 2.490168284 |
| State_Rhode Island | 2021 | 13.12331066 | 2.695837275 |
| State_Rhode Island | 2022 | 25.96739852 | 3.621815371 |
| State_Rhode Island | 2023 | 19.049886 | 3.137969497 |
| State_South Carolina | 1999 | 17.20347958 | 2.006028297 |
| State_South Carolina | 2000 | 18.91769046 | 2.069672845 |
| State_South Carolina | 2001 | 17.19623489 | 1.9643508 |
| State_South Carolina | 2002 | 17.99204512 | 1.981644588 |
| State_South Carolina | 2003 | 23.28017758 | 2.246466636 |
| State_South Carolina | 2004 | 21.9704221 | 2.148337653 |
| State_South Carolina | 2005 | 16.54522691 | 1.851883813 |
| State_South Carolina | 2006 | 21.86483255 | 2.077626638 |
| State_South Carolina | 2007 | 16.80205759 | 1.783636608 |
| State_South Carolina | 2008 | 20.38267151 | 1.937264854 |
| State_South Carolina | 2009 | 14.15924043 | 1.595760403 |
| State_South Carolina | 2010 | 14.27067419 | 1.580614397 |
| State_South Carolina | 2011 | 15.15341189 | 1.601015573 |
| State_South Carolina | 2012 | 15.36589748 | 1.589285619 |
| State_South Carolina | 2013 | 11.98064082 | 1.378970328 |
| State_South Carolina | 2014 | 15.54944965 | 1.557914915 |
| State_South Carolina | 2015 | 16.35207009 | 1.565962208 |
| State_South Carolina | 2016 | 14.93240365 | 1.482014809 |
| State_South Carolina | 2017 | 19.62967072 | 1.651326562 |
| State_South Carolina | 2018 | 17.98392164 | 1.558907855 |
| State_South Carolina | 2019 | 12.10138224 | 1.257560971 |
| State_South Carolina | 2020 | 17.13107402 | 1.459719245 |
| State_South Carolina | 2021 | 18.32584365 | 1.591961071 |
| State_South Carolina | 2022 | 18.02521764 | 1.485649425 |
| State_South Carolina | 2023 | 17.17716077 | 1.4613283 |
| State_South Dakota | 1999 |  | 3.124966777 |
| State_South Dakota | 2000 | 21.07457118 | 4.233194432 |
| State_South Dakota | 2001 |  |  |
| State_South Dakota | 2002 |  | 2.878765688 |
| State_South Dakota | 2003 | 23.7752042 | 4.366132909 |
| State_South Dakota | 2004 | 17.95100096 | 3.767758021 |
| State_South Dakota | 2005 | 21.11508054 | 4.094708285 |
| State_South Dakota | 2006 | 24.19316568 | 4.378184151 |
| State_South Dakota | 2007 | 28.50387149 | 4.731095383 |
| State_South Dakota | 2008 | 26.47670033 | 4.462594104 |
| State_South Dakota | 2009 | 23.16419626 | 4.143925706 |
| State_South Dakota | 2010 | 23.78025708 | 4.264822334 |
| State_South Dakota | 2011 | 26.77748253 | 4.414829914 |
| State_South Dakota | 2012 | 21.62841545 | 3.951330101 |
| State_South Dakota | 2013 | 16.80062646 | 3.494830201 |
| State_South Dakota | 2014 | 15.6207309 | 3.323196667 |
| State_South Dakota | 2015 | 22.5861843 | 4.022556029 |
| State_South Dakota | 2016 | 24.78648198 | 4.230956544 |
| State_South Dakota | 2017 | 18.42049542 | 3.617631923 |
| State_South Dakota | 2018 | 28.02390508 | 4.459791894 |
| State_South Dakota | 2019 | 21.89805956 | 3.939794049 |
| State_South Dakota | 2020 | 21.92923694 | 3.875413319 |
| State_South Dakota | 2021 | 24.53474769 | 4.31165112 |
| State_South Dakota | 2022 | 28.92867268 | 4.395526945 |
| State_South Dakota | 2023 | 23.33426901 | 4.022272137 |
| State_Tennessee | 1999 | 16.77406164 | 1.585867988 |
| State_Tennessee | 2000 | 18.14931799 | 1.643475641 |
| State_Tennessee | 2001 | 18.89655689 | 1.664332417 |
| State_Tennessee | 2002 | 17.09912448 | 1.581528437 |
| State_Tennessee | 2003 | 21.52291405 | 1.770115796 |
| State_Tennessee | 2004 | 19.27181254 | 1.65975683 |
| State_Tennessee | 2005 | 22.73591129 | 1.787195036 |
| State_Tennessee | 2006 | 22.34348664 | 1.745494619 |
| State_Tennessee | 2007 | 26.73402137 | 1.891148818 |
| State_Tennessee | 2008 | 25.00696571 | 1.806608398 |
| State_Tennessee | 2009 | 22.93473132 | 1.716378183 |
| State_Tennessee | 2010 | 19.60405975 | 1.571589628 |
| State_Tennessee | 2011 | 19.73911939 | 1.558156747 |
| State_Tennessee | 2012 | 15.76333502 | 1.371400804 |
| State_Tennessee | 2013 | 15.16954706 | 1.334427136 |
| State_Tennessee | 2014 | 15.71446951 | 1.341163657 |
| State_Tennessee | 2015 | 16.55923528 | 1.3526843 |
| State_Tennessee | 2016 | 14.29282943 | 1.248248334 |
| State_Tennessee | 2017 | 15.11016025 | 1.263379237 |
| State_Tennessee | 2018 | 14.51218837 | 1.225561492 |
| State_Tennessee | 2019 | 15.15312356 | 1.232814028 |
| State_Tennessee | 2020 | 13.88348178 | 1.171823287 |
| State_Tennessee | 2021 | 12.87747505 | 1.159516273 |
| State_Tennessee | 2022 | 14.11021824 | 1.176369119 |
| State_Tennessee | 2023 | 11.66887804 | 1.07121377 |
| State_Texas | 1999 | 15.55083727 | 0.89681999 |
| State_Texas | 2000 | 16.83282947 | 0.925801903 |
| State_Texas | 2001 | 15.59186412 | 0.886086672 |
| State_Texas | 2002 | 16.62996419 | 0.909458974 |
| State_Texas | 2003 | 16.46577798 | 0.898891413 |
| State_Texas | 2004 | 16.44366343 | 0.891087777 |
| State_Texas | 2005 | 15.21413184 | 0.843067394 |
| State_Texas | 2006 | 16.8919593 | 0.874869409 |
| State_Texas | 2007 | 15.81719378 | 0.835104092 |
| State_Texas | 2008 | 16.1597191 | 0.834953105 |
| State_Texas | 2009 | 15.09214894 | 0.796152597 |
| State_Texas | 2010 | 15.23756737 | 0.79113462 |
| State_Texas | 2011 | 13.93609323 | 0.739746483 |
| State_Texas | 2012 | 12.93986231 | 0.704603895 |
| State_Texas | 2013 | 12.87623358 | 0.690972065 |
| State_Texas | 2014 | 11.70428751 | 0.646553403 |
| State_Texas | 2015 | 14.39843465 | 0.707143539 |
| State_Texas | 2016 | 14.39832732 | 0.693758698 |
| State_Texas | 2017 | 15.21881175 | 0.703674834 |
| State_Texas | 2018 | 14.71993022 | 0.68175193 |
| State_Texas | 2019 | 15.69154563 | 0.694352841 |
| State_Texas | 2020 | 15.61448167 | 0.683260473 |
| State_Texas | 2021 | 16.44507023 | 0.726688455 |
| State_Texas | 2022 | 15.4781684 | 0.678574242 |
| State_Texas | 2023 | 14.86912124 | 0.661156574 |
| State_Utah | 1999 |  | 1.769593398 |
| State_Utah | 2000 | 16.3949719 | 2.994929287 |
| State_Utah | 2001 |  | 2.248783297 |
| State_Utah | 2002 | 11.90792775 | 2.484190756 |
| State_Utah | 2003 | 10.73245743 | 2.342504052 |
| State_Utah | 2004 |  | 1.864270402 |
| State_Utah | 2005 | 13.00917625 | 2.504235525 |
| State_Utah | 2006 | 10.63043165 | 2.217119066 |
| State_Utah | 2007 | 9.913389155 | 2.114031371 |
| State_Utah | 2008 |  | 1.853096295 |
| State_Utah | 2009 |  | 1.633202165 |
| State_Utah | 2010 |  | 1.794439468 |
| State_Utah | 2011 |  | 1.63147989 |
| State_Utah | 2012 | 7.64264902 | 1.717224422 |
| State_Utah | 2013 |  | 1.186778084 |
| State_Utah | 2014 | 7.984789202 | 1.706883474 |
| State_Utah | 2015 | 9.80471615 | 1.858526001 |
| State_Utah | 2016 | 10.99104193 | 1.949212684 |
| State_Utah | 2017 | 12.42305881 | 2.049442078 |
| State_Utah | 2018 | 10.64243342 | 1.882243456 |
| State_Utah | 2019 | 14.25280506 | 2.12895518 |
| State_Utah | 2020 | 15.28799987 | 2.169191531 |
| State_Utah | 2021 | 14.36224438 | 2.176753552 |
| State_Utah | 2022 | 12.04222226 | 1.912239811 |
| State_Utah | 2023 | 11.22967517 | 1.861035946 |
| State_Vermont | 1999 |  | 4.959962548 |
| State_Vermont | 2000 | 27.91701606 | 5.955828898 |
| State_Vermont | 2001 |  | 5.021461589 |
| State_Vermont | 2002 | 28.18169873 | 5.880232708 |
| State_Vermont | 2003 |  | 5.097853287 |
| State_Vermont | 2004 |  | 4.475411686 |
| State_Vermont | 2005 | 30.11988953 | 5.914045929 |
| State_Vermont | 2006 | 22.43618232 | 5.026864937 |
| State_Vermont | 2007 | 32.21805058 | 5.999963889 |
| State_Vermont | 2008 |  | 4.822857501 |
| State_Vermont | 2009 |  | 4.471783212 |
| State_Vermont | 2010 | 27.01308398 | 5.44601209 |
| State_Vermont | 2011 | 24.8475541 | 5.232906998 |
| State_Vermont | 2012 |  | 4.581702116 |
| State_Vermont | 2013 |  | 4.429266606 |
| State_Vermont | 2014 | 28.21351331 | 5.398041042 |
| State_Vermont | 2015 | 23.34829025 | 4.824801892 |
| State_Vermont | 2016 | 23.99124584 | 4.860532963 |
| State_Vermont | 2017 | 23.73425803 | 4.689343536 |
| State_Vermont | 2018 | 32.51954624 | 5.526041314 |
| State_Vermont | 2019 | 21.76131181 | 4.456488861 |
| State_Vermont | 2020 | 20.27896393 | 4.240738688 |
| State_Vermont | 2021 | 18.40204379 | 4.125852002 |
| State_Vermont | 2022 | 18.74554377 | 3.996589477 |
| State_Vermont | 2023 | 18.77645363 | 4.026565789 |
| State_Virginia | 1999 | 12.63675102 | 1.31242037 |
| State_Virginia | 2000 | 17.24921066 | 1.526499717 |
| State_Virginia | 2001 | 13.10674263 | 1.318853611 |
| State_Virginia | 2002 | 16.41161873 | 1.451982352 |
| State_Virginia | 2003 | 15.38049701 | 1.393450232 |
| State_Virginia | 2004 | 17.8788739 | 1.490610356 |
| State_Virginia | 2005 | 12.51816423 | 1.228003536 |
| State_Virginia | 2006 | 13.33137965 | 1.248684742 |
| State_Virginia | 2007 | 13.94045697 | 1.262857655 |
| State_Virginia | 2008 | 11.74369289 | 1.147118704 |
| State_Virginia | 2009 | 13.8842696 | 1.230181093 |
| State_Virginia | 2010 | 13.10212423 | 1.1753011 |
| State_Virginia | 2011 | 11.99100545 | 1.113222638 |
| State_Virginia | 2012 | 9.720686 | 0.98676944 |
| State_Virginia | 2013 | 7.105055455 | 0.830758268 |
| State_Virginia | 2014 | 8.044295509 | 0.877798544 |
| State_Virginia | 2015 | 8.736073349 | 0.901810349 |
| State_Virginia | 2016 | 8.815304099 | 0.895064966 |
| State_Virginia | 2017 | 9.276208557 | 0.904586862 |
| State_Virginia | 2018 | 7.858341067 | 0.817490454 |
| State_Virginia | 2019 | 8.511985164 | 0.832986491 |
| State_Virginia | 2020 | 9.058117748 | 0.85069782 |
| State_Virginia | 2021 | 11.57442878 | 0.995473098 |
| State_Virginia | 2022 | 10.92866052 | 0.921628009 |
| State_Virginia | 2023 | 11.13694418 | 0.928506001 |
| State_Washington | 1999 | 19.88580361 | 1.724465932 |
| State_Washington | 2000 | 20.43581612 | 1.733567514 |
| State_Washington | 2001 | 25.98108559 | 1.931790508 |
| State_Washington | 2002 | 26.74225055 | 1.940567962 |
| State_Washington | 2003 | 25.8716126 | 1.89330189 |
| State_Washington | 2004 | 24.90954735 | 1.833247286 |
| State_Washington | 2005 | 24.69291501 | 1.808998062 |
| State_Washington | 2006 | 29.35324469 | 1.933007719 |
| State_Washington | 2007 | 25.30710158 | 1.785097541 |
| State_Washington | 2008 | 25.71413792 | 1.776881432 |
| State_Washington | 2009 | 23.75779971 | 1.703738389 |
| State_Washington | 2010 | 26.01242198 | 1.763560586 |
| State_Washington | 2011 | 22.30265996 | 1.594877146 |
| State_Washington | 2012 | 21.79231333 | 1.576166336 |
| State_Washington | 2013 | 21.58896424 | 1.527268684 |
| State_Washington | 2014 | 25.2423822 | 1.647687491 |
| State_Washington | 2015 | 20.39640209 | 1.453081742 |
| State_Washington | 2016 | 20.88485748 | 1.450613476 |
| State_Washington | 2017 | 18.87047425 | 1.382984028 |
| State_Washington | 2018 | 21.47178658 | 1.448560336 |
| State_Washington | 2019 | 23.94843757 | 1.511415799 |
| State_Washington | 2020 | 23.10824086 | 1.469589277 |
| State_Washington | 2021 | 25.24967905 | 1.582977021 |
| State_Washington | 2022 | 24.13639699 | 1.476536226 |
| State_Washington | 2023 | 20.26009746 | 1.357980159 |
| State_West Virginia | 1999 | 13.20503534 | 2.233543374 |
| State_West Virginia | 2000 | 21.07181674 | 2.817173071 |
| State_West Virginia | 2001 | 19.54963134 | 2.712406795 |
| State_West Virginia | 2002 | 22.12020194 | 2.882182062 |
| State_West Virginia | 2003 | 23.12615301 | 2.916573661 |
| State_West Virginia | 2004 | 20.42474599 | 2.756869213 |
| State_West Virginia | 2005 | 23.02105126 | 2.926099442 |
| State_West Virginia | 2006 | 22.79091869 | 2.872787713 |
| State_West Virginia | 2007 | 24.02989661 | 2.935974323 |
| State_West Virginia | 2008 | 24.24871256 | 2.940690376 |
| State_West Virginia | 2009 | 20.36652417 | 2.675043926 |
| State_West Virginia | 2010 | 21.34969517 | 2.735621607 |
| State_West Virginia | 2011 | 16.8356681 | 2.407860596 |
| State_West Virginia | 2012 | 16.55726749 | 2.369704246 |
| State_West Virginia | 2013 | 13.70820462 | 2.145803372 |
| State_West Virginia | 2014 | 12.95183392 | 2.051653542 |
| State_West Virginia | 2015 | 13.72247349 | 2.125171129 |
| State_West Virginia | 2016 | 11.60401485 | 1.939257646 |
| State_West Virginia | 2017 | 9.826603873 | 1.744507943 |
| State_West Virginia | 2018 | 12.25923692 | 1.967812147 |
| State_West Virginia | 2019 | 12.11529545 | 1.922770706 |
| State_West Virginia | 2020 | 9.102987345 | 1.665938045 |
| State_West Virginia | 2021 | 12.2853544 | 2.024885191 |
| State_West Virginia | 2022 | 13.56058924 | 2.049724985 |
| State_West Virginia | 2023 | 8.28132645 | 1.626199456 |
| State_Wisconsin | 1999 | 13.70673398 | 1.358456534 |
| State_Wisconsin | 2000 | 18.07540413 | 1.56334126 |
| State_Wisconsin | 2001 | 15.81358652 | 1.452150624 |
| State_Wisconsin | 2002 | 14.57922537 | 1.380064926 |
| State_Wisconsin | 2003 | 14.80065078 | 1.377453298 |
| State_Wisconsin | 2004 | 13.58418659 | 1.317142937 |
| State_Wisconsin | 2005 | 13.10855959 | 1.290083792 |
| State_Wisconsin | 2006 | 16.80741516 | 1.431084662 |
| State_Wisconsin | 2007 | 13.75418053 | 1.300896782 |
| State_Wisconsin | 2008 | 13.7506226 | 1.291151273 |
| State_Wisconsin | 2009 | 10.72564208 | 1.114133478 |
| State_Wisconsin | 2010 | 13.14057647 | 1.253945801 |
| State_Wisconsin | 2011 | 10.14282206 | 1.093534889 |
| State_Wisconsin | 2012 | 11.63154147 | 1.160185213 |
| State_Wisconsin | 2013 | 8.860615192 | 1.002791881 |
| State_Wisconsin | 2014 | 11.52890302 | 1.117396638 |
| State_Wisconsin | 2015 | 11.42995134 | 1.108772325 |
| State_Wisconsin | 2016 | 14.89814983 | 1.280367156 |
| State_Wisconsin | 2017 | 12.95670931 | 1.170727749 |
| State_Wisconsin | 2018 | 15.51259105 | 1.277374283 |
| State_Wisconsin | 2019 | 15.28691507 | 1.274226157 |
| State_Wisconsin | 2020 | 16.29948999 | 1.295053153 |
| State_Wisconsin | 2021 | 18.2630329 | 1.430615692 |
| State_Wisconsin | 2022 | 16.72533714 | 1.317038978 |
| State_Wisconsin | 2023 | 14.5758606 | 1.236109299 |
| State_Wyoming | 1999 |  | 6.130956717 |
| State_Wyoming | 2000 |  |  |
| State_Wyoming | 2001 |  |  |
| State_Wyoming | 2002 |  | 5.750988591 |
| State_Wyoming | 2003 |  | 6.455969536 |
| State_Wyoming | 2004 |  | 5.330604673 |
| State_Wyoming | 2005 |  |  |
| State_Wyoming | 2006 |  |  |
| State_Wyoming | 2007 |  |  |
| State_Wyoming | 2008 |  |  |
| State_Wyoming | 2009 |  |  |
| State_Wyoming | 2010 |  |  |
| State_Wyoming | 2011 |  | 5.247819103 |
| State_Wyoming | 2012 |  |  |
| State_Wyoming | 2013 |  |  |
| State_Wyoming | 2014 |  |  |
| State_Wyoming | 2015 |  |  |
| State_Wyoming | 2016 |  | 4.056125092 |
| State_Wyoming | 2017 |  | 5.171525706 |
| State_Wyoming | 2018 | 35.88699808 | 6.576196236 |
| State_Wyoming | 2019 | 25.8544443 | 5.53321562 |
| State_Wyoming | 2020 |  | 4.647609745 |
| State_Wyoming | 2021 | 27.26617692 | 5.831847251 |
| State_Wyoming | 2022 |  | 4.520596877 |
| State_Wyoming | 2023 |  |  |
| Urbanization_Metropolitan | 1999 | 13.54969378 | 0.222216972 |
| Urbanization_Metropolitan | 2000 | 14.61864291 | 0.228475678 |
| Urbanization_Metropolitan | 2001 | 14.39616052 | 0.224873833 |
| Urbanization_Metropolitan | 2002 | 14.84668468 | 0.226634736 |
| Urbanization_Metropolitan | 2003 | 14.82880854 | 0.224296844 |
| Urbanization_Metropolitan | 2004 | 15.1262496 | 0.22517879 |
| Urbanization_Metropolitan | 2005 | 15.25089877 | 0.223299302 |
| Urbanization_Metropolitan | 2006 | 14.98904468 | 0.218858312 |
| Urbanization_Metropolitan | 2007 | 14.40343305 | 0.212522372 |
| Urbanization_Metropolitan | 2008 | 14.04890947 | 0.208073829 |
| Urbanization_Metropolitan | 2009 | 13.21088306 | 0.199401346 |
| Urbanization_Metropolitan | 2010 | 12.94492221 | 0.196002984 |
| Urbanization_Metropolitan | 2011 | 12.48745969 | 0.189314644 |
| Urbanization_Metropolitan | 2012 | 11.62288997 | 0.18108967 |
| Urbanization_Metropolitan | 2013 | 10.72634056 | 0.171814421 |
| Urbanization_Metropolitan | 2014 | 10.42867268 | 0.167773634 |
| Urbanization_Metropolitan | 2015 | 11.1331136 | 0.171114377 |
| Urbanization_Metropolitan | 2016 | 11.86453581 | 0.174585555 |
| Urbanization_Metropolitan | 2017 | 11.77680673 | 0.17209615 |
| Urbanization_Metropolitan | 2018 | 11.82589041 | 0.169932907 |
| Urbanization_Metropolitan | 2019 | 11.83549506 | 0.167986441 |
| Urbanization_Metropolitan | 2020 | 12.69729116 | 0.172087723 |
| Urbanization_Nonmetropolitan | 1999 | 15.5245243 | 0.48035384 |
| Urbanization_Nonmetropolitan | 2000 | 15.82646426 | 0.483099782 |
| Urbanization_Nonmetropolitan | 2001 | 16.38342729 | 0.490383442 |
| Urbanization_Nonmetropolitan | 2002 | 17.91151841 | 0.511462947 |
| Urbanization_Nonmetropolitan | 2003 | 17.62251769 | 0.505061647 |
| Urbanization_Nonmetropolitan | 2004 | 17.61956308 | 0.504172179 |
| Urbanization_Nonmetropolitan | 2005 | 18.44194164 | 0.51311971 |
| Urbanization_Nonmetropolitan | 2006 | 18.79739054 | 0.515687575 |
| Urbanization_Nonmetropolitan | 2007 | 18.09013018 | 0.502198491 |
| Urbanization_Nonmetropolitan | 2008 | 17.35893479 | 0.490075185 |
| Urbanization_Nonmetropolitan | 2009 | 15.89119931 | 0.467536755 |
| Urbanization_Nonmetropolitan | 2010 | 16.48197466 | 0.473297186 |
| Urbanization_Nonmetropolitan | 2011 | 14.22991485 | 0.435463281 |
| Urbanization_Nonmetropolitan | 2012 | 13.88859967 | 0.429259751 |
| Urbanization_Nonmetropolitan | 2013 | 12.28373293 | 0.399120622 |
| Urbanization_Nonmetropolitan | 2014 | 12.9620262 | 0.406772138 |
| Urbanization_Nonmetropolitan | 2015 | 12.76119255 | 0.400479271 |
| Urbanization_Nonmetropolitan | 2016 | 13.57090453 | 0.410886507 |
| Urbanization_Nonmetropolitan | 2017 | 13.96479982 | 0.412328637 |
| Urbanization_Nonmetropolitan | 2018 | 13.64502954 | 0.404322084 |
| Urbanization_Nonmetropolitan | 2019 | 13.89250743 | 0.403780614 |
| Urbanization_Nonmetropolitan | 2020 | 14.77449963 | 0.415152835 |
| Age Groups_65-74 years | 1999 | 1.976229971 | 0.103582596 |
| Age Groups_65-74 years | 2000 | 1.968355585 | 0.103454473 |
| Age Groups_65-74 years | 2001 | 1.963644936 | 0.103349733 |
| Age Groups_65-74 years | 2002 | 2.164392106 | 0.108491173 |
| Age Groups_65-74 years | 2003 | 2.075573019 | 0.105918642 |
| Age Groups_65-74 years | 2004 | 2.0463336 | 0.104699535 |
| Age Groups_65-74 years | 2005 | 1.933088959 | 0.101182501 |
| Age Groups_65-74 years | 2006 | 1.609121312 | 0.091539713 |
| Age Groups_65-74 years | 2007 | 1.79707044 | 0.095513216 |
| Age Groups_65-74 years | 2008 | 1.779994703 | 0.093169181 |
| Age Groups_65-74 years | 2009 | 1.375211409 | 0.080478161 |
| Age Groups_65-74 years | 2010 | 1.395449793 | 0.080166496 |
| Age Groups_65-74 years | 2011 | 1.200974764 | 0.073088997 |
| Age Groups_65-74 years | 2012 | 1.279945727 | 0.073050361 |
| Age Groups_65-74 years | 2013 | 1.082613052 | 0.065522723 |
| Age Groups_65-74 years | 2014 | 1.060674763 | 0.063387441 |
| Age Groups_65-74 years | 2015 | 1.168762096 | 0.065132565 |
| Age Groups_65-74 years | 2016 | 1.365684573 | 0.069065637 |
| Age Groups_65-74 years | 2017 | 1.283543696 | 0.065757936 |
| Age Groups_65-74 years | 2018 | 1.282290266 | 0.064848206 |
| Age Groups_65-74 years | 2019 | 1.349916319 | 0.06548056 |
| Age Groups_65-74 years | 2020 | 1.511548693 | 0.068145878 |
| Age Groups_65-74 years | 2021 | 1.476261507 | 0.066219379 |
| Age Groups_65-74 years | 2022 | 1.479796092 | 0.066178493 |
| Age Groups_65-74 years | 2023 | 1.453065802 | 0.064724695 |
| Age Groups_75-84 years | 1999 | 15.95921247 | 0.361312363 |
| Age Groups_75-84 years | 2000 | 17.3365326 | 0.374499425 |
| Age Groups_75-84 years | 2001 | 16.77833963 | 0.365005352 |
| Age Groups_75-84 years | 2002 | 17.3131496 | 0.368281535 |
| Age Groups_75-84 years | 2003 | 16.64800777 | 0.359290925 |
| Age Groups_75-84 years | 2004 | 17.60598212 | 0.368152009 |
| Age Groups_75-84 years | 2005 | 17.72875796 | 0.368232058 |
| Age Groups_75-84 years | 2006 | 17.97611956 | 0.370503844 |
| Age Groups_75-84 years | 2007 | 17.09272532 | 0.361391823 |
| Age Groups_75-84 years | 2008 | 16.72516779 | 0.357640005 |
| Age Groups_75-84 years | 2009 | 15.18877505 | 0.341514861 |
| Age Groups_75-84 years | 2010 | 15.25902599 | 0.34180087 |
| Age Groups_75-84 years | 2011 | 13.8517506 | 0.324244956 |
| Age Groups_75-84 years | 2012 | 13.74256233 | 0.321777222 |
| Age Groups_75-84 years | 2013 | 12.19646512 | 0.301170239 |
| Age Groups_75-84 years | 2014 | 12.02248973 | 0.296422703 |
| Age Groups_75-84 years | 2015 | 12.15240146 | 0.295434989 |
| Age Groups_75-84 years | 2016 | 12.84993593 | 0.300465152 |
| Age Groups_75-84 years | 2017 | 12.70182247 | 0.293885124 |
| Age Groups_75-84 years | 2018 | 12.75790753 | 0.287878154 |
| Age Groups_75-84 years | 2019 | 12.84293324 | 0.283583785 |
| Age Groups_75-84 years | 2020 | 13.70691765 | 0.288646656 |
| Age Groups_75-84 years | 2021 | 13.88368251 | 0.292693727 |
| Age Groups_75-84 years | 2022 | 12.92768005 | 0.271635478 |
| Age Groups_75-84 years | 2023 | 12.06439622 | 0.25628354 |
| Age Groups_85+ years | 1999 | 58.95496842 | 1.191313377 |
| Age Groups_85+ years | 2000 | 62.83630929 | 1.217428764 |
| Age Groups_85+ years | 2001 | 63.58269716 | 1.214241918 |
| Age Groups_85+ years | 2002 | 66.6314473 | 1.234974937 |
| Age Groups_85+ years | 2003 | 68.47020807 | 1.238177498 |
| Age Groups_85+ years | 2004 | 67.44564257 | 1.218057587 |
| Age Groups_85+ years | 2005 | 69.80164699 | 1.219533469 |
| Age Groups_85+ years | 2006 | 69.13376665 | 1.191961494 |
| Age Groups_85+ years | 2007 | 65.79959103 | 1.142657129 |
| Age Groups_85+ years | 2008 | 63.28139435 | 1.103596045 |
| Age Groups_85+ years | 2009 | 61.76288604 | 1.07271861 |
| Age Groups_85+ years | 2010 | 60.58142513 | 1.050141513 |
| Age Groups_85+ years | 2011 | 59.08833497 | 1.01485011 |
| Age Groups_85+ years | 2012 | 52.84229014 | 0.947396172 |
| Age Groups_85+ years | 2013 | 49.81137398 | 0.908066026 |
| Age Groups_85+ years | 2014 | 49.31655435 | 0.894596873 |
| Age Groups_85+ years | 2015 | 52.86964975 | 0.917013915 |
| Age Groups_85+ years | 2016 | 56.20398064 | 0.938559766 |
| Age Groups_85+ years | 2017 | 56.64214132 | 0.935754514 |
| Age Groups_85+ years | 2018 | 56.4748767 | 0.928943521 |
| Age Groups_85+ years | 2019 | 56.48786866 | 0.924788806 |
| Age Groups_85+ years | 2020 | 60.26955344 | 0.95140054 |
| Age Groups_85+ years | 2021 | 62.31847485 | 1.02120276 |
| Age Groups_85+ years | 2022 | 60.96331285 | 0.969505388 |
| Age Groups_85+ years | 2023 | 56.82019958 | 0.957704269 |

Supplementary table 3. Characteristics with APC, 95% CI, and Time Range.

| Metric | Measure | Start | End | APC | Lower | Upper | P value |
| --- | --- | --- | --- | --- | --- | --- | --- |
| Census Region | Midwest | 1999 | 2006 | 1.5094 | 0.6336 | 2.3928 | 0.002339 |
| Census Region | Midwest | 2006 | 2013 | -5.7696 | -6.8206 | -4.7068 | 0 |
| Census Region | Midwest | 2013 | 2021 | 2.5589 | 1.641 | 3.485 | 0.000031 |
| Census Region | Midwest | 2021 | 2023 | -5.7398 | -11.6765 | 0.596 | 0.071638 |
| Census Region | Northeast | 1999 | 2003 | 1.595 | -1.7916 | 5.0983 | 0.338543 |
| Census Region | Northeast | 2003 | 2014 | -3.9477 | -4.7759 | -3.1123 | 0 |
| Census Region | Northeast | 2014 | 2023 | 0.2857 | -0.7705 | 1.3531 | 0.577163 |
| Census Region | South | 1999 | 2006 | 1.6899 | 0.4874 | 2.9067 | 0.009152 |
| Census Region | South | 2006 | 2014 | -5.4411 | -6.5625 | -4.3062 | 0 |
| Census Region | South | 2014 | 2021 | 3.6386 | 2.1927 | 5.105 | 0.000085 |
| Census Region | South | 2021 | 2023 | -4.2826 | -11.4377 | 3.4506 | 0.246952 |
| Census Region | West | 1999 | 2006 | 3.4969 | 1.3967 | 5.6406 | 0.002531 |
| Census Region | West | 2006 | 2013 | -3.3199 | -5.5323 | -1.0557 | 0.006832 |
| Census Region | West | 2013 | 2023 | 0.9233 | -0.0879 | 1.9448 | 0.071059 |
| Race | Hispanic | 1999 | 2004 | 10.7538 | 3.2425 | 18.8116 | 0.006959 |
| Race | Hispanic | 2004 | 2017 | -1.0404 | -2.2673 | 0.2019 | 0.094878 |
| Race | Hispanic | 2017 | 2023 | 4.0092 | 1.2479 | 6.8458 | 0.006756 |
| Race | NH Black | 1999 | 2005 | 2.7162 | 0.251 | 5.242 | 0.032937 |
| Race | NH Black | 2005 | 2014 | -5.5317 | -7.0027 | -4.0374 | 0.000002 |
| Race | NH Black | 2014 | 2021 | 2.5464 | 0.2117 | 4.9356 | 0.034505 |
| Race | NH Black | 2021 | 2023 | -11.006 | -22.4265 | 2.0958 | 0.090061 |
| Race | NH Other | 1999 | 2004 | 9.1498 | -1.4745 | 20.9198 | 0.08971 |
| Race | NH Other | 2004 | 2023 | -1.3742 | -2.1547 | -0.5874 | 0.001657 |
| Race | NH White | 1999 | 2006 | 1.4799 | 0.5486 | 2.4199 | 0.004166 |
| Race | NH White | 2006 | 2014 | -4.4207 | -5.3093 | -3.5238 | 0 |
| Race | NH White | 2014 | 2021 | 3.2086 | 2.0364 | 4.3943 | 0.000037 |
| Race | NH White | 2021 | 2023 | -4.7714 | -10.7932 | 1.657 | 0.130765 |
| Sex | Both | 1999 | 2006 | 1.451 | 0.5258 | 2.3848 | 0.004556 |
| Sex | Both | 2006 | 2014 | -4.5411 | -5.4096 | -3.6647 | 0 |
| Sex | Both | 2014 | 2021 | 2.7642 | 1.6379 | 3.903 | 0.000111 |
| Sex | Both | 2021 | 2023 | -4.659 | -10.4389 | 1.494 | 0.12407 |
| Sex | Female | 1999 | 2006 | 1.6891 | 0.7949 | 2.5913 | 0.001154 |
| Sex | Female | 2006 | 2014 | -4.3347 | -5.1816 | -3.4802 | 0 |
| Sex | Female | 2014 | 2021 | 3.1124 | 2.0083 | 4.2284 | 0.000027 |
| Sex | Female | 2021 | 2023 | -5.3337 | -10.9543 | 0.6417 | 0.075381 |
| Sex | Male | 1999 | 2006 | 0.9606 | -0.339 | 2.2772 | 0.13582 |
| Sex | Male | 2006 | 2014 | -5.0663 | -6.263 | -3.8544 | 0 |
| Sex | Male | 2014 | 2021 | 2.2677 | 0.7283 | 3.8306 | 0.006802 |
| Sex | Male | 2021 | 2023 | -4.4534 | -12.3348 | 4.1365 | 0.275443 |
| State | Alabama | 1999 | 2005 | 8.312 | 2.3265 | 14.6476 | 0.008708 |
| State | Alabama | 2005 | 2012 | -11.1232 | -16.2266 | -5.7089 | 0.000593 |
| State | Alabama | 2012 | 2023 | -0.0903 | -2.712 | 2.6021 | 0.943692 |
| State | Arizona | 1999 | 2023 | 0.0444 | -0.9998 | 1.0996 | 0.931009 |
| State | Arkansas | 1999 | 2006 | 13.928 | 6.3486 | 22.0476 | 0.000935 |
| State | Arkansas | 2006 | 2015 | -6.5511 | -11.0061 | -1.873 | 0.009419 |
| State | Arkansas | 2015 | 2023 | 4.3984 | -0.5297 | 9.5706 | 0.077634 |
| State | California | 1999 | 2005 | 6.1046 | 3.1567 | 9.1368 | 0.000285 |
| State | California | 2005 | 2023 | -1.991 | -2.4375 | -1.5425 | 0 |
| State | Colorado | 1999 | 2008 | 2.1411 | -1.8423 | 6.286 | 0.272521 |
| State | Colorado | 2008 | 2013 | -11.6935 | -23.073 | 1.3693 | 0.073676 |
| State | Colorado | 2013 | 2016 | 39.5576 | -7.3644 | 110.2466 | 0.102988 |
| State | Colorado | 2016 | 2023 | -1.7303 | -5.3688 | 2.0481 | 0.337923 |
| State | Connecticut | 1999 | 2001 | 16.6598 | -19.3741 | 68.7983 | 0.3946 |
| State | Connecticut | 2001 | 2023 | -4.0739 | -4.9523 | -3.1873 | 0 |
| State | Florida | 1999 | 2001 | -9.3333 | -23.518 | 7.4822 | 0.231139 |
| State | Florida | 2001 | 2005 | 6.8233 | -1.7428 | 16.1363 | 0.110081 |
| State | Florida | 2005 | 2012 | -9.0119 | -11.6998 | -6.2422 | 0.000025 |
| State | Florida | 2012 | 2021 | 4.9627 | 3.0862 | 6.8733 | 0.000102 |
| State | Florida | 2021 | 2023 | -6.1573 | -18.6613 | 8.2689 | 0.349021 |
| State | Georgia | 1999 | 2014 | -3.7191 | -5.205 | -2.21 | 0.000057 |
| State | Georgia | 2014 | 2023 | 3.6348 | 0.5429 | 6.8217 | 0.023178 |
| State | Illinois | 1999 | 2006 | 0.5481 | -2.1137 | 3.2822 | 0.672709 |
| State | Illinois | 2006 | 2014 | -7.2049 | -9.9295 | -4.3979 | 0.00006 |
| State | Illinois | 2014 | 2023 | -0.0545 | -2.243 | 2.183 | 0.959159 |
| State | Indiana | 1999 | 2021 | -0.9037 | -1.585 | -0.2177 | 0.012481 |
| State | Indiana | 2021 | 2023 | -17.2685 | -40.3136 | 14.6744 | 0.239955 |
| State | Iowa | 1999 | 2015 | -1.8672 | -2.7584 | -0.9679 | 0.000341 |
| State | Iowa | 2015 | 2023 | 2.8437 | 0.2932 | 5.4591 | 0.030449 |
| State | Kansas | 1999 | 2004 | 3.4959 | -2.1169 | 9.4306 | 0.207439 |
| State | Kansas | 2004 | 2015 | -7.2815 | -9.2974 | -5.2207 | 0.000003 |
| State | Kansas | 2015 | 2018 | 16.9102 | -12.3217 | 55.8879 | 0.263641 |
| State | Kansas | 2018 | 2023 | -4.0418 | -9.7529 | 2.0308 | 0.171278 |
| State | Kentucky | 1999 | 2023 | -0.9049 | -1.6199 | -0.1848 | 0.016121 |
| State | Louisiana | 1999 | 2021 | 1.3867 | 0.5001 | 2.2811 | 0.003826 |
| State | Louisiana | 2021 | 2023 | -17.1039 | -41.8262 | 18.1248 | 0.282336 |
| State | Maryland | 1999 | 2015 | -6.73 | -8.0814 | -5.3587 | 0 |
| State | Maryland | 2015 | 2023 | 5.8929 | 1.4774 | 10.5005 | 0.010954 |
| State | Massachusetts | 1999 | 2017 | -4.7013 | -5.6727 | -3.7199 | 0 |
| State | Massachusetts | 2017 | 2023 | 6.3261 | 0.5378 | 12.4476 | 0.033309 |
| State | Michigan | 1999 | 2015 | -2.7815 | -3.754 | -1.7992 | 0.00001 |
| State | Michigan | 2015 | 2023 | 3.1206 | 0.3125 | 6.0075 | 0.030925 |
| State | Minnesota | 1999 | 2013 | -1.1381 | -2.7231 | 0.4727 | 0.153459 |
| State | Minnesota | 2013 | 2021 | 7.9667 | 4.1239 | 11.9514 | 0.000342 |
| State | Minnesota | 2021 | 2023 | -10.5429 | -30.0664 | 14.431 | 0.353113 |
| State | Mississippi | 1999 | 2023 | 0.6381 | -0.3747 | 1.6612 | 0.206186 |
| State | Missouri | 1999 | 2005 | 3.4655 | -3.5599 | 11.0028 | 0.324258 |
| State | Missouri | 2005 | 2023 | -3.1235 | -4.4032 | -1.8266 | 0.000072 |
| State | Nebraska | 1999 | 2023 | 1.1347 | 0.022 | 2.2599 | 0.045961 |
| State | New Hampshire | 1999 | 2023 | -3.4494 | -4.4696 | -2.4182 | 0.000001 |
| State | New Jersey | 1999 | 2011 | 0.5671 | -1.161 | 2.3254 | 0.50393 |
| State | New Jersey | 2011 | 2023 | -3.7139 | -5.3865 | -2.0116 | 0.000216 |
| State | New York | 1999 | 2014 | -3.4636 | -4.5314 | -2.3838 | 0.000002 |
| State | New York | 2014 | 2023 | 2.5432 | 0.1153 | 5.0301 | 0.040853 |
| State | North Carolina | 1999 | 2001 | 16.6532 | -8.0228 | 47.9493 | 0.186211 |
| State | North Carolina | 2001 | 2009 | -2.7439 | -5.4054 | -0.0075 | 0.049461 |
| State | North Carolina | 2009 | 2012 | -12.8212 | -30.6341 | 9.5659 | 0.218788 |
| State | North Carolina | 2012 | 2023 | 1.3627 | -0.1245 | 2.872 | 0.069706 |
| State | Ohio | 1999 | 2006 | 2.2536 | -0.1697 | 4.7358 | 0.066544 |
| State | Ohio | 2006 | 2013 | -7.4324 | -10.2948 | -4.4786 | 0.000074 |
| State | Ohio | 2013 | 2023 | -0.9479 | -2.5939 | 0.7259 | 0.246909 |
| State | Oklahoma | 1999 | 2001 | 27.3129 | -15.5699 | 91.9764 | 0.231665 |
| State | Oklahoma | 2001 | 2011 | -3.5962 | -6.7642 | -0.3206 | 0.033534 |
| State | Oklahoma | 2011 | 2023 | 5.4602 | 3.4621 | 7.4969 | 0.000019 |
| State | Oregon | 1999 | 2014 | -0.0717 | -1.3745 | 1.2484 | 0.910397 |
| State | Oregon | 2014 | 2023 | 6.7139 | 4.4921 | 8.9829 | 0.000003 |
| State | Pennsylvania | 1999 | 2005 | 2.5213 | 0.0815 | 5.0205 | 0.043502 |
| State | Pennsylvania | 2005 | 2013 | -6.2561 | -8.133 | -4.3408 | 0.000003 |
| State | Pennsylvania | 2013 | 2023 | 0.3193 | -0.9575 | 1.6126 | 0.606305 |
| State | South Carolina | 1999 | 2023 | -0.6015 | -1.4823 | 0.2871 | 0.174166 |
| State | Tennessee | 1999 | 2008 | 4.9368 | 3.0604 | 6.8474 | 0.00003 |
| State | Tennessee | 2008 | 2012 | -10.4963 | -18.3344 | -1.9059 | 0.020591 |
| State | Tennessee | 2012 | 2023 | -2.1821 | -3.5406 | -0.8044 | 0.00398 |
| State | Texas | 1999 | 2008 | -0.0478 | -1.2541 | 1.1732 | 0.933867 |
| State | Texas | 2008 | 2014 | -4.4216 | -7.1464 | -1.6168 | 0.00473 |
| State | Texas | 2014 | 2017 | 8.0572 | -3.9965 | 21.6243 | 0.181766 |
| State | Texas | 2017 | 2023 | -0.0232 | -1.8543 | 1.842 | 0.978865 |
| State | Virginia | 1999 | 2010 | -1.3512 | -3.6996 | 1.0544 | 0.2499 |
| State | Virginia | 2010 | 2013 | -17.0003 | -42.6093 | 20.036 | 0.301542 |
| State | Virginia | 2013 | 2023 | 3.7432 | 0.8837 | 6.6836 | 0.012997 |
| State | Washington | 1999 | 2001 | 17.2249 | -16.3716 | 64.3183 | 0.338005 |
| State | Washington | 2001 | 2023 | -0.8415 | -1.471 | -0.2079 | 0.011877 |
| State | West Virginia | 1999 | 2006 | 4.4 | -1.2922 | 10.4203 | 0.124819 |
| State | West Virginia | 2006 | 2023 | -5.5303 | -6.9737 | -4.0644 | 0 |
| State | Wisconsin | 1999 | 2013 | -2.7229 | -4.318 | -1.1012 | 0.002345 |
| State | Wisconsin | 2013 | 2023 | 4.7654 | 2.1873 | 7.4085 | 0.000894 |
| Urbanization | Metropolitan | 1999 | 2005 | 1.9111 | 0.6937 | 3.1433 | 0.004501 |
| Urbanization | Metropolitan | 2005 | 2014 | -4.0078 | -4.7336 | -3.2765 | 0 |
| Urbanization | Metropolitan | 2014 | 2020 | 2.5747 | 1.4369 | 3.7252 | 0.00024 |
| Urbanization | Nonmetropolitan | 1999 | 2006 | 3.0361 | 1.8073 | 4.2797 | 0.000103 |
| Urbanization | Nonmetropolitan | 2006 | 2013 | -5.6373 | -7.0543 | -4.1988 | 0.000001 |
| Urbanization | Nonmetropolitan | 2013 | 2020 | 1.707 | 0.4753 | 2.9537 | 0.009946 |
| Age Groups | 65-74 years | 1999 | 2004 | 1.0441 | -3.2715 | 5.5522 | 0.622075 |
| Age Groups | 65-74 years | 2004 | 2013 | -6.5868 | -8.7167 | -4.4073 | 0.000009 |
| Age Groups | 65-74 years | 2013 | 2023 | 3.1761 | 1.6608 | 4.7139 | 0.000345 |
| Age Groups | 75-84 years | 1999 | 2006 | 1.3936 | 0.2957 | 2.5035 | 0.016384 |
| Age Groups | 75-84 years | 2006 | 2014 | -4.9494 | -6.0342 | -3.8521 | 0 |
| Age Groups | 75-84 years | 2014 | 2021 | 1.932 | 0.467 | 3.4185 | 0.013236 |
| Age Groups | 75-84 years | 2021 | 2023 | -6.2771 | -13.4706 | 1.5145 | 0.10359 |
| Age Groups | 85+ years | 1999 | 2005 | 2.8174 | 1.3182 | 4.3387 | 0.001177 |
| Age Groups | 85+ years | 2005 | 2014 | -3.7216 | -4.5657 | -2.87 | 0 |
| Age Groups | 85+ years | 2014 | 2021 | 3.0035 | 1.6952 | 4.3287 | 0.000207 |
| Age Groups | 85+ years | 2021 | 2023 | -3.9593 | -10.8928 | 3.5137 | 0.266903 |
| Census Region | Midwest | 1999 | 2006 | 1.5094 | 0.6336 | 2.3928 | 0.002339 |
| Census Region | Midwest | 2006 | 2013 | -5.7696 | -6.8206 | -4.7068 | 0 |
| Census Region | Midwest | 2013 | 2021 | 2.5589 | 1.641 | 3.485 | 0.000031 |
| Census Region | Midwest | 2021 | 2023 | -5.7398 | -11.6765 | 0.596 | 0.071638 |
| Census Region | Northeast | 1999 | 2003 | 1.595 | -1.7916 | 5.0983 | 0.338543 |
| Census Region | Northeast | 2003 | 2014 | -3.9477 | -4.7759 | -3.1123 | 0 |
| Census Region | Northeast | 2014 | 2023 | 0.2857 | -0.7705 | 1.3531 | 0.577163 |
| Census Region | South | 1999 | 2006 | 1.6899 | 0.4874 | 2.9067 | 0.009152 |
| Census Region | South | 2006 | 2014 | -5.4411 | -6.5625 | -4.3062 | 0 |
| Census Region | South | 2014 | 2021 | 3.6386 | 2.1927 | 5.105 | 0.000085 |
| Census Region | South | 2021 | 2023 | -4.2826 | -11.4377 | 3.4506 | 0.246952 |
| Census Region | West | 1999 | 2006 | 3.4969 | 1.3967 | 5.6406 | 0.002531 |
| Census Region | West | 2006 | 2013 | -3.3199 | -5.5323 | -1.0557 | 0.006832 |
| Census Region | West | 2013 | 2023 | 0.9233 | -0.0879 | 1.9448 | 0.071059 |
| Race | Hispanic | 1999 | 2004 | 10.7538 | 3.2425 | 18.8116 | 0.006959 |
| Race | Hispanic | 2004 | 2017 | -1.0404 | -2.2673 | 0.2019 | 0.094878 |
| Race | Hispanic | 2017 | 2023 | 4.0092 | 1.2479 | 6.8458 | 0.006756 |
| Race | NH Black | 1999 | 2005 | 2.7162 | 0.251 | 5.242 | 0.032937 |
| Race | NH Black | 2005 | 2014 | -5.5317 | -7.0027 | -4.0374 | 0.000002 |
| Race | NH Black | 2014 | 2021 | 2.5464 | 0.2117 | 4.9356 | 0.034505 |
| Race | NH Black | 2021 | 2023 | -11.006 | -22.4265 | 2.0958 | 0.090061 |
| Race | NH Other | 1999 | 2004 | 9.1498 | -1.4745 | 20.9198 | 0.08971 |
| Race | NH Other | 2004 | 2023 | -1.3742 | -2.1547 | -0.5874 | 0.001657 |
| Race | NH White | 1999 | 2006 | 1.4799 | 0.5486 | 2.4199 | 0.004166 |
| Race | NH White | 2006 | 2014 | -4.4207 | -5.3093 | -3.5238 | 0 |
| Race | NH White | 2014 | 2021 | 3.2086 | 2.0364 | 4.3943 | 0.000037 |
| Race | NH White | 2021 | 2023 | -4.7714 | -10.7932 | 1.657 | 0.130765 |
| Sex | Both | 1999 | 2006 | 1.451 | 0.5258 | 2.3848 | 0.004556 |
| Sex | Both | 2006 | 2014 | -4.5411 | -5.4096 | -3.6647 | 0 |
| Sex | Both | 2014 | 2021 | 2.7642 | 1.6379 | 3.903 | 0.000111 |
| Sex | Both | 2021 | 2023 | -4.659 | -10.4389 | 1.494 | 0.12407 |
| Sex | Female | 1999 | 2006 | 1.6891 | 0.7949 | 2.5913 | 0.001154 |
| Sex | Female | 2006 | 2014 | -4.3347 | -5.1816 | -3.4802 | 0 |
| Sex | Female | 2014 | 2021 | 3.1124 | 2.0083 | 4.2284 | 0.000027 |
| Sex | Female | 2021 | 2023 | -5.3337 | -10.9543 | 0.6417 | 0.075381 |
| Sex | Male | 1999 | 2006 | 0.9606 | -0.339 | 2.2772 | 0.13582 |
| Sex | Male | 2006 | 2014 | -5.0663 | -6.263 | -3.8544 | 0 |
| Sex | Male | 2014 | 2021 | 2.2677 | 0.7283 | 3.8306 | 0.006802 |
| Sex | Male | 2021 | 2023 | -4.4534 | -12.3348 | 4.1365 | 0.275443 |
| State | Alabama | 1999 | 2005 | 8.312 | 2.3265 | 14.6476 | 0.008708 |
| State | Alabama | 2005 | 2012 | -11.1232 | -16.2266 | -5.7089 | 0.000593 |
| State | Alabama | 2012 | 2023 | -0.0903 | -2.712 | 2.6021 | 0.943692 |
| State | Arizona | 1999 | 2023 | 0.0444 | -0.9998 | 1.0996 | 0.931009 |
| State | Arkansas | 1999 | 2006 | 13.928 | 6.3486 | 22.0476 | 0.000935 |
| State | Arkansas | 2006 | 2015 | -6.5511 | -11.0061 | -1.873 | 0.009419 |
| State | Arkansas | 2015 | 2023 | 4.3984 | -0.5297 | 9.5706 | 0.077634 |
| State | California | 1999 | 2005 | 6.1046 | 3.1567 | 9.1368 | 0.000285 |
| State | California | 2005 | 2023 | -1.991 | -2.4375 | -1.5425 | 0 |
| State | Colorado | 1999 | 2008 | 2.1411 | -1.8423 | 6.286 | 0.272521 |
| State | Colorado | 2008 | 2013 | -11.6935 | -23.073 | 1.3693 | 0.073676 |
| State | Colorado | 2013 | 2016 | 39.5576 | -7.3644 | 110.2466 | 0.102988 |
| State | Colorado | 2016 | 2023 | -1.7303 | -5.3688 | 2.0481 | 0.337923 |
| State | Connecticut | 1999 | 2001 | 16.6598 | -19.3741 | 68.7983 | 0.3946 |
| State | Connecticut | 2001 | 2023 | -4.0739 | -4.9523 | -3.1873 | 0 |
| State | Florida | 1999 | 2001 | -9.3333 | -23.518 | 7.4822 | 0.231139 |
| State | Florida | 2001 | 2005 | 6.8233 | -1.7428 | 16.1363 | 0.110081 |
| State | Florida | 2005 | 2012 | -9.0119 | -11.6998 | -6.2422 | 0.000025 |
| State | Florida | 2012 | 2021 | 4.9627 | 3.0862 | 6.8733 | 0.000102 |
| State | Florida | 2021 | 2023 | -6.1573 | -18.6613 | 8.2689 | 0.349021 |
| State | Georgia | 1999 | 2014 | -3.7191 | -5.205 | -2.21 | 0.000057 |
| State | Georgia | 2014 | 2023 | 3.6348 | 0.5429 | 6.8217 | 0.023178 |
| State | Illinois | 1999 | 2006 | 0.5481 | -2.1137 | 3.2822 | 0.672709 |
| State | Illinois | 2006 | 2014 | -7.2049 | -9.9295 | -4.3979 | 0.00006 |
| State | Illinois | 2014 | 2023 | -0.0545 | -2.243 | 2.183 | 0.959159 |
| State | Indiana | 1999 | 2021 | -0.9037 | -1.585 | -0.2177 | 0.012481 |
| State | Indiana | 2021 | 2023 | -17.2685 | -40.3136 | 14.6744 | 0.239955 |
| State | Iowa | 1999 | 2015 | -1.8672 | -2.7584 | -0.9679 | 0.000341 |
| State | Iowa | 2015 | 2023 | 2.8437 | 0.2932 | 5.4591 | 0.030449 |
| State | Kansas | 1999 | 2004 | 3.4959 | -2.1169 | 9.4306 | 0.207439 |
| State | Kansas | 2004 | 2015 | -7.2815 | -9.2974 | -5.2207 | 0.000003 |
| State | Kansas | 2015 | 2018 | 16.9102 | -12.3217 | 55.8879 | 0.263641 |
| State | Kansas | 2018 | 2023 | -4.0418 | -9.7529 | 2.0308 | 0.171278 |
| State | Kentucky | 1999 | 2023 | -0.9049 | -1.6199 | -0.1848 | 0.016121 |
| State | Louisiana | 1999 | 2021 | 1.3867 | 0.5001 | 2.2811 | 0.003826 |
| State | Louisiana | 2021 | 2023 | -17.1039 | -41.8262 | 18.1248 | 0.282336 |
| State | Maryland | 1999 | 2015 | -6.73 | -8.0814 | -5.3587 | 0 |
| State | Maryland | 2015 | 2023 | 5.8929 | 1.4774 | 10.5005 | 0.010954 |
| State | Massachusetts | 1999 | 2017 | -4.7013 | -5.6727 | -3.7199 | 0 |
| State | Massachusetts | 2017 | 2023 | 6.3261 | 0.5378 | 12.4476 | 0.033309 |
| State | Michigan | 1999 | 2015 | -2.7815 | -3.754 | -1.7992 | 0.00001 |
| State | Michigan | 2015 | 2023 | 3.1206 | 0.3125 | 6.0075 | 0.030925 |
| State | Minnesota | 1999 | 2013 | -1.1381 | -2.7231 | 0.4727 | 0.153459 |
| State | Minnesota | 2013 | 2021 | 7.9667 | 4.1239 | 11.9514 | 0.000342 |
| State | Minnesota | 2021 | 2023 | -10.5429 | -30.0664 | 14.431 | 0.353113 |
| State | Mississippi | 1999 | 2023 | 0.6381 | -0.3747 | 1.6612 | 0.206186 |
| State | Missouri | 1999 | 2005 | 3.4655 | -3.5599 | 11.0028 | 0.324258 |
| State | Missouri | 2005 | 2023 | -3.1235 | -4.4032 | -1.8266 | 0.000072 |
| State | Nebraska | 1999 | 2023 | 1.1347 | 0.022 | 2.2599 | 0.045961 |
| State | New Hampshire | 1999 | 2023 | -3.4494 | -4.4696 | -2.4182 | 0.000001 |
| State | New Jersey | 1999 | 2011 | 0.5671 | -1.161 | 2.3254 | 0.50393 |
| State | New Jersey | 2011 | 2023 | -3.7139 | -5.3865 | -2.0116 | 0.000216 |
| State | New York | 1999 | 2014 | -3.4636 | -4.5314 | -2.3838 | 0.000002 |
| State | New York | 2014 | 2023 | 2.5432 | 0.1153 | 5.0301 | 0.040853 |
| State | North Carolina | 1999 | 2001 | 16.6532 | -8.0228 | 47.9493 | 0.186211 |
| State | North Carolina | 2001 | 2009 | -2.7439 | -5.4054 | -0.0075 | 0.049461 |
| State | North Carolina | 2009 | 2012 | -12.8212 | -30.6341 | 9.5659 | 0.218788 |
| State | North Carolina | 2012 | 2023 | 1.3627 | -0.1245 | 2.872 | 0.069706 |
| State | Ohio | 1999 | 2006 | 2.2536 | -0.1697 | 4.7358 | 0.066544 |
| State | Ohio | 2006 | 2013 | -7.4324 | -10.2948 | -4.4786 | 0.000074 |
| State | Ohio | 2013 | 2023 | -0.9479 | -2.5939 | 0.7259 | 0.246909 |
| State | Oklahoma | 1999 | 2001 | 27.3129 | -15.5699 | 91.9764 | 0.231665 |
| State | Oklahoma | 2001 | 2011 | -3.5962 | -6.7642 | -0.3206 | 0.033534 |
| State | Oklahoma | 2011 | 2023 | 5.4602 | 3.4621 | 7.4969 | 0.000019 |
| State | Oregon | 1999 | 2014 | -0.0717 | -1.3745 | 1.2484 | 0.910397 |
| State | Oregon | 2014 | 2023 | 6.7139 | 4.4921 | 8.9829 | 0.000003 |
| State | Pennsylvania | 1999 | 2005 | 2.5213 | 0.0815 | 5.0205 | 0.043502 |
| State | Pennsylvania | 2005 | 2013 | -6.2561 | -8.133 | -4.3408 | 0.000003 |
| State | Pennsylvania | 2013 | 2023 | 0.3193 | -0.9575 | 1.6126 | 0.606305 |
| State | South Carolina | 1999 | 2023 | -0.6015 | -1.4823 | 0.2871 | 0.174166 |
| State | Tennessee | 1999 | 2008 | 4.9368 | 3.0604 | 6.8474 | 0.00003 |
| State | Tennessee | 2008 | 2012 | -10.4963 | -18.3344 | -1.9059 | 0.020591 |
| State | Tennessee | 2012 | 2023 | -2.1821 | -3.5406 | -0.8044 | 0.00398 |
| State | Texas | 1999 | 2008 | -0.0478 | -1.2541 | 1.1732 | 0.933867 |
| State | Texas | 2008 | 2014 | -4.4216 | -7.1464 | -1.6168 | 0.00473 |
| State | Texas | 2014 | 2017 | 8.0572 | -3.9965 | 21.6243 | 0.181766 |
| State | Texas | 2017 | 2023 | -0.0232 | -1.8543 | 1.842 | 0.978865 |
| State | Virginia | 1999 | 2010 | -1.3512 | -3.6996 | 1.0544 | 0.2499 |
| State | Virginia | 2010 | 2013 | -17.0003 | -42.6093 | 20.036 | 0.301542 |
| State | Virginia | 2013 | 2023 | 3.7432 | 0.8837 | 6.6836 | 0.012997 |
| State | Washington | 1999 | 2001 | 17.2249 | -16.3716 | 64.3183 | 0.338005 |
| State | Washington | 2001 | 2023 | -0.8415 | -1.471 | -0.2079 | 0.011877 |
| State | West Virginia | 1999 | 2006 | 4.4 | -1.2922 | 10.4203 | 0.124819 |
| State | West Virginia | 2006 | 2023 | -5.5303 | -6.9737 | -4.0644 | 0 |
| State | Wisconsin | 1999 | 2013 | -2.7229 | -4.318 | -1.1012 | 0.002345 |
| State | Wisconsin | 2013 | 2023 | 4.7654 | 2.1873 | 7.4085 | 0.000894 |
| Urbanization | Metropolitan | 1999 | 2005 | 1.9111 | 0.6937 | 3.1433 | 0.004501 |
| Urbanization | Metropolitan | 2005 | 2014 | -4.0078 | -4.7336 | -3.2765 | 0 |
| Urbanization | Metropolitan | 2014 | 2020 | 2.5747 | 1.4369 | 3.7252 | 0.00024 |
| Urbanization | Nonmetropolitan | 1999 | 2006 | 3.0361 | 1.8073 | 4.2797 | 0.000103 |
| Urbanization | Nonmetropolitan | 2006 | 2013 | -5.6373 | -7.0543 | -4.1988 | 0.000001 |
| Urbanization | Nonmetropolitan | 2013 | 2020 | 1.707 | 0.4753 | 2.9537 | 0.009946 |
| Age Groups | 65-74 years | 1999 | 2004 | 1.0441 | -3.2715 | 5.5522 | 0.622075 |
| Age Groups | 65-74 years | 2004 | 2013 | -6.5868 | -8.7167 | -4.4073 | 0.000009 |
| Age Groups | 65-74 years | 2013 | 2023 | 3.1761 | 1.6608 | 4.7139 | 0.000345 |
| Age Groups | 75-84 years | 1999 | 2006 | 1.3936 | 0.2957 | 2.5035 | 0.016384 |
| Age Groups | 75-84 years | 2006 | 2014 | -4.9494 | -6.0342 | -3.8521 | 0 |
| Age Groups | 75-84 years | 2014 | 2021 | 1.932 | 0.467 | 3.4185 | 0.013236 |
| Age Groups | 75-84 years | 2021 | 2023 | -6.2771 | -13.4706 | 1.5145 | 0.10359 |
| Age Groups | 85+ years | 1999 | 2005 | 2.8174 | 1.3182 | 4.3387 | 0.001177 |
| Age Groups | 85+ years | 2005 | 2014 | -3.7216 | -4.5657 | -2.87 | 0 |
| Age Groups | 85+ years | 2014 | 2021 | 3.0035 | 1.6952 | 4.3287 | 0.000207 |
| Age Groups | 85+ years | 2021 | 2023 | -3.9593 | -10.8928 | 3.5137 | 0.266903 |

Supplementary table 4. Characteristics with AAPC, 95% CI, and Time Range.

| Metric | Measure | AAPC | Lower | Upper | *P* value |
| --- | --- | --- | --- | --- | --- |
| Census Region | Midwest | -0.9423 | -1.6141 | -0.2659 | 0.006397 |
| Census Region | Northeast | -1.4645 | -2.1909 | -0.7327 | 0.000093 |
| Census Region | South | -0.6961 | -1.5368 | 0.1518 | 0.107353 |
| Census Region | West | 0.4015 | -0.522 | 1.3336 | 0.395393 |
| Race | Hispanic | 2.5768 | 0.9261 | 4.2545 | 0.002115 |
| Race | NH Black | -1.6878 | -3.0915 | -0.2638 | 0.020339 |
| Race | NH Other | 0.7312 | -1.3521 | 2.8585 | 0.494448 |
| Race | NH White | -0.5633 | -1.2509 | 0.1291 | 0.110569 |
| Sex | Both | -0.7286 | -1.3925 | -0.0603 | 0.032673 |
| Sex | Female | -0.5499 | -1.1987 | 0.1031 | 0.09868 |
| Sex | Male | -1.1729 | -2.0861 | -0.2512 | 0.012739 |
| State | Alabama | -1.4736 | -3.7762 | 0.8841 | 0.218543 |
| State | Arizona | 0.0444 | -0.9998 | 1.0996 | 0.931009 |
| State | Arkansas | 2.7339 | -0.238 | 5.7944 | 0.071727 |
| State | California | -0.0269 | -0.76 | 0.7115 | 0.942812 |
| State | Colorado | 1.8783 | -3.6975 | 7.7771 | 0.516982 |
| State | Connecticut | -2.4968 | -5.3778 | 0.4719 | 0.098472 |
| State | Florida | -1.1773 | -3.4113 | 1.1085 | 0.310072 |
| State | Georgia | -1.0246 | -2.4052 | 0.3754 | 0.150694 |
| State | Illinois | -2.3261 | -3.6891 | -0.9438 | 0.001029 |
| State | Indiana | -2.3831 | -4.9118 | 0.2129 | 0.071677 |
| State | Iowa | -0.3214 | -1.2858 | 0.6524 | 0.516361 |
| State | Kansas | -1.6422 | -5.2723 | 2.1271 | 0.388148 |
| State | Kentucky | -0.9049 | -1.6199 | -0.1848 | 0.016121 |
| State | Louisiana | -0.3004 | -3.1253 | 2.6069 | 0.837477 |
| State | Maryland | -2.6991 | -4.26 | -1.1128 | 0.000913 |
| State | Massachusetts | -2.0566 | -3.5148 | -0.5763 | 0.006626 |
| State | Michigan | -0.8526 | -1.9076 | 0.2137 | 0.116667 |
| State | Minnesota | 0.9636 | -1.41 | 3.3943 | 0.429491 |
| State | Mississippi | 0.6381 | -0.3747 | 1.6612 | 0.206186 |
| State | Missouri | -1.5166 | -3.3692 | 0.3715 | 0.114734 |
| State | Nebraska | 1.1347 | 0.022 | 2.2599 | 0.045961 |
| State | New Hampshire | -3.4494 | -4.4696 | -2.4182 | 0.000001 |
| State | New Jersey | -1.5966 | -2.7295 | -0.4505 | 0.006444 |
| State | New York | -1.2534 | -2.3019 | -0.1936 | 0.020566 |
| State | North Carolina | -0.7358 | -4.0014 | 2.6408 | 0.665205 |
| State | Ohio | -1.9796 | -3.1991 | -0.7447 | 0.001747 |
| State | Oklahoma | 3.1944 | -0.4004 | 6.919 | 0.082182 |
| State | Oregon | 2.4208 | 1.3314 | 3.5219 | 0.000012 |
| State | Pennsylvania | -1.3884 | -2.3453 | -0.422 | 0.004954 |
| State | South Carolina | -0.6015 | -1.4823 | 0.2871 | 0.174166 |
| State | Tennessee | -1.0468 | -2.6781 | 0.6118 | 0.214697 |
| State | Texas | -0.1855 | -1.7862 | 1.4412 | 0.821865 |
| State | Virginia | -1.4115 | -5.7839 | 3.1638 | 0.539095 |
| State | Washington | 0.5513 | -2.1279 | 3.3037 | 0.689905 |
| State | West Virginia | -2.7357 | -4.5156 | -0.9227 | 0.003243 |
| State | Wisconsin | 0.3299 | -0.9971 | 1.6746 | 0.627836 |
| Urbanization | Metropolitan | -0.4846 | -1.0019 | 0.0354 | 0.067697 |
| Urbanization | Nonmetropolitan | -0.3723 | -1.0628 | 0.323 | 0.293184 |
| Age Groups | 65-74 years | -1.0307 | -2.3079 | 0.2633 | 0.117995 |
| Age Groups | 75-84 years | -1.2624 | -2.0997 | -0.418 | 0.003455 |
| Age Groups | 85+ years | -0.2009 | -0.9995 | 0.604 | 0.623632 |

Note: The analysis period specifies the interval summarized by each AAPC. Urbanization-specific AAPCs cover 1999–2020; all other AAPCs cover 1999–2023.

Supplementary Table 5. Sensitivity analysis comparing AAPCs for the full study period (1999–2023) and the restricted pre-pandemic period (1999–2019).

| Metric | Measure | AAPC (1999–2023), % (95% CI) | AAPC (1999–2019), % (95% CI) |
| --- | --- | --- | --- |
| Census Region | Midwest | −0.94 (−1.61, −0.27) | −0.82 (−1.38, −0.25) |
|  | Northeast | −1.46 (−2.19, −0.73) | −1.87 (−2.87, −0.85) |
|  | South | −0.70 (−1.54, 0.15) | −0.86 (−1.72, 0.01) |
|  | West | 0.40 (−0.52, 1.33) | 0.27 (−0.64, 1.20) |
| Race | Hispanic | 2.58 (0.93, 4.25) | 1.82 (0.64, 3.01) |
|  | NH Black | −1.69 (−3.09, −0.26) | −1.39 (−2.39, −0.39) |
|  | NH Other | 0.73 (−1.35, 2.86) | 0.94 (−1.45, 3.40) |
|  | NH White | −0.56 (−1.25, 0.13) | −0.58 (−1.18, 0.03) |
| Sex | Both | −0.73 (−1.39, −0.06) | −0.78 (−1.31, −0.24) |
|  | Female | −0.55 (−1.20, 0.10) | −0.40 (−0.95, 0.15) |
|  | Male | −1.17 (−2.09, −0.25) | −1.30 (−2.01, −0.60) |
| Urbanization | Metropolitan | −0.48 (−1.00, 0.04) | −0.68 (−1.20, −0.14) |
|  | Nonmetropolitan | −0.37 (−1.06, 0.32) | −0.55 (−1.30, 0.21) |
| Age Groups | 65–74 years | −1.03 (−2.31, 0.26) | −1.82 (−3.53, −0.09) |
|  | 75–84 years | −1.26 (−2.10, −0.42) | −1.16 (−1.84, −0.48) |
|  | ≥85 years | −0.20 (−1.00, 0.60) | −0.29 (−1.00, 0.43) |

AAPC for urbanization analyses limit to 1999-2020 rather than 1999-2023 due to data limits.

Supplementary table 6. Comparison of race- and ethnicity-specific age-adjusted mortality rates from the bridged-race and single-race CDC WONDER datasets during the overlapping period 2018 to 2020.

| Group | Year | Bridged-race AAMR | Single-race AAMR | Absolute difference | Relative difference (%) |
| --- | --- | --- | --- | --- | --- |
| Hispanic | 2018 | 9.1096 | 9.1096 | +0.0000 | +0.00 |
| Hispanic | 2019 | 8.2312 | 8.2312 | +0.0000 | +0.00 |
| Hispanic | 2020 | 10.2497 | 10.2497 | +0.0000 | +0.00 |
| NH White | 2018 | 12.7825 | 12.8947 | +0.1122 | +0.88 |
| NH White | 2019 | 13.0193 | 13.0438 | +0.0245 | +0.19 |
| NH White | 2020 | 13.6739 | 13.6984 | +0.0245 | +0.18 |
| NH Black | 2018 | 11.6083 | 11.7529 | +0.1446 | +1.25 |
| NH Black | 2019 | 12.0035 | 12.1126 | +0.1091 | +0.91 |
| NH Black | 2020 | 13.8946 | 14.0023 | +0.1077 | +0.78 |
| NH Other | 2018 | 6.4158 | 6.0240 | -0.3918 | -6.11 |
| NH Other | 2019 | 5.7448 | 5.8827 | +0.1379 | +2.40 |
| NH Other | 2020 | 6.0686 | 6.0936 | +0.0250 | +0.41 |

AAMRs are expressed per 100,000 persons and were calculated for U.S. residents aged 65 years or older with Alzheimer disease (ICD-10 G30) and malignant neoplasms (ICD-10 C00-C97) both recorded on the death certificate, with underlying cause retained as All Causes. Absolute difference = single-race AAMR - bridged-race AAMR. Relative difference = absolute difference / bridged-race AAMR x 100. NH Other comprised non-Hispanic American Indian or Alaska Native plus Asian or Pacific Islander in the bridged-race dataset, and non-Hispanic American Indian or Alaska Native, Asian, Native Hawaiian or Other Pacific Islander, and more than one race in the single-race dataset.
